# Exploring the Causal Relationship Between Blood Metabolites and Chronic Periodontitis: Insights From Genetic Causal Analysis

**DOI:** 10.1111/jcmm.70938

**Published:** 2025-10-31

**Authors:** Weilun Cai, Huaxuan Zhao, Panpan Wang, Xiao Chen, Yumeng Yang, Hongle Wu, Zehao Chen, Fuchun Fang, Wei Qiu

**Affiliations:** ^1^ Department of Stomatology, Nanfang Hospital Southern Medical University Guangzhou China; ^2^ Department of Periodontology Guanghua School and Hospital of Stomatology, Sun Yat‐sen University Guangzhou China; ^3^ Stomatological Hospital, School of Stomatology Southern Medical University Guangzhou China

**Keywords:** chronic periodontitis, genome‐wide association study, Mendelian randomisation, metabolic pathways, metabolites

## Abstract

To investigate the potential bidirectional causal relationship between specific blood metabolites and chronic periodontitis using Mendelian randomisation (MR). A two‐sample bidirectional MR analysis was conducted. Data on 1400 blood metabolites were obtained from a genome‐wide association study involving 8299 participants. Chronic periodontitis data were sourced from the FinnGen consortium, comprising 4784 cases and 272,252 controls. The primary analysis involved the inverse‐variance weighted (IVW) method. Furthermore, a metabolic pathway enrichment analysis was conducted on the metabolites identified by the IVW method. Following IVW and sensitivity analyses, 60 blood metabolites were found to be associated with chronic periodontitis. Metabolic pathway analysis suggested that these metabolites may influence chronic periodontitis through four pathways: pyrimidine metabolism, glycine, serine and threonine metabolism, arginine and proline metabolism and riboflavin metabolism. Reverse MR analysis indicated that chronic periodontitis could alter the levels of 47 blood metabolites. Pathway analysis revealed that chronic periodontitis might affect blood metabolite levels through four pathways: vitamin B6 metabolism, ether lipid metabolism, glycerophospholipid metabolism and caffeine metabolism. This study provides new insights into the pathogenesis of periodontitis, identifying potential blood biomarkers for early detection and monitoring.

## 1 Introduction

Periodontitis is a prevalent chronic inflammatory disease affecting nearly 50% of the global population. In its advanced stages, it can lead to tooth mobility and tooth loss, thereby impacting oral function, aesthetics and overall health [1, 2, 3]. Consequently, early identification and prevention of periodontitis are essential.

Metabolites are substances produced during the breakdown of food, chemicals or tissues in the body, generating energy and maintaining physiological functions. Metabolomics, the comprehensive measurement of all metabolites, can be used to detect changes in metabolite levels and composition within cells, tissues or body fluids, resulting from genetic variations or physiological and pathological conditions [4, 5]. In recent years, a significant association between systemic metabolic status and oral health has been demonstrated. For instance, obesity is linked to not only various systemic diseases but also to an increased severity of oral inflammation. Studies have shown that hydroxyproline levels are significantly elevated in the saliva and plasma of obese children and are associated with exacerbated gingivitis [6]. These findings suggest that systemic metabolic status may influence the progression of periodontitis through similar mechanisms.

Although the pathogenesis of periodontitis is incompletely understood, studies have reported an association with metabolic abnormalities [7]. Metabolomic analysis has enhanced our understanding of the complex pathways driving the pathogenesis of periodontal disease by identifying key metabolites as biomarkers. This approach has broadened our perspective on microbe‐host interactions within the disease mechanism. A deeper understanding of these interactions is crucial for uncovering the pathogenesis of periodontal disease and its potential impact on overall health [8]. Screening blood biomarkers can be used to identify potential changes in complex multifactorial biochemical pathways, offering insights into the causal role of metabolites in disease aetiology [9]. This approach may facilitate the early detection and prevention of periodontitis by identifying abnormalities before the appearance of clinical symptoms [10, 11]. However, comprehensive and systematic studies evaluating the causal relationship between blood metabolites and chronic periodontitis remain limited. A recent Mendelian randomisation (MR) study demonstrated a significant association between periodontitis and specific metabolites such as 25‐hydroxyvitamin D (25(OH)D) in cross‐sectional research; however, their MR analyses have not consistently supported these findings [12]. Large‐scale metabolomic genome‐wide association studies (GWAS) on the MR of chronic periodontitis are yet to be conducted.

MR analysis utilises genetic variants as instrumental variables to investigate potential causal relationships between specific exposures and disease outcomes [13]. Compared to traditional observational studies, MR analysis provides significant advantages by leveraging genetic variants that are determined at conception and thus remain unaffected by environmental exposures, lifestyle factors or disease progression [14]. This approach effectively minimises confounding factors and reverse causation biases, making MR particularly suitable for investigating conditions such as the relationship between blood metabolites and chronic periodontitis, where strong associations have been observed but causal relationships remain unclear. Therefore, the application of MR analysis in this study is scientifically justified and necessary, overcoming limitations inherent in conventional study designs and providing robust theoretical support for clarifying the potential causal role of blood metabolites in the pathogenesis of periodontitis.

Given the limited understanding of the causal relationships between blood metabolites and periodontitis, this study is the first to utilise a dataset of 1400 blood metabolites as exposures to systematically evaluate the causal relationships between blood metabolites and chronic periodontitis, providing a theoretical basis for identifying periodontitis‐related metabolites. We believe that this study is not only of significant importance in the field of periodontitis but will also attract the attention of readers interested in the connections between periodontitis, systemic diseases and metabolism‐related issues.

## 2 Materials and Methods

### 2.1 Study Design

**FIGURE 1:**
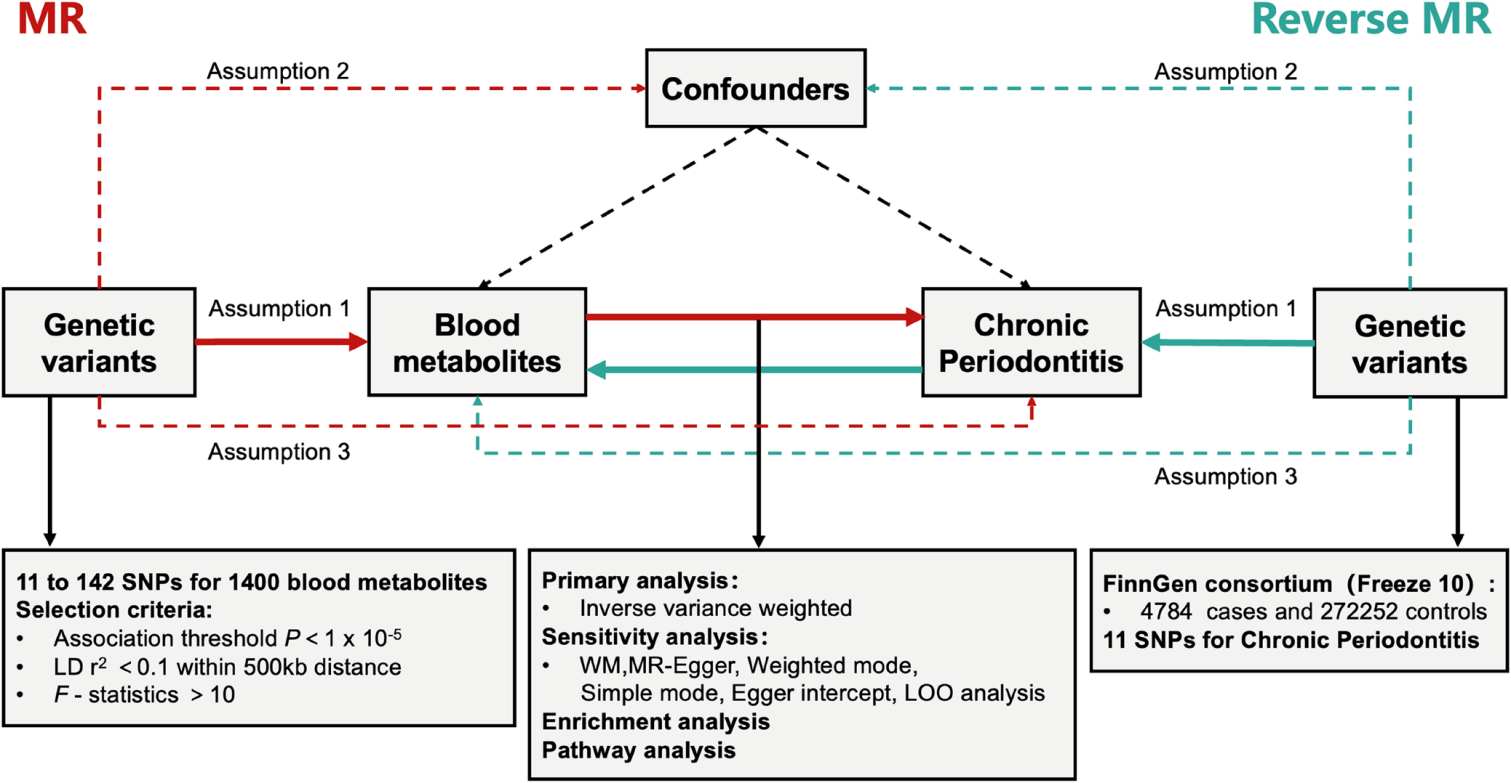
Overview of the Mendelian randomisation (MR) study. Assumption 1: genetic variants are robustly associated with exposure. Assumption 2: genetic variants are not associated with confounders. Assumption 3: genetic variants affect the outcome only through the exposure of interest. LD, linkage disequilibrium; LOO, leave‐one‐out; SNPs, single nucleotide polymorphisms; WM, weighted median.

We conducted a two‐sample MR analysis to investigate the causal relationship between 1400 blood metabolites and chronic periodontitis. This analytical method was based on three fundamental assumptions [15]:

1. The genetic variants are strongly associated with the exposure.
2. The genetic variants are not associated with any confounding factors.
3. The genetic variants influence the outcome exclusively through the exposure (Table S1 and Figure 1).

### 2.2 Data Sources

Data used in this study are publicly accessible, and they were obtained legally. The study adhered to the strengthening the reporting of observational studies in epidemiology using Mendelian randomisation (STROBE‐MR) guidelines to ensure the integrity of research [16].

#### 2.2.1 GWAS Data for Blood Metabolites

Our study utilised summary statistics from a GWAS on 1400 human blood metabolites, involving 8299 individuals of European ancestry from the Canadian Longitudinal Study on Aging. This dataset included 1091 metabolites and 309 metabolite ratios. Among the 1091 blood metabolites, 850 were classified into eight super pathways: amino acids, lipids, nucleotides, carbohydrates, peptides, energy, cofactors and vitamins and xenobiotics. The remaining 241 metabolites are currently unclassified [17]. Detailed information on these data is available in Table S2 and the original publications.

#### 2.2.2 GWAS Data for Chronic Periodontitis

Data for chronic periodontitis were obtained from the R10 release of the FinnGen consortium, which included 4784 cases and 272,252 controls [18]. Periodontitis was defined according to the International Statistical Classification of Diseases and Related Health Problems, Tenth Revision (ICD‐10) code K05.3.0 and K05.31.

#### 2.2.3 Genetic Instrumental Variable Selection

We selected single nucleotide polymorphisms (SNPs) that were strongly associated with the metabolites, using a significance threshold of *p* < 1 × 10^−5^. In MR studies, a relatively relaxed statistical threshold is applied when there are insufficient genome‐wide significant SNPs for the exposure. We performed linkage disequilibrium pruning with *r* ^2^ < 0.1 and a kilobase (kb) distance of 500, focusing on the European population. The *F*‐statistic was used to assess the strength of the genetic instruments for each metabolite; SNPs with an *F*‐statistic < 10 were considered weak instruments. Consequently, we only selected SNPs with *F*‐statistic > 10 as instrumental variables (Figure 1).

### 2.3 Metabolite Set Enrichment Analysis and Metabolic Pathway Analysis

To elucidate the potential metabolic mechanisms underlying the identified metabolites and to explore their common biological basis with chronic periodontitis, we performed metabolite set enrichment analysis (MSEA) and metabolic pathway analysis using MetaboAnalyst 6.0 [19] (https://www.metaboanalyst.ca/). Specifically, significant metabolites identified from bidirectional MR analyses (60 metabolites in MR analysis and 47 metabolites in reverse MR analysis; inverse‐variance weighted (IVW) method, *p* < 0.05) were matched within the Human Metabolome Database (HMDB). Subsequently, MSEA was performed using the Over Representation Analysis (ORA) method based on the RaMP‐DB (Relational Database of Metabolomic Pathways) database to identify significantly enriched metabolite sets. Furthermore, metabolic pathway analysis using the KEGG database was carried out to annotate and interpret the pathways involving these significant metabolites, thereby identifying key metabolic pathways potentially involved in the development and progression of chronic periodontitis (significance threshold set at *p* < 0.05) [20].

### 2.4 Statistical Analysis

The main analyses were conducted using R (version 4.3.1) with the TwoSampleMR package (version 0.5.8). In the primary MR analysis, we employed the IVW method to estimate causal effects [21, 22], as it is the most robust method when all genetic variants are valid instruments. We identified metabolites significantly associated with chronic periodontitis (*p* < 0.05) and applied a false discovery rate (FDR) threshold of < 0.2 to exclude false positives. Sensitivity analyses were performed using several MR methods, including MR‐Egger, weighted median, simple mode, weighted mode and leave‐one‐out analysis. Pleiotropy was assessed using the MR‐Egger intercept.

## 3 Results

### 3.1 Characteristics of the Selected SNPs

According to the instrumental variable selection criteria, we used between 11 and 142 SNPs as instrumental variables for the 1400 blood metabolites associated with chronic periodontitis. In the reverse MR analysis, 11 SNPs were selected as instrumental variables for chronic periodontitis (Figure 1). All instrumental variables had *F*‐statistics > 10, indicating the robustness of the MR analysis for chronic periodontitis‐related blood metabolites, with no weak instruments present. A bidirectional two‐sample MR analysis was conducted to explore the causal relationship between these metabolites and chronic periodontitis.

### 3.2 Causal Effect of Blood Metabolites on Chronic Periodontitis

**FIGURE 2:**
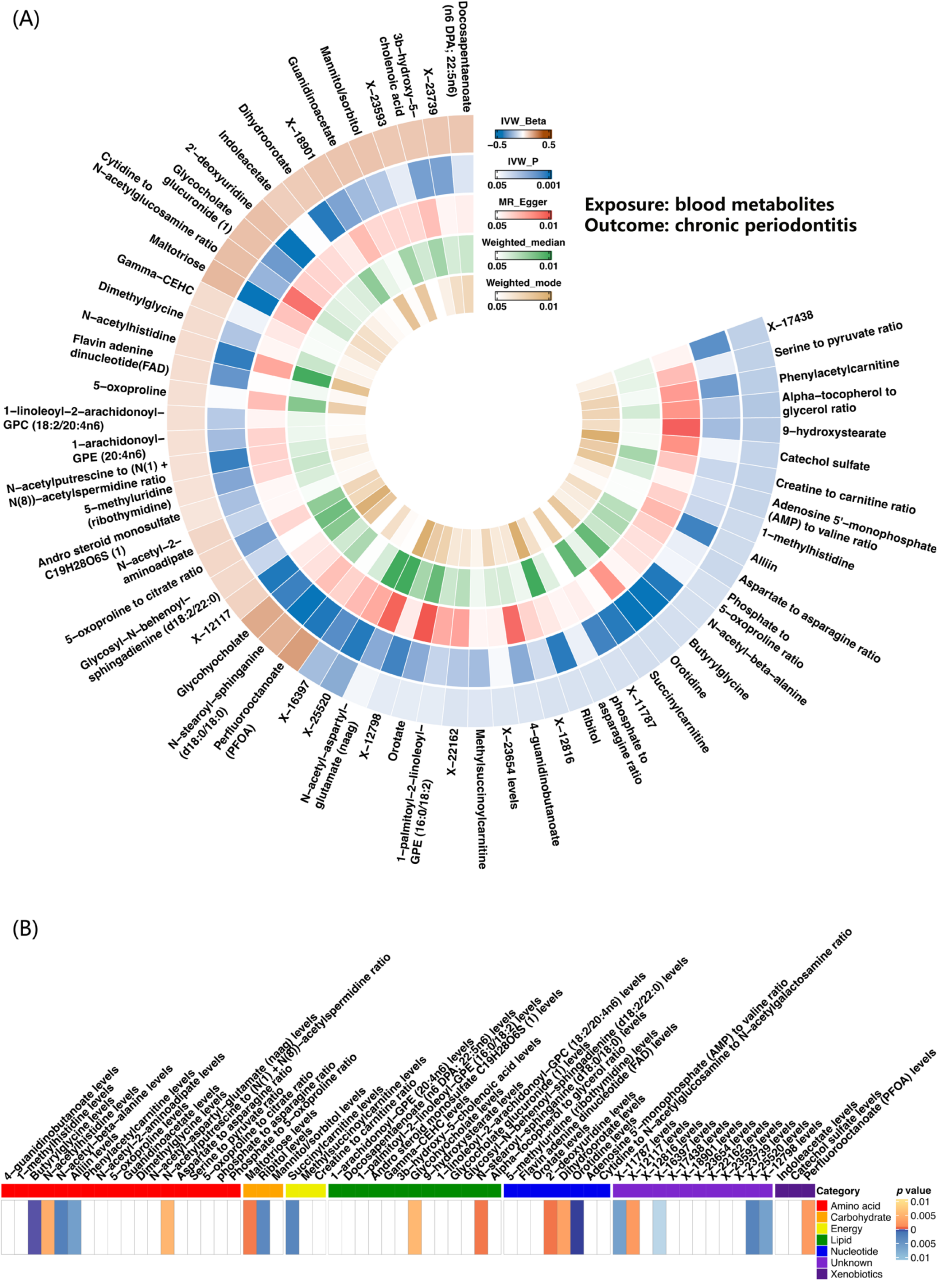
Circular heatmap results and metabolite classification heatmap results of blood metabolites on chronic periodontitis based on MR analysis. (A) Circular heatmap plot illustrating the causal effects of blood metabolites on chronic periodontitis, based on MR analysis. From outside to inside, the *p* values of inverse variance weighted (IVW) Beta, IVW, MR‐Egger, weighted median and weighted mode are shown, with data from the FinnGen consortium. The colour of each metabolite in this circular heatmap represents the *p* value of the MR method corresponding to its respective layer. Gradient blue indicates a significant causal association (*p* < 0.05). (B) Heatmap plot showing the causal associations between blood metabolites and chronic periodontitis. The *x*‐axis shows significant blood metabolites, grouped into seven different categories. The y‐axis shows chronic periodontitis. The coloured cells denote metabolite‐phenotype associations with significant MR based on the IVW method (*p* < 0.01). Red cells represent increased risk between each metabolite and phenotype, while blue cells represent decreased risk.

Using the IVW method, we initially identified 70 blood metabolites significantly associated with chronic periodontitis. Through sensitivity analysis, 60 metabolites were confirmed, including 37 known metabolites, 10 metabolite ratios and 12 unknown metabolites (Tables S3 and S4; Figure 2A; Figure S1A). These metabolites were categorised based on their chemical properties into amino acids, carbohydrates, energy, lipids, nucleotides, xenobiotics, cofactors and vitamins (Figure 2B). The five most significant blood metabolites were butyrylglycine (*p* = 0.0000844, odds ratio [OR] = 0.91, 95% confidence interval [CI]: 0.87–0.95), orotidine (*p* = 0.0002, OR = 0.91, 95% CI: 0.87–0.95), 2′‐deoxyuridine (*p* = 0.0007, OR = 1.14, 95% CI: 1.05–1.24), N‐stearoyl‐sphinganine (d18:0/18:0) (*p* = 0.0007, OR = 1.18, 95% CI: 1.07–1.30) and maltotriose (*p* = 0.0008, OR = 1.16, 95% CI: 1.06–1.27) (Table 1 and Table S5; Figure S2A). Additionally, the MR‐Egger intercept indicated a low risk of horizontal pleiotropy and the leave‐one‐out analysis showed no SNPs with a high influence that could bias the overall effect estimate.

**TABLE 1:** Top five significant metabolites identified in MR and reverse MR analysis with effect estimates.

Analysis	Exposure	Outcome	SNP	*p*	OR (95% CI)
MR	Butyrylglycine	Chronic periodontitis	47	8.44E‐05	0.91 (0.87–0.95)
	Orotidine	Chronic periodontitis	33	< 0.001	0.91 (0.87–0.95)
	2′‐deoxyuridine	Chronic periodontitis	34	< 0.001	1.14 (1.05–1.24)
	N‐stearoyl‐sphinganine	Chronic periodontitis	13	< 0.001	1.18 (1.07–1.30)
	Maltotriose	Chronic periodontitis	23	< 0.001	1.16 (1.06–1.27)
Reverse MR	Chronic periodontitis	Furaneol sulfate	11	3.73E‐05	1.24 (1.12–1.37)
	Chronic periodontitis	N‐acetyl‐2‐aminoadipate	11	0.001	1.15 (1.05–1.26)
	Chronic periodontitis	Pyridoxal	11	0.002	0.86 (0.79–0.94)
	Chronic periodontitis	Glycerophosphoethanolamine	11	0.006	0.88 (0.81–0.96)
	Chronic periodontitis	Guanidinoacetate	11	0.007	0.88 (0.81–0.96)

Following FDR correction (*p* < 0.2), two blood metabolites, orotidine (*p* = 0.10) and butyrylglycine (*p* = 0.14), remained significantly associated with chronic periodontitis, suggesting that both metabolites may reduce the risk of chronic periodontitis (Table S3; Figure S2C).

### 3.3 Causal Effect of Chronic Periodontitis on Blood Metabolites

**FIGURE 3:**
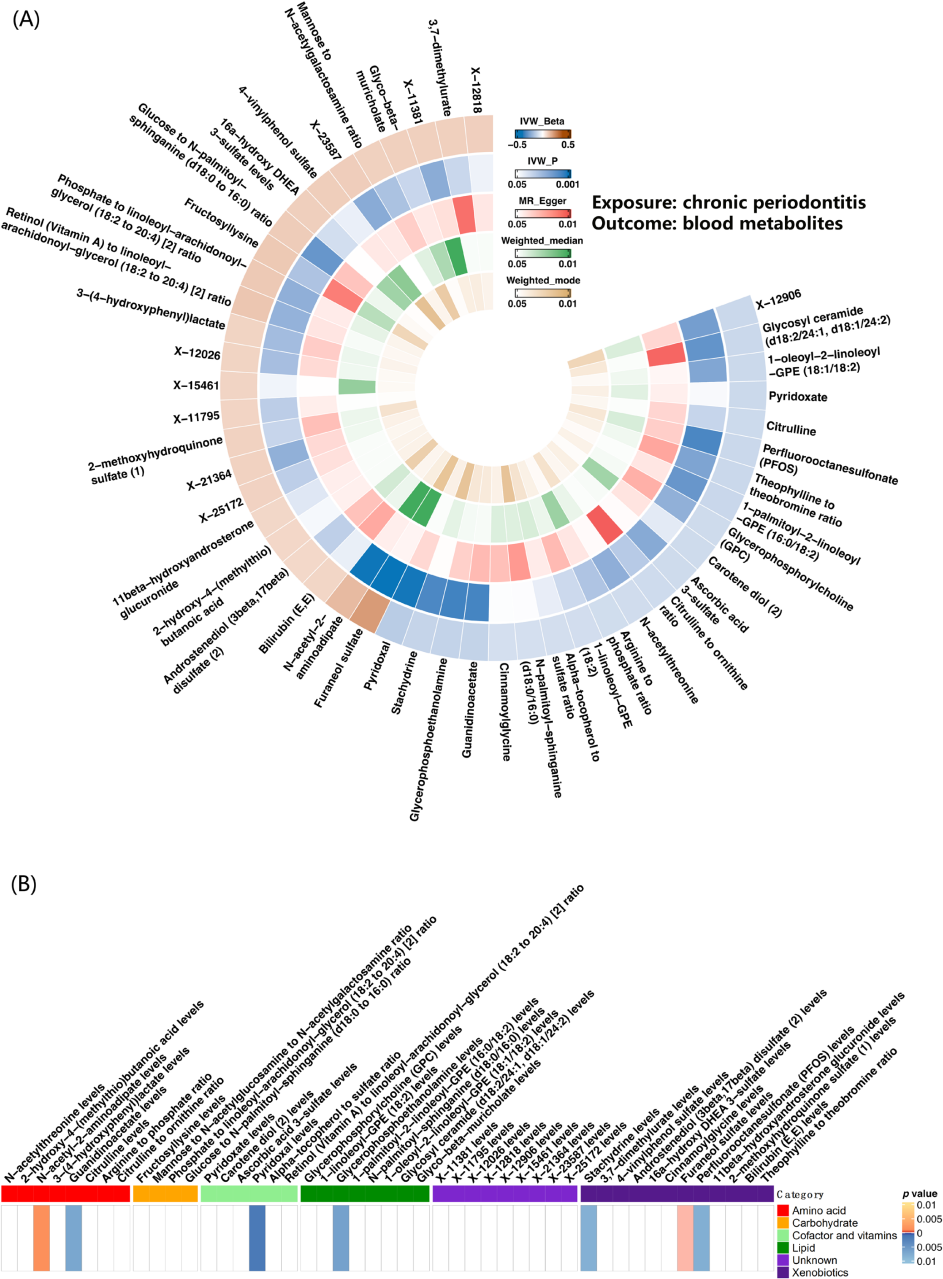
Circular heatmap results and metabolite classification heatmap results of reverse MR analysis of chronic periodontitis on blood metabolites. (A) Circular heatmap plot showing the causal effects of chronic periodontitis on blood metabolites, based on reverse MR analysis. (B) Heatmap plot of causal associations between chronic periodontitis and blood metabolites. We identified six additional distinct categories.

Using the IVW method, we initially found that chronic periodontitis may cause changes in the levels of 47 blood metabolites. Sensitivity analysis confirmed these 47 metabolites, including 30 known metabolites, 8 metabolite ratios and 9 unknown metabolites (Tables S6 and S7; Figure 3A and Figure S1B). These were classified into amino acids, carbohydrates, cofactors and vitamins, lipids and xenobiotics (Figure 3B). The five most significant blood metabolites were furaneol sulfate (*p* = 0.0000372, OR = 1.24, 95% CI: 1.12–1.37), N‐acetyl‐2‐aminoadipate (*p* = 0.001, OR = 1.15, 95% CI: 1.05–1.26), pyridoxal (*p* = 0.001, OR = 0.86, 95% CI: 0.79–0.94), glycerophosphoethanolamine (*p* = 0.0064, OR = 0.88, 95% CI: 0.81–0.96) and guanidinoacetate (*p* = 0.007, OR = 0.88, 95% CI: 0.81–0.96) (Table 1 and Table S5; Figure S2B).

After FDR correction (*p* < 0.2), chronic periodontitis significantly affected the level of only furaneol sulfate (*p* = 0.05). Additionally, the MR‐Egger intercept indicated a low risk of horizontal pleiotropy and the leave‐one‐out analysis showed no SNPs with a high influence that could bias the overall effect estimate. This suggested that chronic periodontitis leads to an increase in the blood level of furaneol sulfate (Table S6; Figure S2D).

### 3.4 MSEA and Metabolic Pathway Analysis

**FIGURE 4:**
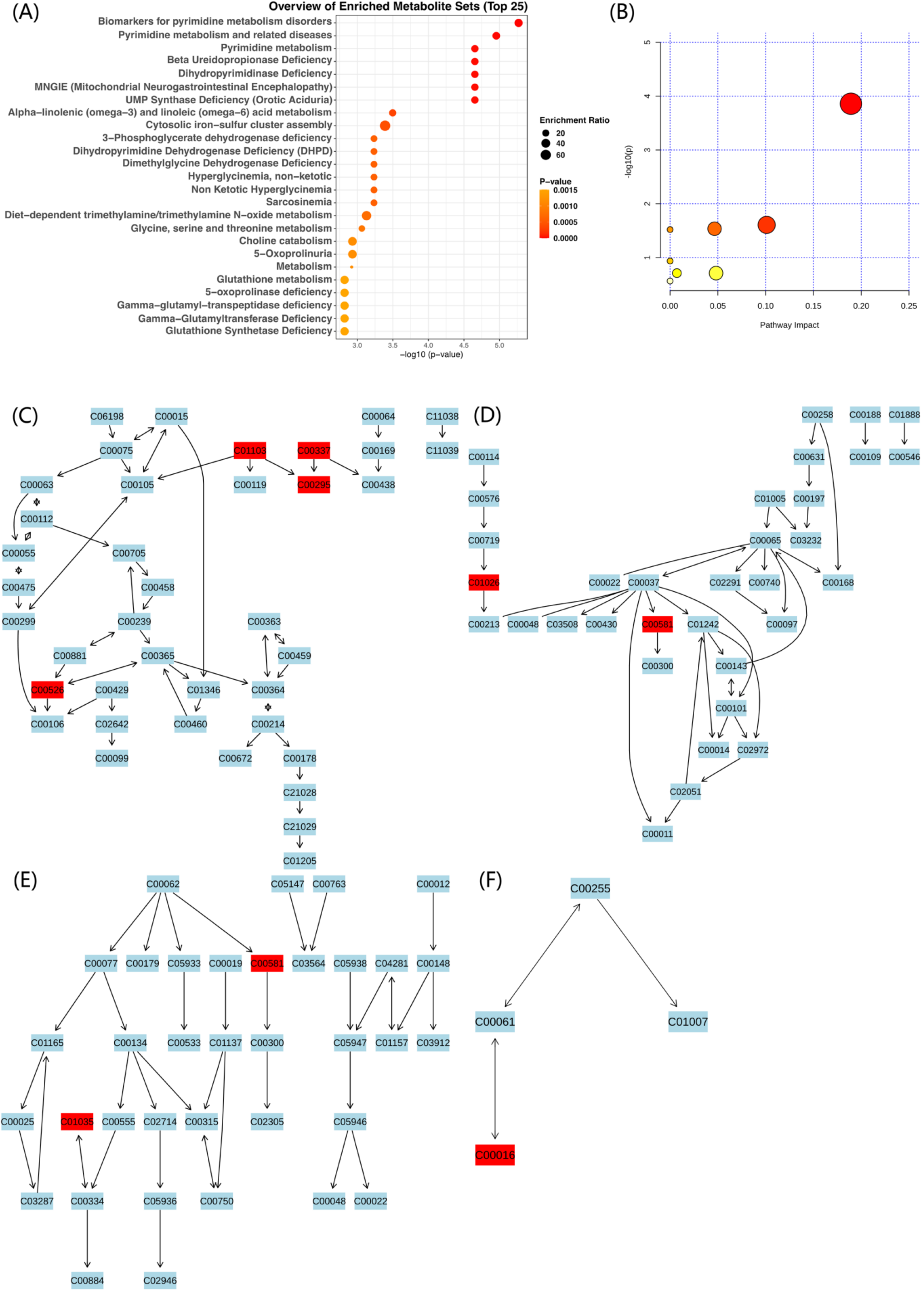
MSEA and metabolic pathway analysis of significant blood metabolites identified by MR. (A) Top 25 enriched metabolite sets identified by MSEA using significant blood metabolites (*p* < 0.05) from the MR analysis. (B) The metabolome view provides an overview of all significantly impacted pathways identified through pathway analysis. Each dot represents a metabolic pathway, with the size indicating the enrichment ratio and the colour representing the *p* value. Larger and darker dots indicate higher enrichment significance. (C) Pyrimidine metabolism. (D) Glycine, serine and threonine metabolism. (E) Arginine and proline metabolism. (F) Riboflavin metabolism. The panels (C–F) show detailed pathway diagrams highlighting key metabolic pathways. MSEA was based on the RaMP database, and pathway analysis was conducted using the KEGG database. The significance threshold was set at *p* < 0.05. In the pathway diagrams, light blue nodes indicate background metabolites not present in the input data, while red nodes indicate significantly enriched metabolites.

**FIGURE 5:**
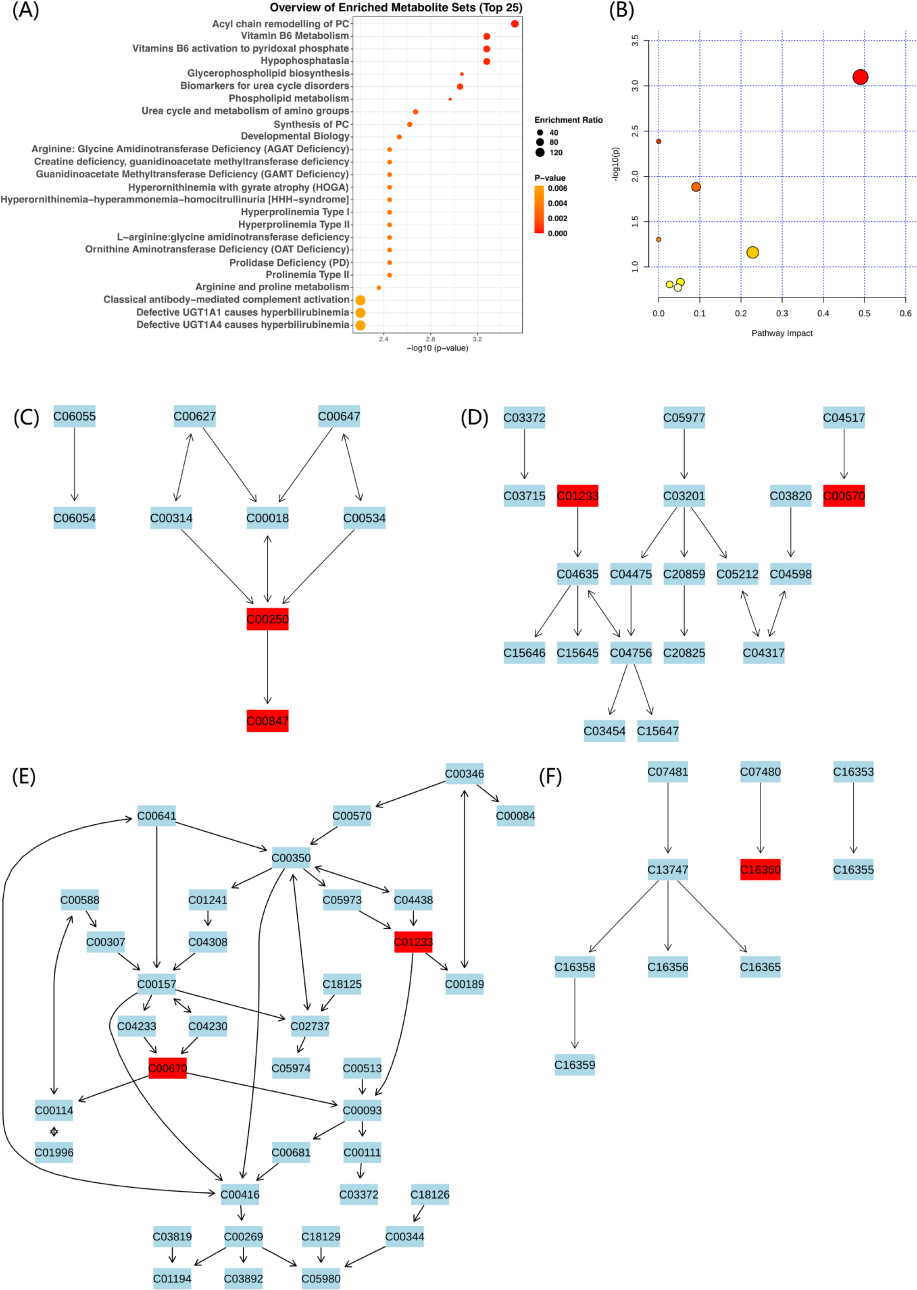
MSEA and metabolic pathway analysis of significant blood metabolites identified by reverse MR. (A) Top 25 enriched metabolite sets identified by MSEA using significant blood metabolites (*p* < 0.05) from the reverse MR analysis. (B) The metabolome view illustrates significantly impacted pathways identified through KEGG‐based pathway analysis. Each dot represents a metabolic pathway, with dot size indicating the enrichment ratio and colour representing the *p* value. Larger and darker dots reflect higher statistical significance. (C) Vitamin B6 metabolism. (D) Ether lipid metabolism. (E) Glycerophospholipid metabolism. (F) Caffeine metabolism. The panels (C–F) show detailed pathway diagrams highlighting key metabolic pathways. MSEA was conducted using the RaMP database, and pathway analysis was based on the KEGG database, with a significance threshold of *p* < 0.05. All analyses were performed via the MetaboAnalyst 6.0. In the pathway diagrams, light blue nodes indicate background metabolites not present in the input data, while red nodes represent statistically significant metabolites included in the data.

We conducted MSEA and metabolic pathway analysis on the 60 and 47 blood metabolites identified in our study. In the MSEA, using the RaMP‐DB database, we identified 83 significant metabolic enrichment pathways (*p* < 0.05) related to chronic periodontitis, primarily involving pyrimidine metabolism, amino acid metabolism, nucleotide metabolism and energy metabolism pathways (Figure 4A and Figure S3). In the reverse MR analysis, 91 significant metabolic enrichment pathways (*p* < 0.05) were identified, including vitamin metabolism, lipid metabolism and amino acid metabolism, and other biologically relevant metabolic pathways (Figure 5A and Figure S4).

In the metabolic pathway analysis, we identified four potential metabolic pathways involved in the development of chronic periodontitis. Orotidine, 2′‐deoxyuridine, orotate and dihydroorotate are involved in pyrimidine metabolism; guanidinoacetate and dimethylglycine in glycine, serine and threonine metabolism; guanidinoacetate and 4‐guanidinobutanoate in arginine and proline metabolism; and flavin adenine dinucleotide in riboflavin metabolism (*p* < 0.05) (Table 2; Figure 4B–F and Figure S5). In the reverse MR analysis, four potential metabolic pathways were also identified, suggesting that chronic periodontitis may influence the levels of pyridoxal and glycerophosphoethanolamine through pathways, such as vitamin B6 metabolism, ether lipid metabolism, glycerophospholipid metabolism and caffeine metabolism (*p* < 0.05) (Table 2; Figure 5B–F and Figure S6).

**TABLE 2:** Significant metabolic pathways involved in chronic periodontitis from MR and reverse MR analysis.

Analysis	Pathway name	Involved metabolites	*p*
MR	Pyrimidine metabolism	(S)‐Dihydroorotate Orotidine 5′‐phosphate Deoxyuridine Orotate	1.38E‐04
	Glycine, serine and threonine metabolism	Guanidinoacetate N‐Dimethylglycine	0.025
	Arginine and proline metabolism	Guanidinoacetate 4‐Guanidinobutanoate	0.029
	Riboflavin metabolism	Flavin adenine dinucleotide	0.030
Reverse MR	Vitamin B6 metabolism	Pyridoxal 4‐Pyridoxate	7.98E‐04
	Ether lipid metabolism	sn‐Glycero‐3‐phosphoethanolamine sn‐Glycero‐3‐phosphocholine	0.005
	Glycerophospholipid metabolism	sn‐Glycero‐3‐phosphocholine sn‐Glycero‐3‐phosphoethanolamine	0.013
	Caffeine metabolism	3,7‐Dimethyluric acid	0.049

## 4 Discussion

This study used MR analysis to explore the causal relationship between blood metabolites and chronic periodontitis, identifying 60 metabolites related to the disease. Additionally, a reverse MR analysis was performed, which suggested that chronic periodontitis may cause alterations in the levels of 47 metabolites. These findings indicated that certain blood metabolites could serve as potential biomarkers or therapeutic targets for chronic periodontitis, offering valuable insights into the genetic correlations between metabolites and this condition.

Recent research has highlighted the influence of metabolites and metabolic processes on disease risk. Understanding the causal role of metabolites in disease aetiology could pave the way for novel treatment options. While current literature has suggested a correlation between metabolic disorders and chronic periodontitis [5], a definitive causal role of metabolites in periodontitis has not been established. Based on previous metabolite‐related GWAS, we designed this study to systematically evaluate the causal relationships between metabolites and periodontitis. To our knowledge, this is the first MR study focusing on large‐scale blood metabolite GWAS data in relation to chronic periodontitis, aiming to elucidate the relationship between periodontitis pathogenesis and metabolism and to identify new targets for early identification and prevention of the disease.

Our MR analysis demonstrated that higher blood levels of 2′‐deoxyuridine, N‐stearoyl‐sphinganine (d18:0/18:0) and maltotriose are associated with an increased risk of chronic periodontitis. This suggests that a genetic predisposition to elevated levels of these metabolites increases the risk of developing the disease. N‐stearoyl‐sphinganine (d18:0/18:0), a sphingolipid metabolite, has also been implicated in other studies. A cross‐sectional study on plasma 3‐carboxy‐4‐methyl‐5‐propyl‐2‐furanpropanoic acid (CMPF) levels in 922 participants found that higher levels were associated with reduced gingival inflammation and a lower risk of periodontitis, suggesting a potential protective role of CMPF in periodontal health. However, the study's design was limited by methodological flaws, making it more suitable for exploring potential associations than for establishing causality. Through MR, which is not subject to reverse causation and confounding factors, we demonstrated that higher N‐stearoyl‐sphinganine (d18:0/18:0) levels are predictive of an increased risk of chronic periodontitis [23]. Since CMPF and N‐stearoyl‐sphinganine (d18:0/18:0) are both lipid metabolites, they may be linked through common metabolic pathways, similar biological effects or dietary habits. Further research is necessary to clarify these associations and mechanisms. Regarding 2′‐deoxyuridine, a pyrimidine metabolism product, it primarily participates in DNA synthesis and repair processes. A metabolomic study on saliva, plasma and multifluid samples in periodontal disease found an association between 2′‐deoxyuridine levels and periodontitis, suggesting that changes in plasma 2′‐deoxyuridine levels may be related to the onset and progression of periodontitis [24]. Given its key role in cell proliferation and DNA metabolism, it is hypothesised that 2′‐deoxyuridine may influence chronic periodontitis development by affecting cell proliferation and inflammatory responses. Maltotriose is a product of carbohydrate metabolism and is closely associated with glucose metabolism. Its levels increase under high‐glucose, hypoxic or inflammatory conditions. Studies in inflammatory tissues and islet cells have shown that maltotriose may serve as a marker of metabolic dysregulation [25], with its elevation potentially reflecting abnormal glucose metabolism and being associated with the development and progression of chronic inflammation and periodontitis. Although direct research on the roles of these metabolites in periodontitis is limited, further studies are needed to investigate their involvement in metabolic disorders and inflammatory responses. These findings offer new directions for understanding the metabolic mechanisms underlying periodontitis and may also have broader implications for overall health. This study underscores the critical role of metabolic regulation in the pathogenesis and management of chronic inflammatory diseases. By identifying blood metabolites with a causal impact on chronic periodontitis, our findings support the concept that targeting metabolic pathways may enable early intervention, prevention and treatment of periodontitis and other metabolic‐related chronic diseases. Previous studies have demonstrated that plasma advanced glycation end‐products (AGEs) aggravate periodontal tissue destruction by activating the RAGE signalling pathway in gingival fibroblasts (GFs). This activation significantly upregulates the expression of proinflammatory cytokines such as TNF‐α and IL‐6, induces cellular apoptosis and oxidative stress and ultimately exacerbates periodontal inflammation and tissue damage [26]. This mechanism further illustrates how blood metabolites may influence the development and progression of periodontitis through the regulation of inflammatory responses and cellular homeostasis. A recent study found that both salivary and plasma levels of trimethylamine N‐oxide (TMAO) were significantly elevated in patients with periodontitis and showed a positive correlation with TNF‐α and periodontal parameters, suggesting that TMAO is a promising non‐invasive inflammatory biomarker that is also associated with disease severity [27, 28]. Additionally, studies have shown that salivary and plasma levels of malondialdehyde (MDA) are significantly increased in periodontitis patients. Notably, salivary MDA levels decreased significantly after 3 months of periodontal treatment, whereas plasma MDA levels did not show significant changes, indicating that MDA is closely associated with periodontal indices and can improve with treatment [29, 30].

Elevated blood levels of two metabolites, orotidine and butyrylglycine, were found to have a protective effect against chronic periodontitis. Butyrylglycine belongs to fatty acid metabolism and is associated with energy metabolism, lipid metabolism disorders and inflammatory processes. Studies have found that butyrylglycine levels are associated with periodontitis, and both butyrylglycine and 2′‐deoxyuridine have been identified as part of the plasma metabolomic profile related to periodontitis, indicating their representative role in the plasma metabolomic characteristics of periodontitis patients. These metabolites may serve as potential biomarkers for periodontitis. Additionally, butyrylglycine is linked to fatty acid and amino acid metabolism disorders, which is consistent with chronic inflammatory conditions [24]. A recent cross‐sectional study reported that, in obese individuals, increased amino acid metabolism and decreased lipid and cofactor/vitamin metabolism might increase the risk of periodontitis [4]. This finding contrasts with our results, highlighting the need for further investigation. Orotidine is a key intermediate in the de novo pyrimidine nucleotide synthesis pathway, generated from orotic acid and phosphoribosyl pyrophosphate (PRPP). As the direct precursor of UMP (uridine monophosphate), orotidine provides the foundation for RNA and DNA synthesis. Abnormal metabolism of orotidine, typically present as orotidine 5′‐phosphate, can directly affect cell proliferation and genomic stability. A large‐scale metabolomics study with a 22‐year follow‐up demonstrated that elevated orotidine levels are significantly associated with increased risks of cardiovascular disease and coronary heart disease in patients with type 2 diabetes, suggesting its potential as a novel biomarker [31]. The underlying mechanism may involve disruption of pyrimidine metabolic homeostasis or direct participation in inflammatory pathways, thereby exacerbating vascular endothelial injury. Recent research on orotidine has primarily focused on the catalytic mechanism of orotidine‐5′‐phosphate decarboxylase (ODCase), the construction of genetically engineered strains and its application in pyrimidine biosynthesis. However, studies exploring the translational relevance of orotidine in human diseases remain limited [32].

Regarding the causal effect of chronic periodontitis on blood metabolites, our reverse MR analysis demonstrated that the condition leads to decreased blood levels of pyridoxal, glycerophosphoethanolamine and guanidinoacetate, which may weaken the body's defence mechanisms and increase susceptibility to other diseases. Pyridoxal, a form of vitamin B6, is an essential coenzyme in several enzymatic reactions involved in amino acid metabolism, neurotransmitter synthesis and red blood cell formation, primarily through its active form, pyridoxal‐5′‐phosphate [33]. Although direct studies on the relationship between pyridoxal and chronic periodontitis are limited, its role in inflammation, immune response and amino acid metabolism [34] suggests that pyridoxal may indirectly contribute to periodontitis pathogenesis. Future research should explore whether pyridoxal supplementation could improve periodontitis treatment outcomes by modulating immune response and inflammation pathways.

Conversely, chronic periodontitis led to increased N‐acetyl‐2‐aminoadipate and furaneol sulfate levels, potentially increasing the risk of systemic diseases. Furaneol sulfate, a food‐derived metabolite and N‐acetyl‐2‐aminoadipate, an intermediate product of lysine metabolism associated with amino acid metabolism, have not been specifically linked to periodontitis in existing studies. However, research suggests that vitamins and omega‐3 fatty acids may regulate inflammation associated with periodontitis, potentially playing a role in its pathophysiology. Further research is necessary to explore individualised nutritional intake and its impact on periodontitis progression and treatment [35]. We propose that modifying dietary patterns, identifying potential targets and discovering suitable drugs to lower furaneol sulfate and N‐acetyl‐2‐aminoadipate levels could help alleviate periodontitis symptoms.

With the increasing availability of GWAS data, researchers have explored periodontitis through MR studies from various perspectives. MR analysis of metabolites in relation to systemic diseases has been reported, with most studies utilising the GWAS data of 486 metabolites published in 2014 [36]. To our knowledge, MR studies on the relationship between metabolites and periodontitis are scarce. For example, an MR analysis on the causal relationship between periodontitis and 25(OH)D metabolites based on cross‐sectional observational data was conducted [12] but found inconsistent results, with no significant association between genetically elevated 25(OH)D levels and periodontitis risk. Another study also found no clear evidence of a causal relationship between circulating vitamin C and vitamin D levels and periodontitis [37]. These studies only examined the causal impact of individual metabolites on periodontitis and found no significant causal relationship. However, in a 2023 MR study involving 10 dental traits, including periodontitis, using the GWAS data of 309 metabolites published in 2014, no significant causal relationship between metabolites and periodontitis was found. In contrast, our study used the updated GWAS data of 1400 metabolites published in 2023. Taken together, in our study, both the exposure and outcome factors were derived from the most recent GWAS data, allowing us to conduct a bidirectional MR analysis on several categories of blood metabolites and chronic periodontitis, yielding significant results. Unlike previous studies limited to smaller datasets or single metabolites, our research leveraged the largest and most comprehensive human blood metabolite GWAS to date, covering 1400 metabolites and enabling large‐scale bidirectional MR analysis. This approach enhanced the statistical power and reliability of our findings and allowed for a more systematic exploration of the metabolic landscape underlying chronic periodontitis.

Research on the metabolomics of oral diseases, particularly periodontitis, remains limited and prospective studies are yet to be reported. A recent observational study reported that signs of inflammatory oral diseases, particularly periodontitis, are associated with metabolic profiles typical of inflammation [38]. In the context of oral diseases, metabolomics studies often involve saliva sample collection. Previous studies have focused on the early diagnosis of systemic diseases, including various cancers and neurodegenerative disorders, through salivary metabolites. Clinically, salivary metabolic profiles are explored for their diagnostic and prognostic applications, monitoring treatment success and personalising the treatment of both oral and systemic diseases [39]. A cross‐sectional study utilising untargeted metabolomics analysis found significantly elevated serum urea and myo‐inositol levels in generalised aggressive periodontitis patients. Conversely, glutathione, 2,5‐dihydroxybenzaldehyde, adipic acid and 2‐deoxyguanosine levels were decreased, providing insights into the underlying mechanisms of generalised aggressive periodontitis and improving our understanding of periodontitis [40]. Metabolomic analyses of various biological fluids have consistently identified common metabolites, mainly amino acids, organic acids and their derivatives, including acetate, alanine, butyrate, formate, gamma‐aminobutyric acid, lactate, propionate, phenylalanine and valine. These metabolites are believed to be associated with host and microbial responses in individuals with periodontitis, highlighting the necessity for further in‐depth research [41]. Nevertheless, research on serum metabolomics of periodontitis is still limited and further investigation is warranted. Notably, in addition to classified metabolites, our study also identified several metabolites with ‘x‐’ code (e.g., x‐12798, x‐11787) that remain unclassified and currently lack structural or functional annotation in public metabolomics databases. Although their precise biological functions are unknown, these unclassified metabolites exhibited significant causal associations with chronic periodontitis in our MR analysis. This highlights the need for future research to clarify their chemical identities and potential roles in disease pathogenesis.

In summary, this study utilised MR analysis to identify blood metabolite levels that may be significant for the early prevention of not only periodontitis but also systemic diseases. Our research provides a theoretical foundation for the role of blood metabolites in disease prevention. Despite the limited research on these metabolites in periodontal disease, future in vivo experiments and validation are essential to explore the potential of modulating blood metabolite levels for periodontitis prevention and treatment.

Our study has several limitations. First, the metabolite GWAS data used in this study included a relatively small number of genome‐wide significant SNPs available for MR analysis. We employed a relatively relaxed significance threshold for instrumental variable selection, a widely applied method, with each SNP having an *F*‐statistic greater than 10, indicating no weak instruments. Future research should utilise larger scale blood metabolite GWAS data to identify more relevant genetic loci for MR analysis. Second, our study population was predominantly of European descent, which may limit the generalisability and applicability of our findings to other populations. Future validation using GWAS data from different populations is necessary to confirm the universality of our results.

Despite these limitations, this study has notable strengths. First, it covered 1400 metabolites or metabolite ratios, representing the most comprehensive blood metabolite GWAS data to date. Second, even after multiple corrections, several blood metabolites showed significant causal associations with chronic periodontitis, effectively integrating metabolomics and genomics to identify potential biomarkers influencing the disease. Therefore, this study significantly contributes to our understanding of the potential mechanisms underlying chronic periodontitis.

## 5 Conclusion

In this study, we were the first to utilise the largest available GWAS dataset of human blood metabolites to systematically identify metabolites associated with the risk of chronic periodontitis. Our findings provide new insights into the pathogenesis of periodontitis from both metabolomic and genomic perspectives and reveal potential causal relationships between specific blood metabolites and chronic periodontitis. Notably, several identified metabolites may serve as promising blood‐based biomarkers for early screening, risk stratification and disease monitoring of chronic periodontitis. These results offer theoretical support for clinical individualised risk assessment and lay a foundation for the development of preventive and therapeutic strategies targeting metabolic pathways.

## Author Contributions

**Weilun Cai:** data curation (equal), formal analysis (equal), investigation (equal), methodology (equal), visualization (equal), writing – original draft (equal). **Huaxuan Zhao:** data curation (equal), formal analysis (equal), investigation (equal), methodology (equal), visualization (equal), writing – original draft (equal). **Panpan Wang:** project administration (equal), validation (equal), writing – review and editing (equal). **Xiao Chen:** project administration (equal), validation (equal), writing – review and editing (equal). **Yumeng Yang:** project administration (equal), validation (equal), writing – review and editing (equal). **Hongle Wu:** project administration (equal), validation (equal), writing – review and editing (equal). **Zehao Chen:** project administration (equal), validation (equal), writing – review and editing (equal). **Fuchun Fang:** conceptualization (equal), funding acquisition (equal), supervision (equal). **Wei Qiu:** conceptualization (equal), data curation (equal), supervision (equal), validation (equal).

## Ethics Statement

All of the data used in this study were obtained from previously published research and publicly available online databases. Therefore, no further ethics approval or informed consent was required.

## Conflicts of Interest

The authors declare no conflicts of interest.

## Supporting information


**Figure S1:** Forest plots for the Mendelian randomisation (MR) of the significant inverse variance weighted (IVW) estimates. (A) The forest plot of 60 positive results from the MR analysis, with blood metabolites sequentially categorised into seven categories. N indicates the number of SNPs used for the analysis of each blood metabolite, OR (95% CI) represents the odds ratio with its 95% confidence interval and *p* value indicates the level of statistical significance. (B) The forest plot of 60 positive results from the MR analysis, with blood metabolites sequentially categorised into six categories.

**Figure S2:** Scatter plot of significant MR results. (A) MR analysis scatter plot. (B) Reverse MR analysis scatter plot. (C) Causal effect of butyrylglycine and orotidine on chronic periodontitis. (D) Causal effect of chronic periodontitis on furaneol sulfate. Odds ratio point estimates and 95% confidence intervals of MR results for the causal risk blood metabolites on chronic periodontitis and chronic periodontitis on blood metabolites.

**Figure S3:** The bar chart and network chart of MSEA associated with blood metabolites related to chronic periodontitis. (A) The bar chart. (B) The network chart. Each node represents a metabolite set with its colour based on its *p* value and its size is based on fold enrichment (hits/expected) to your query. Two metabolite sets are connected by an edge if the number of their shared metabolites is over 25% of the total number of their combined metabolite sets.

**Figure S4:** The bar chart and network chart of MSEA associated with chronic periodontitis related to blood metabolites. (A) The bar chart. (B) The network chart. Each node represents a metabolite set with its colour based on its *p* value and its size is based on fold enrichment (hits/expected) to your query. Two metabolite sets are connected by an edge if the number of their shared metabolites is over 25% of the total number of their combined metabolite sets.

**Figure S5:** Metabolic pathway analysis associated with blood metabolites related to chronic periodontitis. (A)This figure illustrates the pyrimidine metabolism pathway (hsa00240), which involves de novo synthesis, degradation and nucleotide metabolism. This pathway is crucial for the synthesis of DNA and RNA in cells. Metabolites are converted through various enzyme‐catalysed reactions, eventually participating in the synthesis or degradation of nucleotides. Different nodes in the figure represent distinct metabolites in the pyrimidine metabolism, while the connections depict the relationships and conversions between these metabolites. (B)The glycine, serine and threonine metabolism pathway (hsa00260). This pathway involves the interconversion of glycine and serine, as well as the metabolism of threonine, an essential amino acid. These amino acids play crucial roles in the synthesis of nucleic acids and proteins, as well as in various metabolic pathways. (C) The arginine and proline metabolism pathway (hsa00330). This pathway involves the synthesis and degradation of arginine and proline, which play crucial roles in protein synthesis, the urea cycle and various metabolic processes. Arginine metabolism is also linked to nitric oxide production, while proline metabolism is involved in cellular osmoregulation and antioxidant activity. (D) The riboflavin (vitamin B2) metabolism pathway (hsa00740). Riboflavin is an essential nutrient involved in energy metabolism, antioxidant reactions and other metabolic processes. In the body, riboflavin is enzymatically converted to flavin mononucleotide (FMN) and flavin adenine dinucleotide (FAD), which are crucial cofactors in various redox reactions.

**Figure S6:** Metabolic pathway analysis associated with chronic periodontitis related to blood metabolites. (A) This figure illustrates the vitamin B6 metabolism pathway (hsa00750). Vitamin B6 is an essential cofactor in many metabolic processes, including amino acid metabolism, neurotransmitter synthesis and glucose metabolism. It is converted into pyridoxal‐5′‐phosphate (PLP) in the body, the active form involved in various enzymatic reactions. The nodes represent vitamin B6 and its derivatives, while the edges depict the conversion pathways between these metabolites. (B) The ether lipid metabolism pathway (hsa00565). Ether lipids are a class of lipid molecules containing an ether bond, involved in cellular membrane composition and signalling processes. This pathway includes the biosynthesis and degradation of ether lipids, with multiple enzymatic reactions that play critical roles in various physiological functions. (C) The glycerophospholipid metabolism pathway (hsa00564). Glycerophospholipids are major components of cell membranes, involved in cell signalling, membrane fusion and lipid metabolism. This pathway includes the biosynthesis and degradation of glycerophospholipids, with various key enzymatic reactions that are crucial for maintaining the structure and function of cellular membranes. (D) The caffeine metabolism pathway (hsa00232). Caffeine is a common stimulant that is primarily metabolised in the liver through various enzymatic reactions. This pathway includes the metabolism of caffeine and the formation of its metabolic products (such as theobromine, theophylline and paraxanthine), along with their further breakdown.

**Table S1:** Overview of exposure and outcome data included in this study.
**Table S2:** List of the identification (ID) for each of the 1400 blood metabolites.
**Table S3:** Causal effects of the blood metabolites on chronic periodontitis.
**Table S4:** Sensitivity analyses based on the causal effects of the blood metabolites on chronic periodontitis.
**Table S5:** Basic Information and Classification of Significant Metabolites Identified by Bidirectional MR.
**Table S6:** Causal effects of chronic periodontitis on the blood metabolites.
**Table S7:** Sensitivity analyses based on the causal effects of chronic periodontitis on the blood metabolites.

## Data Availability

All original data were retrieved from two public databases (https://www.ebi.ac.uk/gwas/publications/36635386) (https://r10.finngen.fi/).
