# Supplementary material for: Exploring the Causal Relationship Between Blood Metabolites and Chronic Periodontitis: Insights From Genetic Causal Analysis
Source: J Cell Mol Med. 2025 Oct 31;29(21):e70938. doi: 10.1111/jcmm.70938 (PMC12576583; doi:10.1111/jcmm.70938)
Supplement: Supplementary file 1 — Figure S1: Forest plots for the Mendelian randomisation (MR) of the significant inverse variance weighted (IVW) estimates. (A) The forest plot of 60 positive results from the MR analysis, with blood metabolites sequentially categorised into seven categories. N indicates the number of SNPs used for the analysis of each blood metabolite, OR (95% CI) represents the odds ratio with its 95% confidence interval and p value indicates the level of statistical significance. (B) The forest plot of 60 positive results from the MR analysis, with blood metabolites sequentially categorised into six categories. [file JCMM-29-e70938-s005.docx]

**Supplementary Materials**

**Exploring the causal relationship** **between blood metabolites and chronic periodontitis: Insights from genetic causal analysis**

Weilun Cai^1#^, Huaxuan Zhao^1#^, Panpan Wang^2^，Xiao Chen^1, 3^, Yumeng Yang^3^, Hongle Wu^3^, Zehao Chen^1^, Fuchun Fang^1*^, Wei Qiu^1*^

Corresponding to Wei Qiu and Fuchun Fang, Department of Stomatology, Nanfang Hospital, Southern Medical University, Guangzhou, China. Email: [qiuweiandmj@163.com](mailto:qiuweiandmj@163.com); [fangfuchun@smu.edu.cn](mailto:fangfuchun@smu.edu.cn)

**Contents**

**Supplementary Figures**

Figure S1. Forest plots for the Mendelian randomization (MR) of the significant inverse variance weighted (IVW) estimates.

Figure S2. Scatter plot of significant MR results.

Figure S3. The bar chart and network chart of MSEA associated with blood metabolites related to chronic periodontitis.

Figure S4. The bar chart and network chart of MSEA associated with chronic periodontitis related to blood metabolites.

Figure S5. Metabolic pathway analysis associated with blood metabolites related to chronic periodontitis.

Figure S6. Metabolic pathway analysis associated with chronic periodontitis related to blood metabolites.

**Supplementary Figures and Figure Legends**

**
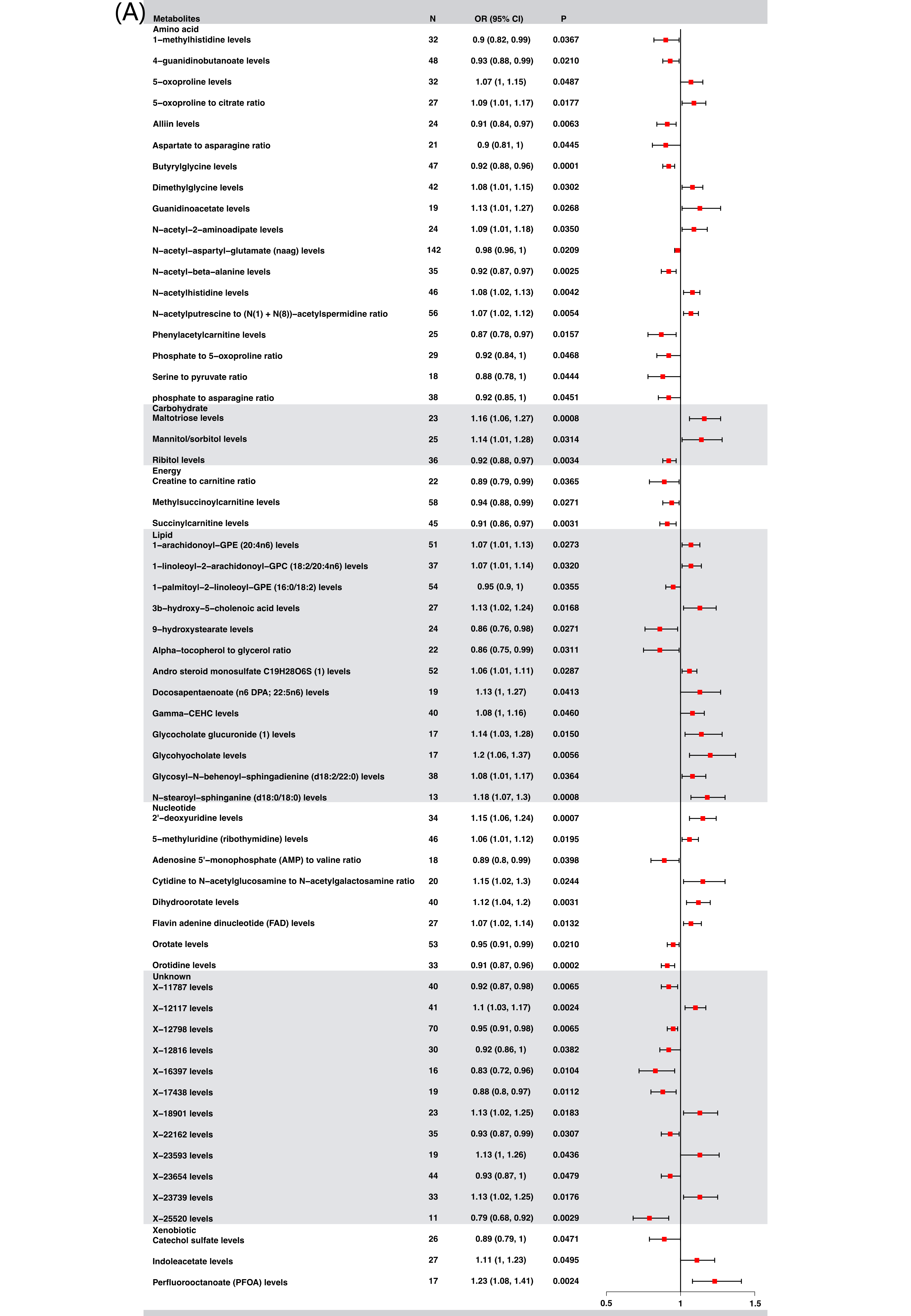
**


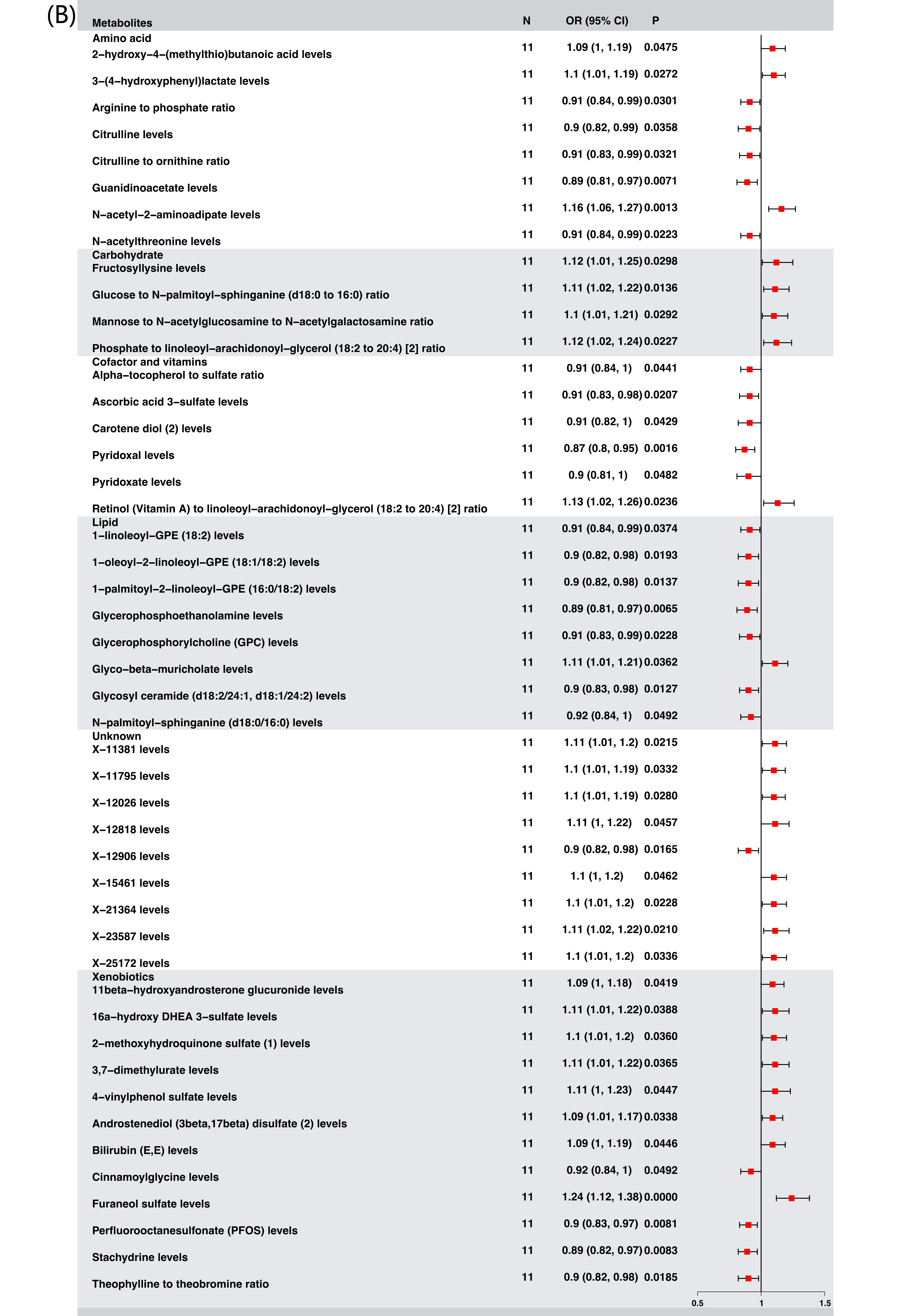


**Figure S1** Forest plots for the Mendelian randomization (MR) of the significant inverse variance weighted (IVW) estimates. (A) The forest plot of 60 positive results from the MR analysis, with blood metabolites sequentially categorized into 7 categories. N indicates the number of SNPs used for the analysis of each blood metabolite, OR (95% CI) represents the odds ratio with its 95% confidence interval, and p-value indicates the level of statistical significance. (B) The forest plot of 60 positive results from the MR analysis, with blood metabolites sequentially categorized into 6 categories.
