# Supplementary material for: Exploring the Causal Relationship Between Blood Metabolites and Chronic Periodontitis: Insights From Genetic Causal Analysis
Source: J Cell Mol Med. 2025 Oct 31;29(21):e70938. doi: 10.1111/jcmm.70938 (PMC12576583; doi:10.1111/jcmm.70938)
Supplement: Supplementary file 2 — Figure S2: Scatter plot of significant MR results. (A) MR analysis scatter plot. (B) Reverse MR analysis scatter plot. (C) Causal effect of butyrylglycine and orotidine on chronic periodontitis. (D) Causal effect of chronic periodontitis on furaneol sulfate. Odds ratio point estimates and 95% confidence intervals of MR results for the causal risk blood metabolites on chronic periodontitis and chronic periodontitis on blood metabolites. [file JCMM-29-e70938-s001.docx]

**
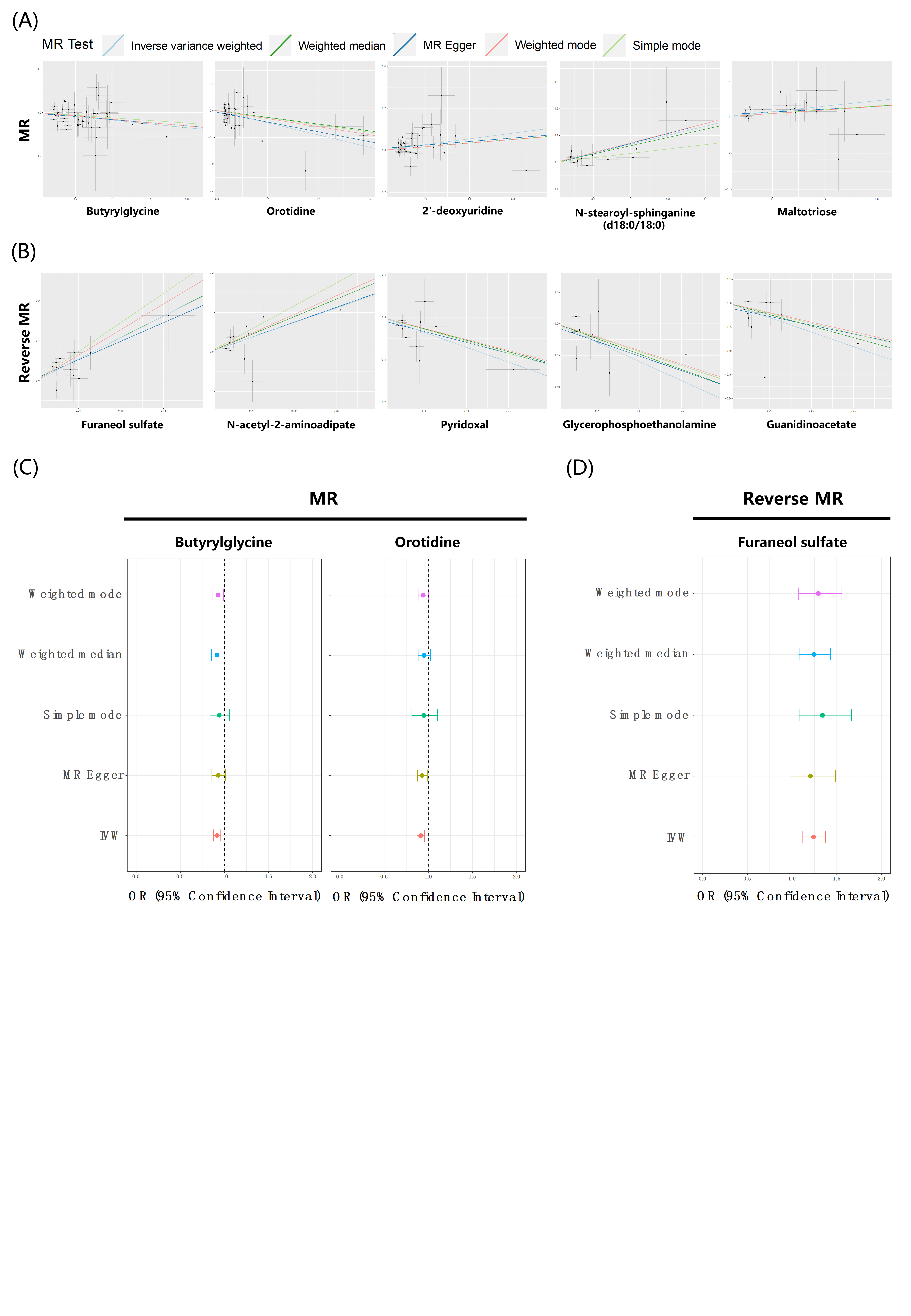
Figure S2** Scatter plot of significant MR results. (A) MR analysis scatter plot. (B) Reverse MR analysis scatter plot. (C) Causal effect of butyrylglycine and orotidine on chronic periodontitis. (D) Causal effect of chronic periodontitis on furaneol sulfate. Odds ratio point estimates and 95% confidence intervals of MR results for the causal risk blood metabolites on chronic periodontitis and chronic periodontitis on blood metabolites.
