# Supplementary material for: Exploring the Causal Relationship Between Blood Metabolites and Chronic Periodontitis: Insights From Genetic Causal Analysis
Source: J Cell Mol Med. 2025 Oct 31;29(21):e70938. doi: 10.1111/jcmm.70938 (PMC12576583; doi:10.1111/jcmm.70938)
Supplement: Supplementary file 3 — Figure S3: The bar chart and network chart of MSEA associated with blood metabolites related to chronic periodontitis. (A) The bar chart. (B) The network chart. Each node represents a metabolite set with its colour based on its p value and its size is based on fold enrichment (hits/expected) to your query. Two metabolite sets are connected by an edge if the number of their shared metabolites is over 25% of the total number of their combined metabolite sets. [file JCMM-29-e70938-s003.docx]

**
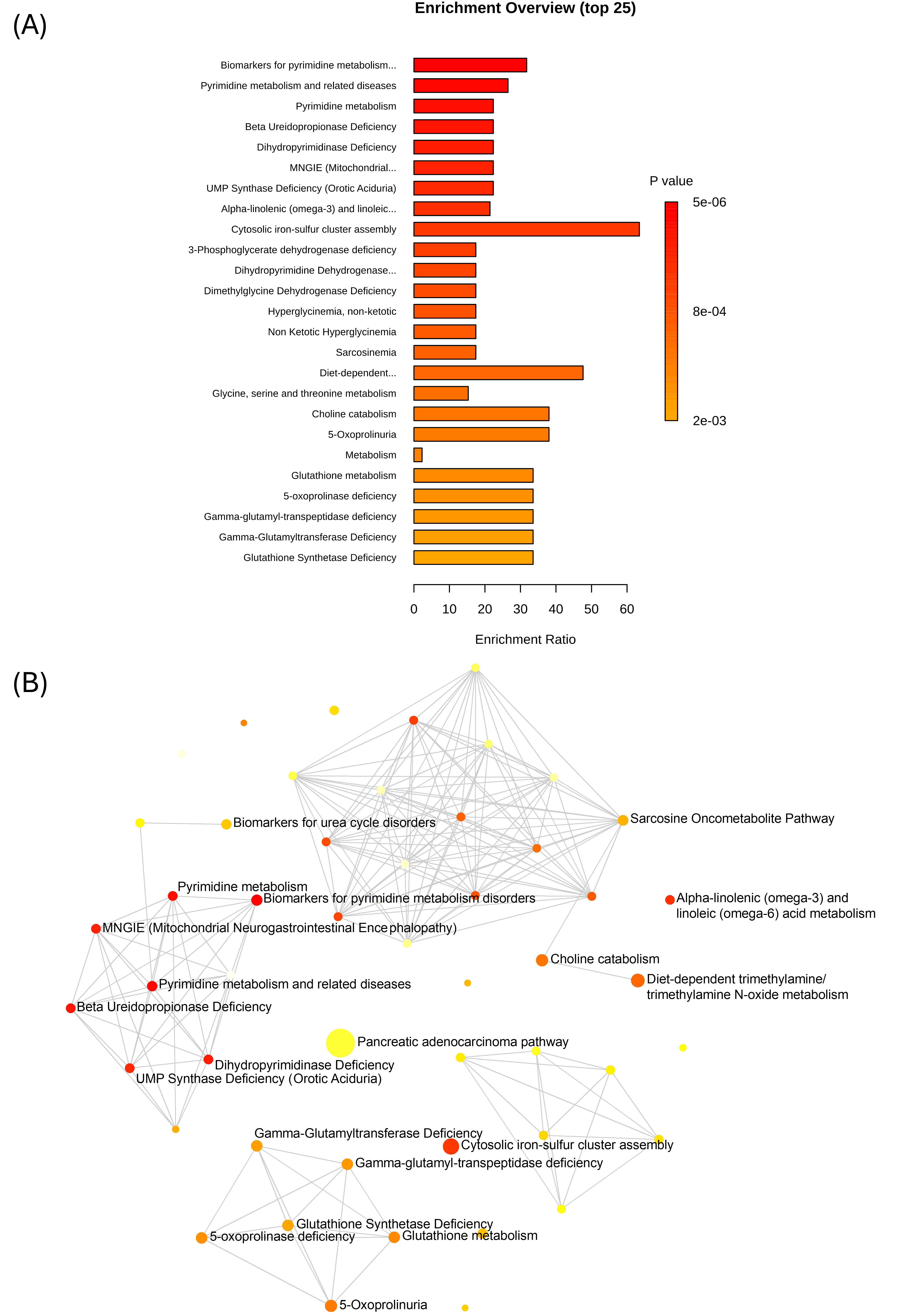
Figure S3** The bar chart and network chart of MSEA associated with blood metabolites related to chronic periodontitis. (A) The bar chart. (B) The network chart. Each node represents a metabolite set with its color based on its p value, and its size is based on fold enrichment (hits/expected) to your query. Two metabolite sets are connected by an edge if the number of their shared metabolites is over 25% of the total number of their combined metabolite sets.
