# Supplementary material for: Exploring the Causal Relationship Between Blood Metabolites and Chronic Periodontitis: Insights From Genetic Causal Analysis
Source: J Cell Mol Med. 2025 Oct 31;29(21):e70938. doi: 10.1111/jcmm.70938 (PMC12576583; doi:10.1111/jcmm.70938)
Supplement: Supplementary file 5 — Figure S5: Metabolic pathway analysis associated with blood metabolites related to chronic periodontitis. (A)This figure illustrates the pyrimidine metabolism pathway (hsa00240), which involves de novo synthesis, degradation and nucleotide metabolism. This pathway is crucial for the synthesis of DNA and RNA in cells. Metabolites are converted through various enzyme‐catalysed reactions, eventually participating in the synthesis or degradation of nucleotides. Different nodes in the figure represent distinct metabolites in the pyrimidine metabolism, while the connections depict the relationships and conversions between these metabolites. (B)The glycine, serine and threonine metabolism pathway (hsa00260). This pathway involves the interconversion of glycine and serine, as well as the metabolism of threonine, an essential amino acid. These amino acids play crucial roles in the synthesis of nucleic acids and proteins, as well as in various metabolic pathways. (C) The arginine and proline metabolism pathway (hsa00330). This pathway involves the synthesis and degradation of arginine and proline, which play crucial roles in protein synthesis, the urea cycle and various metabolic processes. Arginine metabolism is also linked to nitric oxide production, while proline metabolism is involved in cellular osmoregulation and antioxidant activity. (D) The riboflavin (vitamin B2) metabolism pathway (hsa00740). Riboflavin is an essential nutrient involved in energy metabolism, antioxidant reactions and other metabolic processes. In the body, riboflavin is enzymatically converted to flavin mononucleotide (FMN) and flavin adenine dinucleotide (FAD), which are crucial cofactors in various redox reactions. [file JCMM-29-e70938-s004.docx]

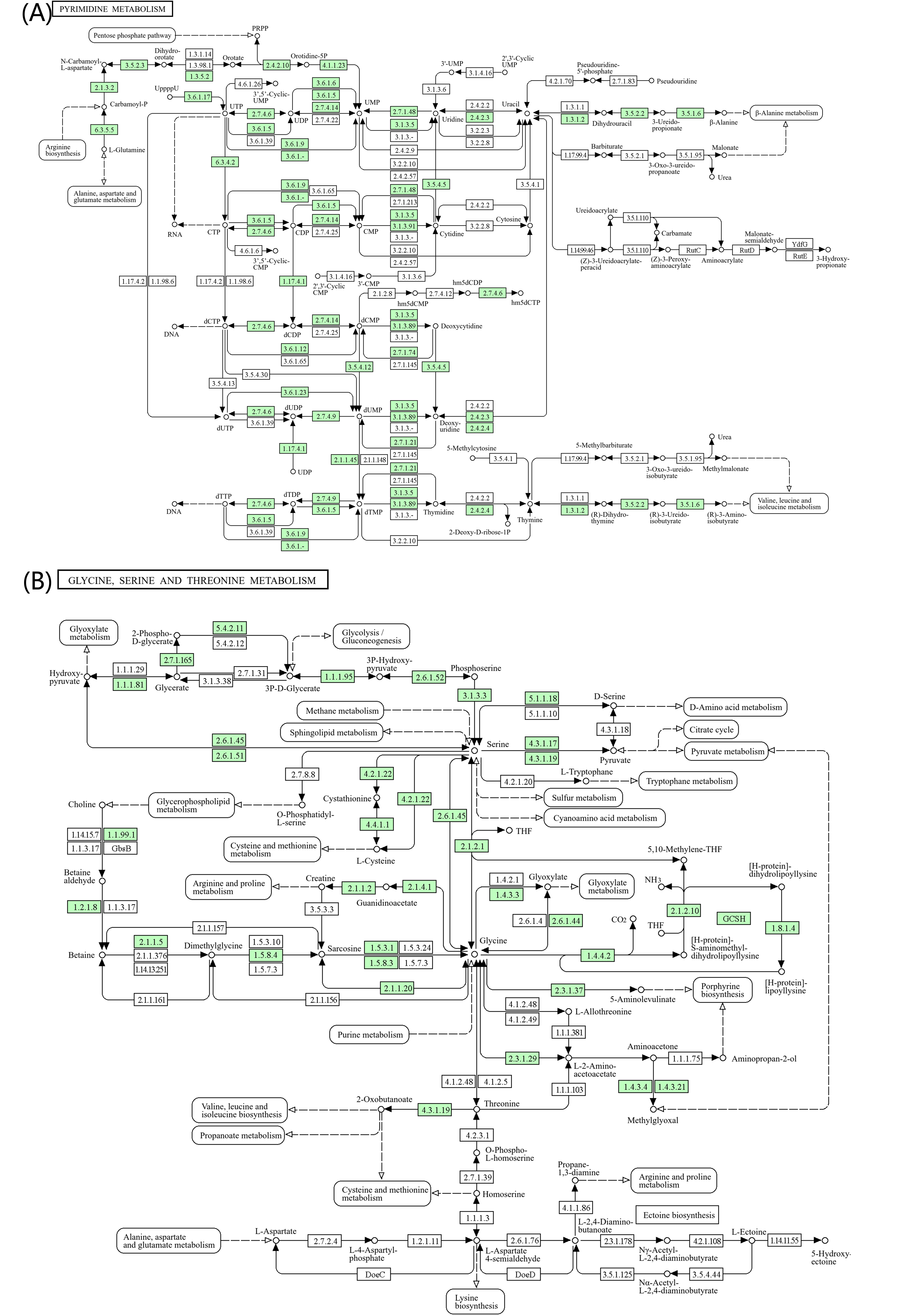


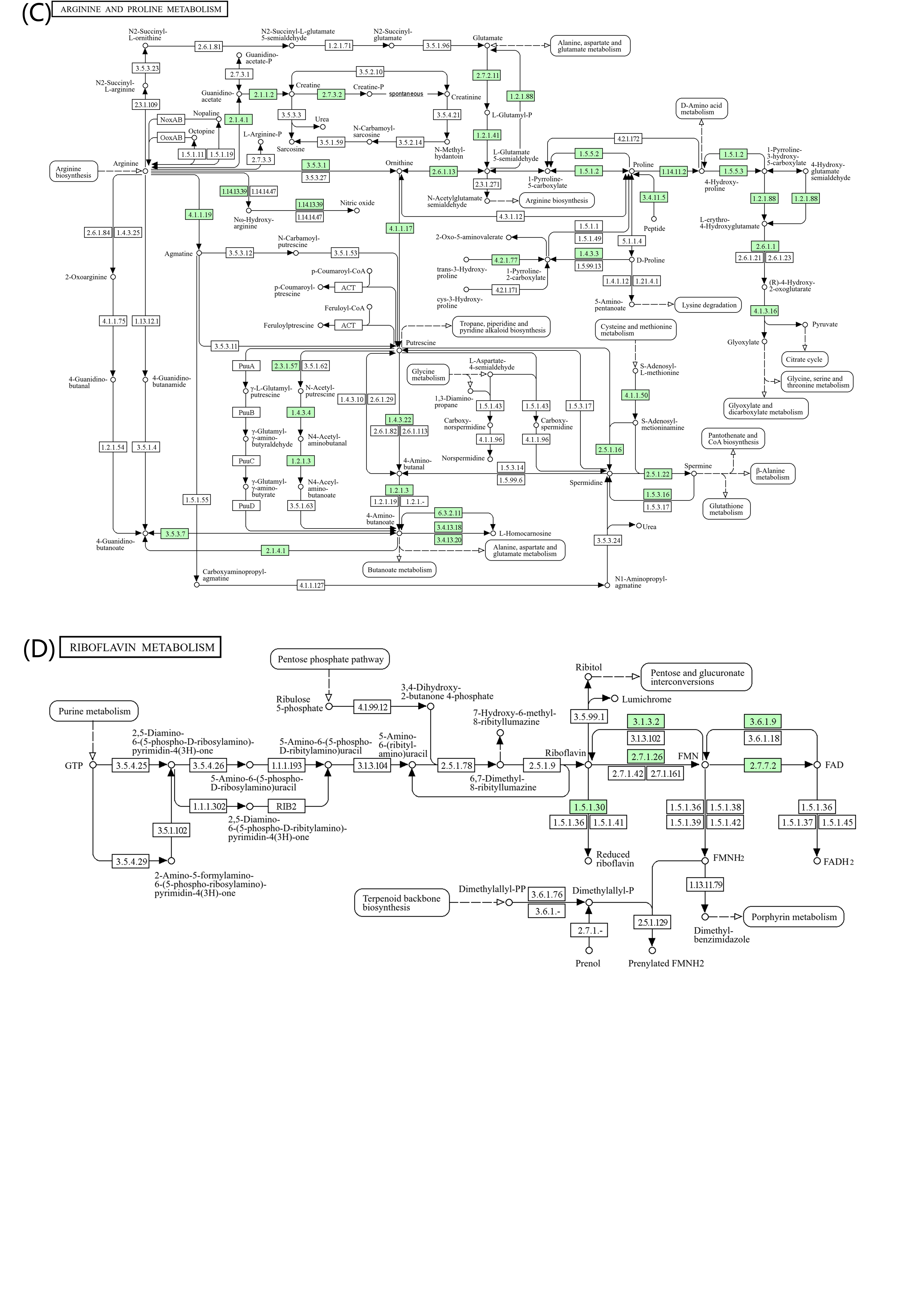


**Figure S5** Metabolic pathway analysis associated with blood metabolites related to chronic periodontitis. (A)This figure illustrates the pyrimidine metabolism pathway (hsa00240), which involves de novo synthesis, degradation, and nucleotide metabolism. This pathway is crucial for the synthesis of DNA and RNA in cells. Metabolites are converted through various enzyme-catalyzed reactions, eventually participating in the synthesis or degradation of nucleotides. Different nodes in the figure represent distinct metabolites in the pyrimidine metabolism, while the connections depict the relationships and conversions between these metabolites. (B)The glycine, serine, and threonine metabolism pathway (hsa00260). This pathway involves the interconversion of glycine and serine, as well as the metabolism of threonine, an essential amino acid. These amino acids play crucial roles in the synthesis of nucleic acids and proteins, as well as in various metabolic pathways. (C) The arginine and proline metabolism pathway (hsa00330). This pathway involves the synthesis and degradation of arginine and proline, which play crucial roles in protein synthesis, the urea cycle, and various metabolic processes. Arginine metabolism is also linked to nitric oxide production, while proline metabolism is involved in cellular osmoregulation and antioxidant activity. (D) The riboflavin (vitamin B2) metabolism pathway (hsa00740). Riboflavin is an essential nutrient involved in energy metabolism, antioxidant reactions, and other metabolic processes. In the body, riboflavin is enzymatically converted to flavin mononucleotide (FMN) and flavin adenine dinucleotide (FAD), which are crucial cofactors in various redox reactions.
