# Supplementary material for: Exploring the Causal Relationship Between Blood Metabolites and Chronic Periodontitis: Insights From Genetic Causal Analysis
Source: J Cell Mol Med. 2025 Oct 31;29(21):e70938. doi: 10.1111/jcmm.70938 (PMC12576583; doi:10.1111/jcmm.70938)
Supplement: Supplementary file 6 — Figure S6: Metabolic pathway analysis associated with chronic periodontitis related to blood metabolites. (A) This figure illustrates the vitamin B6 metabolism pathway (hsa00750). Vitamin B6 is an essential cofactor in many metabolic processes, including amino acid metabolism, neurotransmitter synthesis and glucose metabolism. It is converted into pyridoxal‐5′‐phosphate (PLP) in the body, the active form involved in various enzymatic reactions. The nodes represent vitamin B6 and its derivatives, while the edges depict the conversion pathways between these metabolites. (B) The ether lipid metabolism pathway (hsa00565). Ether lipids are a class of lipid molecules containing an ether bond, involved in cellular membrane composition and signalling processes. This pathway includes the biosynthesis and degradation of ether lipids, with multiple enzymatic reactions that play critical roles in various physiological functions. (C) The glycerophospholipid metabolism pathway (hsa00564). Glycerophospholipids are major components of cell membranes, involved in cell signalling, membrane fusion and lipid metabolism. This pathway includes the biosynthesis and degradation of glycerophospholipids, with various key enzymatic reactions that are crucial for maintaining the structure and function of cellular membranes. (D) The caffeine metabolism pathway (hsa00232). Caffeine is a common stimulant that is primarily metabolised in the liver through various enzymatic reactions. This pathway includes the metabolism of caffeine and the formation of its metabolic products (such as theobromine, theophylline and paraxanthine), along with their further breakdown. [file JCMM-29-e70938-s002.docx]

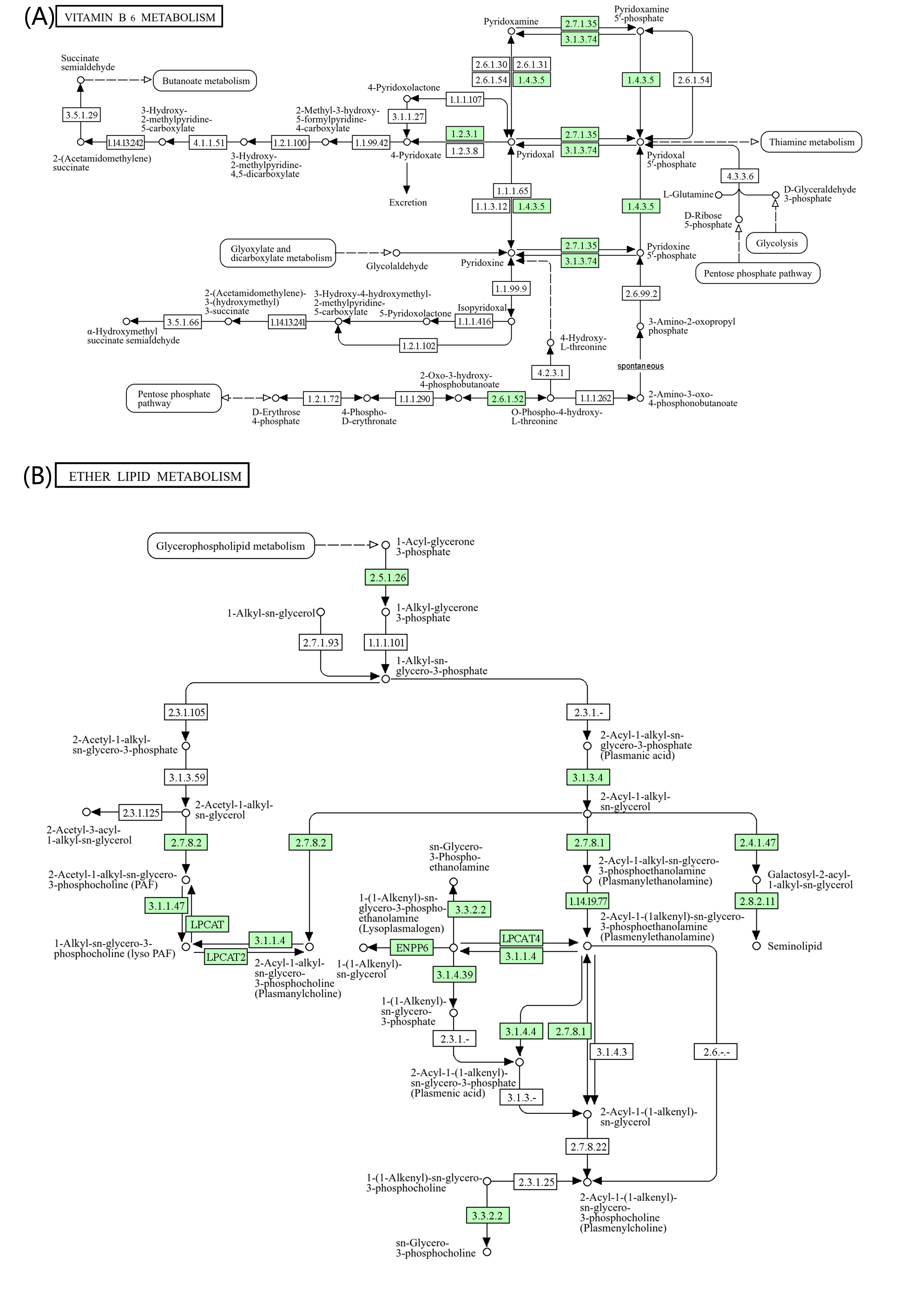


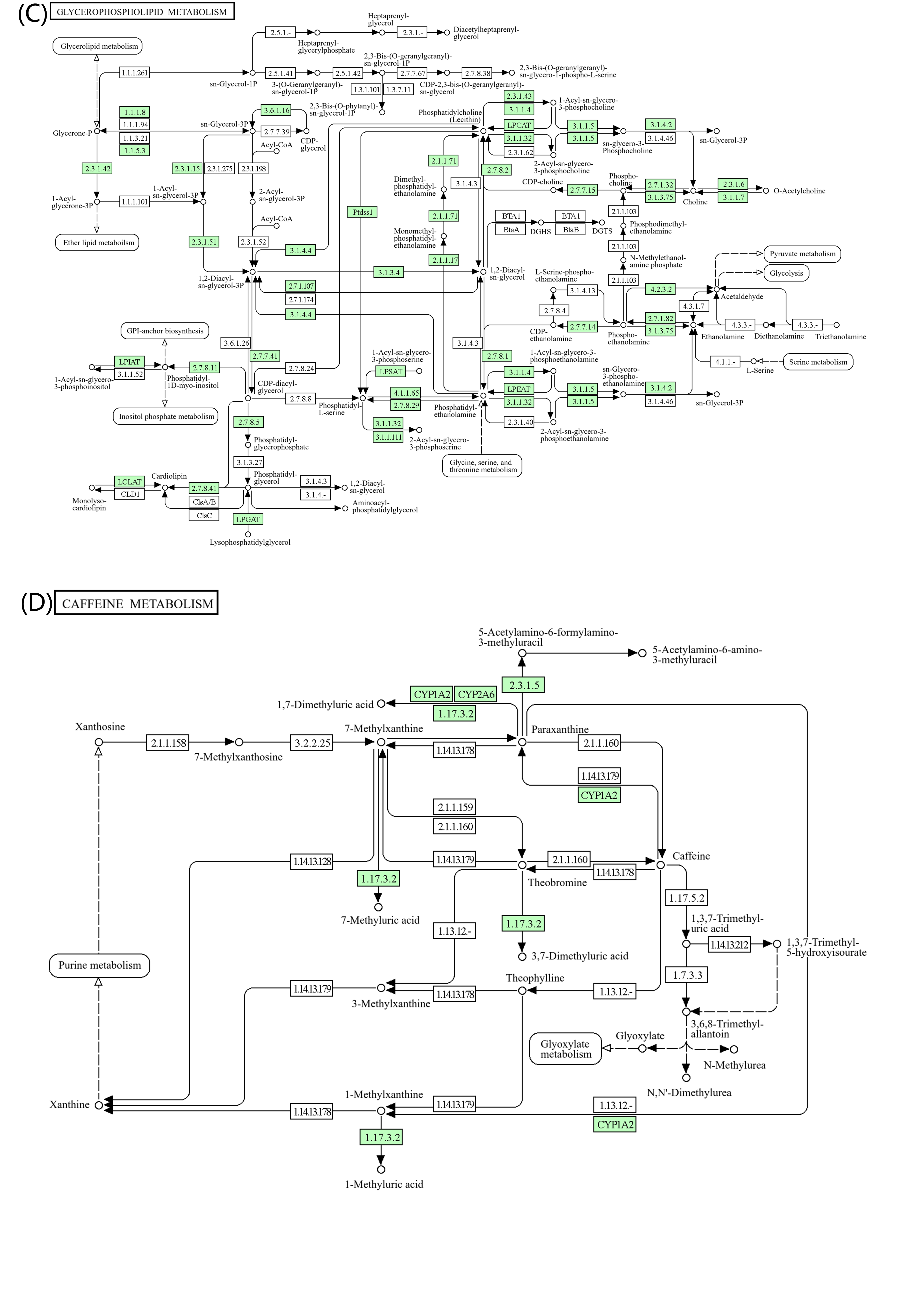


**Figure S6** Metabolic pathway analysis associated with chronic periodontitis related to blood metabolites. (A) This figure illustrates the vitamin B6 metabolism pathway (hsa00750). Vitamin B6 is an essential cofactor in many metabolic processes, including amino acid metabolism, neurotransmitter synthesis, and glucose metabolism. It is converted into pyridoxal-5’-phosphate (PLP) in the body, the active form involved in various enzymatic reactions. The nodes represent vitamin B6 and its derivatives, while the edges depict the conversion pathways between these metabolites. (B) The ether lipid metabolism pathway (hsa00565). Ether lipids are a class of lipid molecules containing an ether bond, involved in cellular membrane composition and signaling processes. This pathway includes the biosynthesis and degradation of ether lipids, with multiple enzymatic reactions that play critical roles in various physiological functions. (C) The glycerophospholipid metabolism pathway (hsa00564). Glycerophospholipids are major components of cell membranes, involved in cell signaling, membrane fusion, and lipid metabolism. This pathway includes the biosynthesis and degradation of glycerophospholipids, with various key enzymatic reactions that are crucial for maintaining the structure and function of cellular membranes. (D) The caffeine metabolism pathway (hsa00232). Caffeine is a common stimulant that is primarily metabolized in the liver through various enzymatic reactions. This pathway includes the metabolism of caffeine and the formation of its metabolic products (such as theobromine, theophylline, and paraxanthine), along with their further breakdown.
