# Supplementary material for: Exploring the Causal Relationship Between Blood Metabolites and Chronic Periodontitis: Insights From Genetic Causal Analysis
Source: J Cell Mol Med. 2025 Oct 31;29(21):e70938. doi: 10.1111/jcmm.70938 (PMC12576583; doi:10.1111/jcmm.70938)
Supplement: Supplementary file 7 — Table S1: Overview of exposure and outcome data included in this study. Table S2: List of the identification (ID) for each of the 1400 blood metabolites. Table S3: Causal effects of the blood metabolites on chronic periodontitis. Table S4: Sensitivity analyses based on the causal effects of the blood metabolites on chronic periodontitis. Table S5: Basic Information and Classification of Significant Metabolites Identified by Bidirectional MR. Table S6: Causal effects of chronic periodontitis on the blood metabolites. Table S7: Sensitivity analyses based on the causal effects of chronic periodontitis on the blood metabolites. [file JCMM-29-e70938-s007.docx]

**Supplementary Materials**

**Exploring the causal relationship** **between blood metabolites and chronic periodontitis: Insights from genetic causal analysis**

Weilun Cai^1#^, Huaxuan Zhao^1#^, Panpan Wang^2^，Xiao Chen^1, 3^, Yumeng Yang^3^, Hongle Wu^3^, Zehao Chen^1^, Fuchun Fang^1*^, Wei Qiu^1*^

Corresponding to Wei Qiu and Fuchun Fang, Department of Stomatology, Nanfang Hospital, Southern Medical University, Guangzhou, China. Email: [qiuweiandmj@163.com](mailto:qiuweiandmj@163.com); [fangfuchun@smu.edu.cn](mailto:fangfuchun@smu.edu.cn)

**Contents**

**Supplementary Tables**

Table S1. Overview of exposure and outcome data included in this study.

Table S2. List of the identification (ID) for each of the 1400 blood metabolites.

Table S3. Causal effects of the blood metabolites on chronic periodontitis.

Table S4. Sensitivity analyses based on the causal effects of the blood metabolites on chronic periodontitis.

Table S5. Basic Information and Classification of Significant Metabolites Identified by Bidirectional MR.

Table S6. Causal effects of chronic periodontitis on the blood metabolites.

Table S7. Sensitivity analyses based on the causal effects of chronic periodontitis on the blood metabolites.

**Table S1.** Overview of exposure and outcome data included in this study.

| **PMID** | **Exposure or outcome** | **ID** | **Website** | **Download** | **Number of cases** | **Number of controls** | **Consortium** | **Set Parameters** |
| --- | --- | --- | --- | --- | --- | --- | --- | --- |
| - | Chronic periodontitis | K11_PERIODON_CHRON | https://risteys.finregistry.fi/endpoints/K11_PERIODON_CHRON | https://storage.googleapis.com/finngen-public-data-r10/summary_stats/finngen_R10_K11_PERIODON_CHRON.gz | 4784 individuals | 272,252 controls | Finngen | - |
| 36635386 | Human Blood Metabolites | GCST90199621 to GCST90201020 | https://www.ebi.ac.uk/gwas/publications/36635386 | - | - | - | - | P < 1E05,  r^2^：0.1,  LD：500kb |
| The MR analysis was conducted using R (version 4.3.1) along with the R package "Two Sample MR" (version 0.5.8) | | | | | | | | |

**Table S2.** List of the identification (ID) for each of the 1400 blood metabolites

| **ID** | **Metabolites** | **DiscoverySampleAncestry** |
| --- | --- | --- |
| GCST90199621 | Carnitine levels | 8192 European |
| GCST90199622 | Benzoate levels | 7306 European |
| GCST90199623 | Maltotriose levels | 4641 European |
| GCST90199624 | Hippurate levels | 8260 European |
| GCST90199625 | Methionine sulfoxide levels | 8243 European |
| GCST90199626 | Xanthurenate levels | 7965 European |
| GCST90199627 | N-alpha-acetylornithine levels | 5606 European |
| GCST90199628 | 3-methylhistidine levels | 8289 European |
| GCST90199629 | Glycerophosphorylcholine (GPC) levels | 8212 European |
| GCST90199630 | Phenylacetate levels | 7059 European |
| GCST90199631 | 3-methyl-2-oxovalerate levels | 8257 European |
| GCST90199632 | Tartarate levels | 7099 European |
| GCST90199633 | Suberate (C8-DC) levels | 6902 European |
| GCST90199634 | 5-hydroxylysine levels | 8128 European |
| GCST90199635 | 3-phenylpropionate hydrocinnamate levels | 7925 European |
| GCST90199636 | Kynurenine levels | 8244 European |
| GCST90199637 | N-acetylglutamate levels | 7802 European |
| GCST90199638 | Glycerol 3-phosphate levels | 8201 European |
| GCST90199639 | Imidazole lactate levels | 8256 European |
| GCST90199640 | 4-guanidinobutanoate levels | 8144 European |
| GCST90199641 | Glucuronate levels | 7988 European |
| GCST90199642 | Ribitol levels | 8128 European |
| GCST90199643 | Glycodeoxycholate levels | 6877 European |
| GCST90199644 | Theobromine levels | 8137 European |
| GCST90199645 | Quinate levels | 8200 European |
| GCST90199646 | Gentisate levels | 8013 European |
| GCST90199647 | Theophylline levels | 7822 European |
| GCST90199648 | 1,5-anhydroglucitol (1,5-ag) levels | 8139 European |
| GCST90199649 | 1-stearoyl-2-arachidonoyl-GPI (18:0/20:4) levels | 8134 European |
| GCST90199650 | Indolelactate levels | 8233 European |
| GCST90199651 | Docosahexaenoate DHA; 22:6n3 levels | 8279 European |
| GCST90199652 | 1-stearoyl-2-oleoyl-GPS (18:0/18:1) levels | 8055 European |
| GCST90199653 | 1-myristoyl-2-palmitoyl-gpc (14:0/16:0) levels | 8271 European |
| GCST90199654 | Paraxanthine levels in elite athletes | 7840 European |
| GCST90199655 | 4-methyl-2-oxopentanoate levels | 8255 European |
| GCST90199656 | 3-indoxyl sulfate levels | 8242 European |
| GCST90199657 | Sphingosine 1-phosphate levels | 8246 European |
| GCST90199658 | Alpha-hydroxyisocaproate levels | 8250 European |
| GCST90199659 | 1-stearoyl-GPI (18:0) levels | 8260 European |
| GCST90199660 | 1,2-dipalmitoyl-gpc (16:0/16:0) levels | 8264 European |
| GCST90199661 | Maleate levels | 8171 European |
| GCST90199662 | Isovalerate (i5:0) levels | 7862 European |
| GCST90199663 | 4-acetylphenol sulfate levels | 7135 European |
| GCST90199664 | Phenyllactate (PLA) levels in elite athletes | 8235 European |
| GCST90199665 | N-acetylaspartate (naa) levels | 8148 European |
| GCST90199666 | Palmitoylcarnitine levels (Metabolon platform) | 8256 European |
| GCST90199667 | Erythritol levels in elite athletes | 8167 European |
| GCST90199668 | Hexanoylcarnitine levels (Biocrates platform) | 8242 European |
| GCST90199669 | Acetylcarnitine levels (Biocrates platform) | 8262 European |
| GCST90199670 | Iminodiacetate (IDA) levels | 8213 European |
| GCST90199671 | Cysteine s-sulfate levels | 8229 European |
| GCST90199672 | Oxalate (ethanedioate) levels | 8181 European |
| GCST90199673 | DHEAS levels | 8228 European |
| GCST90199674 | 3-hydroxymyristate levels | 8235 European |
| GCST90199675 | Homoarginine levels | 8265 European |
| GCST90199676 | 1-oleoylglycerol (18:1) levels | 8225 European |
| GCST90199677 | 3-methyl-2-oxobutyrate levels | 8254 European |
| GCST90199678 | Tartronate (hydroxymalonate) levels | 8125 European |
| GCST90199679 | 2,3-dihydroxypyridine levels | 6496 European |
| GCST90199680 | 3-hydroxyoctanoate levels | 8182 European |
| GCST90199681 | 2-hydroxyoctanoate levels | 8224 European |
| GCST90199682 | Homocitrulline levels | 7998 European |
| GCST90199683 | Indoleacetate levels | 8208 European |
| GCST90199684 | 1-linoleoylglycerol (18:2) levels | 8212 European |
| GCST90199685 | 2-linoleoylglycerol (18:2) levels | 6378 European |
| GCST90199686 | Indolepropionate levels | 8153 European |
| GCST90199687 | Threonate levels | 8151 European |
| GCST90199688 | N-acetylglycine levels | 8250 European |
| GCST90199689 | Glycolithocholate levels | 7158 European |
| GCST90199690 | EDTA levels | 8239 European |
| GCST90199691 | 1-methylhistidine levels | 8226 European |
| GCST90199692 | Citramalate levels | 7125 European |
| GCST90199693 | 3-hydroxydecanoate levels | 8239 European |
| GCST90199694 | 1,3-dimethylurate levels | 7444 European |
| GCST90199695 | Galactonate levels | 7518 European |
| GCST90199696 | Trigonelline levels | 8272 European |
| GCST90199697 | Dodecanedioate levels | 8240 European |
| GCST90199698 | Isobutyrylcarnitine (c4) levels | 8243 European |
| GCST90199699 | 3-methylxanthine levels | 8085 European |
| GCST90199700 | 3-hydroxylaurate levels | 8206 European |
| GCST90199701 | Pyridoxate levels | 8089 European |
| GCST90199702 | 5-hydroxyhexanoate levels | 7097 European |
| GCST90199703 | Gamma-glutamylvaline levels | 8179 European |
| GCST90199704 | 3-hydroxysebacate levels | 7325 European |
| GCST90199705 | Propionylglycine levels | 7311 European |
| GCST90199706 | Butyrylglycine levels | 6044 European |
| GCST90199707 | Pro-hydroxy-pro levels | 8208 European |
| GCST90199708 | Propionylcarnitine (c3) levels | 8273 European |
| GCST90199709 | Adrenate (22:4n6) levels | 8279 European |
| GCST90199710 | Docosatrienoate (22:3n3) levels | 6511 European |
| GCST90199711 | Docosadienoate (22:2n6) levels | 8264 European |
| GCST90199712 | 3-carboxy-4-methyl-5-propyl-2-furanpropanoate (cmpf) levels | 8270 European |
| GCST90199713 | Docosapentaenoate n3 DPA; 22:5n3 levels | 8284 European |
| GCST90199714 | 3-hydroxy-2-ethylpropionate levels | 8238 European |
| GCST90199715 | Myristoleate (14:1n5) levels | 8255 European |
| GCST90199716 | 10-undecenoate (11:1n1) levels | 8243 European |
| GCST90199717 | 1-methyl-4-imidazoleacetate levels | 8090 European |
| GCST90199718 | Sebacate (C10-DC) levels | 7926 European |
| GCST90199719 | Tauro-beta-muricholate levels | 4661 European |
| GCST90199720 | Octanoylcarnitine (c8) levels | 8226 European |
| GCST90199721 | Stearidonate (18:4n3) levels | 8240 European |
| GCST90199722 | Decanoylcarnitine (C10) levels | 8225 European |
| GCST90199723 | 5-dodecenoate (12:1n7) levels | 8248 European |
| GCST90199724 | N-acetylisoleucine levels | 7223 European |
| GCST90199725 | Campesterol levels | 5300 European |
| GCST90199726 | N-acetylthreonine levels | 8223 European |
| GCST90199727 | 1-oleoyl-GPC (18:1) levels | 8257 European |
| GCST90199728 | 10-nonadecenoate (19:1n9) levels | 8277 European |
| GCST90199729 | Piperine levels | 8155 European |
| GCST90199730 | Carnitine C14 levels | 8241 European |
| GCST90199731 | 10-heptadecenoate (17:1n7) levels | 8282 European |
| GCST90199732 | 1-stearoyl-gpc (18:0) levels | 8240 European |
| GCST90199733 | 1-palmitoyl-GPC (16:0) levels | 8232 European |
| GCST90199734 | Hyocholate levels | 5782 European |
| GCST90199735 | N-acetylhistidine levels | 7054 European |
| GCST90199736 | Alpha-hydroxyisovalerate levels | 8208 European |
| GCST90199737 | N-acetylproline levels | 7100 European |
| GCST90199738 | Stachydrine levels | 8297 European |
| GCST90199739 | Salicyluric glucuronide levels | 5190 European |
| GCST90199740 | P-cresol sulfate levels | 8183 European |
| GCST90199741 | Gamma-glutamylglycine levels | 8263 European |
| GCST90199742 | 1-linoleoyl-gpc (18:2) levels | 8262 European |
| GCST90199743 | Stearoylcarnitine levels | 8133 European |
| GCST90199744 | Eicosenoate (20:1) levels | 8277 European |
| GCST90199745 | Aconitate [cis or trans] levels | 8262 European |
| GCST90199746 | Erythronate levels | 8198 European |
| GCST90199747 | Isovalerylcarnitine (C5) levels | 8266 European |
| GCST90199748 | Linolenate [alpha or gamma; (18:3n3 or 6)] levels | 8257 European |
| GCST90199749 | Gamma-glutamylthreonine levels | 8231 European |
| GCST90199750 | Laurylcarnitine levels | 8241 European |
| GCST90199751 | Gamma-glutamylmethionine levels | 8237 European |
| GCST90199752 | Epiandrosterone sulfate levels | 8174 European |
| GCST90199753 | Gamma-glutamyltryptophan levels | 7902 European |
| GCST90199754 | 7-methylxanthine levels | 8019 European |
| GCST90199755 | 3,7-dimethylurate levels | 6894 European |
| GCST90199756 | 1,7-dimethyluric acid levels | 7950 European |
| GCST90199757 | 1,3,7-trimethylurate levels | 7446 European |
| GCST90199758 | N4-acetylcytidine levels | 7425 European |
| GCST90199759 | Indolebutyrate levels | 4423 European |
| GCST90199760 | 5-acetylamino-6-amino-3-methyluracil levels | 8006 European |
| GCST90199761 | N1-methylinosine levels | 8147 European |
| GCST90199762 | N6-carbamoylthreonyladenosine levels | 8176 European |
| GCST90199763 | 1-methylxanthine levels | 7959 European |
| GCST90199764 | N2,n2-dimethylguanosine levels | 8210 European |
| GCST90199765 | 4-hydroxyhippurate levels | 8259 European |
| GCST90199766 | 3-(3-amino-3-carboxypropyl)uridine levels | 7679 European |
| GCST90199767 | 5,6-dihydrouridine levels | 8210 European |
| GCST90199768 | Phenylacetylglutamine levels | 8251 European |
| GCST90199769 | 3-hydroxydodecanedioate levels | 7168 European |
| GCST90199770 | 1-arachidonylglycerol (20:4) levels | 7650 European |
| GCST90199771 | 7-methylguanine levels | 8239 European |
| GCST90199772 | 1-stearoyl-GPE (18:0) levels | 8270 European |
| GCST90199773 | Phenol sulfate levels | 8249 European |
| GCST90199774 | Oleoylcarnitine levels | 8260 European |
| GCST90199775 | Gamma-glutamylisoleucine levels | 8267 European |
| GCST90199776 | Malonylcarnitine levels | 5822 European |
| GCST90199777 | 1-palmitoleoyl-GPC (16:1) levels | 8227 European |
| GCST90199778 | N-methyl-2-pyridone-5-carboxamide levels | 8177 European |
| GCST90199779 | Gamma-glutamyl-2-aminobutyrate levels | 7826 European |
| GCST90199780 | 1-stearoyl-GPG (18:0) levels | 6806 European |
| GCST90199781 | Hexanoylglycine levels | 6015 European |
| GCST90199782 | Glutamine degradant levels | 8293 European |
| GCST90199783 | 5-methyluridine (ribothymidine) levels | 8168 European |
| GCST90199784 | Cysteine-glutathione disulfide levels | 7812 European |
| GCST90199785 | Isovalerylglycine levels | 7100 European |
| GCST90199786 | 2-hydroxy-3-methylvalerate levels | 8246 European |
| GCST90199787 | Homostachydrine levels | 8248 European |
| GCST90199788 | 1-arachidonoyl-gpc (20:4n6) levels | 8267 European |
| GCST90199789 | 2-palmitoyl-GPC (16:0) levels | 8249 European |
| GCST90199790 | 2-palmitoleoyl-GPC (16:1) levels | 6697 European |
| GCST90199791 | 1-arachidonoyl-GPE (20:4n6) levels | 8270 European |
| GCST90199792 | 1-palmitoyl-GPE (16:0) levels | 8276 European |
| GCST90199793 | 1-linoleoyl-GPE (18:2) levels | 8279 European |
| GCST90199794 | 1-oleoyl-GPE (18:1) levels | 8283 European |
| GCST90199795 | 2-hydroxypalmitate levels | 8247 European |
| GCST90199796 | Docosapentaenoate (n6 DPA; 22:5n6) levels | 7540 European |
| GCST90199797 | Gulonate levels | 7731 European |
| GCST90199798 | Isobutyrylglycine levels | 7465 European |
| GCST90199799 | Glutarylcarnitine (c5-dc) levels | 8238 European |
| GCST90199800 | Carnitine C5:1 levels | 8097 European |
| GCST90199801 | Hydroquinone sulfate levels | 7682 European |
| GCST90199802 | Beta-hydroxyisovaleroylcarnitine levels | 8232 European |
| GCST90199803 | Catechol sulfate levels | 8242 European |
| GCST90199804 | 7-alpha-hydroxy-3-oxo-4-cholestenoate (7-hoca) levels | 8236 European |
| GCST90199805 | Octadecanedioate levels | 8223 European |
| GCST90199806 | Tetradecanedioate (C14-DC) levels | 8268 European |
| GCST90199807 | Hexadecanedioate (C16-DC) levels | 8260 European |
| GCST90199808 | Glycerophosphoethanolamine levels | 8231 European |
| GCST90199809 | Taurolithocholate 3-sulfate levels | 8124 European |
| GCST90199810 | 1-palmitoyl-GPI (16:0) levels | 8246 European |
| GCST90199811 | Glycolithocholate sulfate levels | 8165 European |
| GCST90199812 | 3-(3-hydroxyphenyl)propionate levels | 7304 European |
| GCST90199813 | Deoxycarnitine levels | 8271 European |
| GCST90199814 | Alpha-hydroxycaproate levels | 5169 European |
| GCST90199815 | 1-ribosyl-imidazoleacetate levels | 8205 European |
| GCST90199816 | 3,4-dihydroxybutyrate levels | 8142 European |
| GCST90199817 | Indoleacetylglutamine levels | 6161 European |
| GCST90199818 | Mannitol/sorbitol levels | 8211 European |
| GCST90199819 | Tryptophan betaine levels | 8216 European |
| GCST90199820 | Dihomo-linolenate (20:3n3 or n6) levels | 8273 European |
| GCST90199821 | 1-linoleoyl-GPI (18:2) levels | 8268 European |
| GCST90199822 | 1-oleoyl-GPI (18:1) levels | 8252 European |
| GCST90199823 | O-cresol sulfate levels | 7315 European |
| GCST90199824 | 4-vinylphenol sulfate levels | 8280 European |
| GCST90199825 | Pyrraline levels | 7920 European |
| GCST90199826 | N6-acetyllysine levels | 8242 European |
| GCST90199827 | Thymol sulfate levels | 8063 European |
| GCST90199828 | N-acetylserine levels | 8217 European |
| GCST90199829 | 3-methyladipate levels | 6958 European |
| GCST90199830 | 4-ethylphenylsulfate levels | 8139 European |
| GCST90199831 | 1-stearoyl-2-oleoyl-GPE (18:0/18:1) levels | 8273 European |
| GCST90199832 | Dimethylarginine (sdma + adma) levels | 8233 European |
| GCST90199833 | Hexanoylglutamine levels | 8128 European |
| GCST90199834 | Gamma-glutamylalanine levels | 7518 European |
| GCST90199835 | 5alpha-androstan-3beta,17beta-diol disulfate levels | 8199 European |
| GCST90199836 | N-methylproline levels | 8299 European |
| GCST90199837 | Carotenoid levels (cryptoxanthin) | 8226 European |
| GCST90199838 | 5alpha-pregnan-3beta,20alpha-diol disulfate levels | 8130 European |
| GCST90199839 | Taurocholenate sulfate levels | 8249 European |
| GCST90199840 | Pregnenediol disulfate (C21H34O8S2) levels | 8245 European |
| GCST90199841 | Glycocholenate sulfate levels | 8250 European |
| GCST90199842 | Androstenediol (3beta,17beta) disulfate (1) levels | 8222 European |
| GCST90199843 | Chiro-inositol levels | 4429 European |
| GCST90199844 | 1-palmitoyl-2-linoleoyl-GPE (16:0/18:2) levels | 8266 European |
| GCST90199845 | Sphinganine-1-phosphate levels | 8126 European |
| GCST90199846 | Glycosyl-N-stearoyl-sphingosine (d18:1/18:0) levels | 8233 European |
| GCST90199847 | 4-allylphenol sulfate levels | 8240 European |
| GCST90199848 | Succinylcarnitine levels | 8212 European |
| GCST90199849 | 21-hydroxypregnenolone disulfate levels | 8132 European |
| GCST90199850 | 5alpha-androstan-3alpha,17alpha-diol monosulfate levels | 5524 European |
| GCST90199851 | Androstenediol (3beta,17beta) disulfate (2) levels | 8205 European |
| GCST90199852 | 5alpha-androstan-3beta,17alpha-diol disulfate levels | 7103 European |
| GCST90199853 | 5alpha-pregnan-3beta,20beta-diol monosulfate (1) levels | 7772 European |
| GCST90199854 | 5alpha-pregnan-3beta,20alpha-diol monosulfate (2) levels | 8028 European |
| GCST90199855 | 5alpha-androstan-3beta,17beta-diol monosulfate (2) levels | 7066 European |
| GCST90199856 | Androstenediol (3alpha, 17alpha) monosulfate (3) levels | 8192 European |
| GCST90199857 | 5alpha-pregnan-diol disulfate levels | 4110 European |
| GCST90199858 | 5alpha-androstan-3alpha,17beta-diol monosulfate (1) levels | 7608 European |
| GCST90199859 | 5alpha-androstan-3alpha,17beta-diol disulfate levels | 5082 European |
| GCST90199860 | Andro steroid monosulfate C19H28O6S (1) levels | 7415 European |
| GCST90199861 | Pregnenediol sulfate (C21H34O5S) levels | 8260 European |
| GCST90199862 | 4-hydroxycoumarin levels | 6944 European |
| GCST90199863 | 16a-hydroxy DHEA 3-sulfate levels | 8236 European |
| GCST90199864 | Gamma-CEHC levels | 8258 European |
| GCST90199865 | 2-hydroxyglutarate levels | 7769 European |
| GCST90199866 | N-acetyl-beta-alanine levels | 8231 European |
| GCST90199867 | Tridecenedioate (C13:1-DC) levels | 8231 European |
| GCST90199868 | N-acetyl-3-methylhistidine levels | 7018 European |
| GCST90199869 | 2-hydroxyhippurate levels | 8241 European |
| GCST90199870 | 4-cholesten-3-one levels | 6990 European |
| GCST90199871 | Androstenediol (3beta,17beta) monosulfate (2) levels | 7811 European |
| GCST90199872 | Androstenediol (3beta,17beta) monosulfate (1) levels | 8229 European |
| GCST90199873 | Pregnenolone sulfate levels | 7772 European |
| GCST90199874 | S-methylmethionine levels | 6674 European |
| GCST90199875 | 13-HODE + 9-HODE levels | 8207 European |
| GCST90199876 | Palmitoyl sphingomyelin (d18:1/16:0) levels | 8269 European |
| GCST90199877 | Indole-3-carboxylate levels | 4989 European |
| GCST90199878 | Sphingomyelin (d18:1/18:1, d18:2/18:0) levels | 8267 European |
| GCST90199879 | Ergothioneine levels | 8210 European |
| GCST90199880 | Cis-4-decenoylcarnitine (C10:1) levels | 8239 European |
| GCST90199881 | 5-(galactosylhydroxy)-L-lysine levels | 6898 European |
| GCST90199882 | 2s,3R-dihydroxybutyrate levels | 8252 European |
| GCST90199883 | Cysteinylglycine disulfide levels | 8216 European |
| GCST90199884 | 2,3-dihydroxyisovalerate levels | 8268 European |
| GCST90199885 | Alpha-ketoglutaramate levels | 8210 European |
| GCST90199886 | 3-methylglutaconate levels | 8197 European |
| GCST90199887 | (16 or 17)-methylstearate (a19:0 or i19:0) levels | 8239 European |
| GCST90199888 | Cinnamoylglycine levels | 7824 European |
| GCST90199889 | 2R,3R-dihydroxybutyrate levels | 8223 European |
| GCST90199890 | 4-hydroxyglutamate levels | 7226 European |
| GCST90199891 | S-methylcysteine levels | 8239 European |
| GCST90199892 | Isoursodeoxycholate levels | 8234 European |
| GCST90199893 | Cis-4-decenoate (10:1n6) levels | 8173 European |
| GCST90199894 | 4-oxo-retinoic acid levels | 7514 European |
| GCST90199895 | 1-lignoceroyl-GPC (24:0) levels | 8208 European |
| GCST90199896 | Sulfate levels | 8230 European |
| GCST90199897 | 4-hydroxy-2-oxoglutaric acid levels | 7919 European |
| GCST90199898 | Glycoursodeoxycholate levels | 8252 European |
| GCST90199899 | 1-(1-enyl-palmitoyl)-GPC (p-16:0) levels | 8170 European |
| GCST90199900 | Argininate levels | 8226 European |
| GCST90199901 | 1-methyl-5-imidazoleacetate levels | 8171 European |
| GCST90199902 | Hydantoin-5-propionate levels | 8164 European |
| GCST90199903 | 2-oxoarginine levels | 8223 European |
| GCST90199904 | Pantoate levels | 7662 European |
| GCST90199905 | Formiminoglutamate levels | 8175 European |
| GCST90199906 | 1-(1-enyl-palmitoyl)-GPE (p-16:0) levels | 8227 European |
| GCST90199907 | S-methylcysteine sulfoxide levels | 8255 European |
| GCST90199908 | 2-o-methylascorbic acid levels | 8219 European |
| GCST90199909 | Eicosanedioate (C20-DC) levels | 8258 European |
| GCST90199910 | 1-(1-enyl-stearoyl)-GPE (p-18:0) levels | 8249 European |
| GCST90199911 | Beta-citrylglutamate levels | 8226 European |
| GCST90199912 | N-oleoyltaurine levels | 7375 European |
| GCST90199913 | Docosadioate (C22-DC) levels | 8225 European |
| GCST90199914 | Oleoyl-linoleoyl-glycerol (18:1/18:2) [2] levels | 8069 European |
| GCST90199915 | Imidazole propionate levels | 7776 European |
| GCST90199916 | Carnitine C18:2 levels | 8268 European |
| GCST90199917 | Pregnanediol-3-glucuronide levels | 7905 European |
| GCST90199918 | Carboxyethyl-gaba levels | 8090 European |
| GCST90199919 | Alliin levels | 6517 European |
| GCST90199920 | Trimethylamine n-oxide levels | 8218 European |
| GCST90199921 | Dihydroferulate levels | 6398 European |
| GCST90199922 | Prolylglycine levels | 7907 European |
| GCST90199923 | N-palmitoylglycine levels | 8236 European |
| GCST90199924 | N-methyltaurine levels | 5440 European |
| GCST90199925 | Succinimide levels | 5865 European |
| GCST90199926 | N-acetylcarnosine levels | 8233 European |
| GCST90199927 | Histidine betaine (hercynine) levels | 7518 European |
| GCST90199928 | 2-stearoyl-GPE (18:0) levels | 8073 European |
| GCST90199929 | Margaroylcarnitine (C17) levels | 8112 European |
| GCST90199930 | (R)-3-hydroxybutyrylcarnitine levels | 7787 European |
| GCST90199931 | Lanthionine levels | 7760 European |
| GCST90199932 | Mannonate levels | 8166 European |
| GCST90199933 | 3b-hydroxy-5-cholenoic acid levels | 6846 European |
| GCST90199934 | 2-hydroxydecanoate levels | 8267 European |
| GCST90199935 | Glycohyocholate levels | 7095 European |
| GCST90199936 | 3-methyl catechol sulfate (2) levels | 6320 European |
| GCST90199937 | 2-aminooctanoate levels | 8178 European |
| GCST90199938 | 3-methyl catechol sulfate (1) levels | 8233 European |
| GCST90199939 | 4-methylcatechol sulfate levels | 8229 European |
| GCST90199940 | Guaiacol sulfate levels | 8262 European |
| GCST90199941 | Gamma-CEHC glucuronide levels | 6305 European |
| GCST90199942 | Dimethyl sulfone levels | 7905 European |
| GCST90199943 | 2-piperidinone levels | 8225 European |
| GCST90199944 | Indolin-2-one levels | 7027 European |
| GCST90199945 | Sphingomyelin (d18:1/14:0, d16:1/16:0) levels | 8254 European |
| GCST90199946 | 3-acetylphenol sulfate levels | 6897 European |
| GCST90199947 | Sphingomyelin (d18:2/16:0, d18:1/16:1) levels | 8272 European |
| GCST90199948 | S-allylcysteine levels | 6857 European |
| GCST90199949 | 6-oxopiperidine-2-carboxylate levels | 8032 European |
| GCST90199950 | 2-aminophenol sulfate levels | 8264 European |
| GCST90199951 | N-acetylalliin levels | 5432 European |
| GCST90199952 | 2-aminoheptanoate levels | 8238 European |
| GCST90199953 | 3-methoxytyramine sulfate levels | 5419 European |
| GCST90199954 | N2,N5-diacetylornithine levels | 7596 European |
| GCST90199955 | Ferulic acid 4-sulfate levels | 7332 European |
| GCST90199956 | 1H-indole-7-acetic acid levels | 5986 European |
| GCST90199957 | 3-(3-hydroxyphenyl)propionate sulfate levels | 5773 European |
| GCST90199958 | 1-linolenoyl-GPC (18:3) levels | 8261 European |
| GCST90199959 | O-sulfo-l-tyrosine levels | 8229 European |
| GCST90199960 | N-formylanthranilic acid levels | 7534 European |
| GCST90199961 | Methionine sulfone levels | 8254 European |
| GCST90199962 | Acisoga levels | 7489 European |
| GCST90199963 | 17alpha-hydroxypregnanolone glucuronide levels | 5582 European |
| GCST90199964 | Etiocholanolone glucuronide levels | 7821 European |
| GCST90199965 | Fructosyllysine levels | 8238 European |
| GCST90199966 | 1-(1-enyl-oleoyl)-GPE (p-18:1) levels | 8157 European |
| GCST90199967 | N-acetyltaurine levels | 8160 European |
| GCST90199968 | 1-palmitoyl-GPG (16:0) levels | 7708 European |
| GCST90199969 | 1-oleoyl-GPG (18:1) levels | 6990 European |
| GCST90199970 | Octadecenedioylcarnitine (C18:1-DC) levels | 8245 European |
| GCST90199971 | 3-methylglutarylcarnitine (2) levels | 8220 European |
| GCST90199972 | Sphingomyelin (d18:1/24:1, d18:2/24:0) levels | 8267 European |
| GCST90199973 | Methyl glucopyranoside (alpha + beta) levels | 7973 European |
| GCST90199974 | 9-hydroxystearate levels | 7474 European |
| GCST90199975 | Sphingomyelin (d18:2/14:0, d18:1/14:1) levels | 8278 European |
| GCST90199976 | 1-dihomo-linolenylglycerol (20:3) levels | 6470 European |
| GCST90199977 | 4-hydroxychlorothalonil levels | 8216 European |
| GCST90199978 | Vanillactate levels | 7935 European |
| GCST90199979 | Carnitine C14:1 levels | 8257 European |
| GCST90199980 | N-formylphenylalanine levels | 7229 European |
| GCST90199981 | 2-acetamidophenol sulfate levels | 6984 European |
| GCST90199982 | 4-vinylguaiacol sulfate levels | 7592 European |
| GCST90199983 | 5alpha-androstan-3alpha,17beta-diol monosulfate (2) levels | 5920 European |
| GCST90199984 | Vanillic alcohol sulfate levels | 5076 European |
| GCST90199985 | Methyl-4-hydroxybenzoate sulfate levels | 8285 European |
| GCST90199986 | Eugenol sulfate levels | 7063 European |
| GCST90199987 | 2-methoxyresorcinol sulfate levels | 5897 European |
| GCST90199988 | Tyramine O-sulfate levels | 7254 European |
| GCST90199989 | 4-methylguaiacol sulfate levels | 7141 European |
| GCST90199990 | 3-hydroxypyridine sulfate levels | 8270 European |
| GCST90199991 | Arabonate/xylonate levels | 8211 European |
| GCST90199992 | Octadecanedioylcarnitine (C18-DC) levels | 8049 European |
| GCST90199993 | Phenylacetylcarnitine levels | 8127 European |
| GCST90199994 | Ethylparaben sulfate levels | 6408 European |
| GCST90199995 | Sphingomyelin (d18:1/20:2, d18:2/20:1, d16:1/22:2) levels | 8124 European |
| GCST90199996 | Sphingomyelin (d18:1/20:1, d18:2/20:0) levels | 8255 European |
| GCST90199997 | Behenoyl sphingomyelin (d18:1/22:0) levels | 8260 European |
| GCST90199998 | Propyl 4-hydroxybenzoate sulfate levels | 7855 European |
| GCST90199999 | 4-methoxyphenol sulfate levels | 7500 European |
| GCST90200000 | Umbelliferone sulfate levels | 5598 European |
| GCST90200001 | Sphingomyelin (d18:1/20:0, d16:1/22:0) levels | 8257 European |
| GCST90200002 | 6-hydroxyindole sulfate levels | 8229 European |
| GCST90200003 | P-cresol glucuronide levels | 7969 European |
| GCST90200004 | N-carbamoylalanine levels | 7315 European |
| GCST90200005 | Citraconate/glutaconate levels | 8263 European |
| GCST90200006 | 1,2,3-benzenetriol sulfate (2) levels | 7484 European |
| GCST90200007 | 3-methoxycatechol sulfate (2) levels | 7286 European |
| GCST90200008 | C-glycosyltryptophan levels | 8204 European |
| GCST90200009 | 3-methoxycatechol sulfate (1) levels | 8194 European |
| GCST90200010 | Sphingomyelin (d18:1/22:1, d18:2/22:0, d16:1/24:1) levels | 8246 European |
| GCST90200011 | Arabitol/xylitol levels | 8219 European |
| GCST90200012 | 3-hydroxyhexanoate levels | 8268 European |
| GCST90200013 | Sphingomyelin (d18:1/22:2, d18:2/22:1, d16:1/24:2) levels | 8267 European |
| GCST90200014 | 3beta-hydroxy-5-cholestenoate levels | 8259 European |
| GCST90200015 | N-acetylkynurenine (2) levels | 6193 European |
| GCST90200016 | Dopamine 4-sulfate levels | 6888 European |
| GCST90200017 | Sphingomyelin (d17:1/16:0, d18:1/15:0, d16:1/17:0) levels | 8261 European |
| GCST90200018 | Adipoylcarnitine (C6-DC) levels | 8216 European |
| GCST90200019 | Dopamine 3-o-sulfate levels | 8252 European |
| GCST90200020 | Lignoceroyl sphingomyelin (d18:1/24:0) levels | 8250 European |
| GCST90200021 | N-acetylglucosamine/n-acetylgalactosamine levels | 7940 European |
| GCST90200022 | Glycochenodeoxycholate 3-sulfate levels | 8123 European |
| GCST90200023 | Trans 3,4-methyleneheptanoate levels | 7104 European |
| GCST90200024 | Taurodeoxycholic acid 3-sulfate levels | 7333 European |
| GCST90200025 | Phenol glucuronide levels | 4466 European |
| GCST90200026 | Glycodeoxycholate 3-sulfate levels | 8066 European |
| GCST90200027 | 4-hydroxyphenylacetoylcarnitine levels | 7523 European |
| GCST90200028 | 1-stearoyl-2-oleoyl-gpc (18:0/18:1) levels | 8257 European |
| GCST90200029 | Linoleoyl ethanolamide levels | 7214 European |
| GCST90200030 | 1,2-dilinoleoyl-GPC (18:2/18:2) levels | 8227 European |
| GCST90200031 | Nonanoylcarnitine (C9) levels | 7669 European |
| GCST90200032 | 2-hydroxybutyrate/2-hydroxyisobutyrate levels | 8215 European |
| GCST90200033 | Sphingomyelin (d18:2/23:0, d18:1/23:1, d17:1/24:1) levels | 8272 European |
| GCST90200034 | Tricosanoyl sphingomyelin (d18:1/23:0) levels | 8257 European |
| GCST90200035 | Palmitoyl dihydrosphingomyelin (d18:0/16:0) levels | 8269 European |
| GCST90200036 | 1-palmitoleoylglycerol (16:1) levels | 6432 European |
| GCST90200037 | 1-stearoyl-2-linoleoyl-gpc (18:0/18:2) levels | 8231 European |
| GCST90200038 | 1-palmitoyl-2-palmitoleoyl-gpc (16:0/16:1) levels | 8276 European |
| GCST90200039 | 1-stearoyl-2-linoleoyl-GPE (18:0/18:2) levels | 8241 European |
| GCST90200040 | Sphingomyelin (d18:2/24:1, d18:1/24:2) levels | 8273 European |
| GCST90200041 | 1-palmitoyl-2-stearoyl-gpc (16:0/18:0) levels | 8271 European |
| GCST90200042 | Oleate/vaccenate (18:1) levels | 8230 European |
| GCST90200043 | 1-palmitoyl-2-docosahexaenoyl-gpc (16:0/22:6) levels | 8256 European |
| GCST90200044 | 1-(1-enyl-stearoyl)-2-oleoyl-GPE (p-18:0/18:1) levels | 8258 European |
| GCST90200045 | 1-stearoyl-2-arachidonoyl-GPE (18:0/20:4) levels | 8259 European |
| GCST90200046 | 1-stearoyl-2-docosahexaenoyl-gpc (18:0/22:6) levels | 8257 European |
| GCST90200047 | Sphingomyelin (d18:1/17:0, d17:1/18:0, d19:1/16:0) levels | 8252 European |
| GCST90200048 | 1-(1-enyl-palmitoyl)-2-arachidonoyl-GPE (p-16:0/20:4) levels | 8262 European |
| GCST90200049 | Myristoyl dihydrosphingomyelin (d18:0/14:0) levels | 8254 European |
| GCST90200050 | 5-hydroxyindole sulfate levels | 6163 European |
| GCST90200051 | 1-palmitoyl-2-dihomo-linolenoyl-GPC (16:0/20:3n3 or 6) levels | 8230 European |
| GCST90200052 | 1-(1-enyl-palmitoyl)-2-arachidonoyl-gpc (p-16:0/20:4) levels | 8251 European |
| GCST90200053 | Sphingomyelin (d18:1/21:0, d17:1/22:0, d16:1/23:0) levels | 8242 European |
| GCST90200054 | 1-palmitoyl-2-arachidonoyl-GPE (16:0/20:4) levels | 8257 European |
| GCST90200055 | Lactosyl-N-palmitoyl-sphingosine (d18:1/16:0) levels | 8266 European |
| GCST90200056 | Sphingomyelin (d18:0/18:0, d19:0/17:0) levels | 8274 European |
| GCST90200057 | 1-(1-enyl-palmitoyl)-2-oleoyl-gpc (p-16:0/18:1) levels | 8222 European |
| GCST90200058 | 1-(1-enyl-stearoyl)-2-arachidonoyl-GPE (p-18:0/20:4) levels | 8254 European |
| GCST90200059 | Behenoyl dihydrosphingomyelin (d18:0/22:0) levels | 8275 European |
| GCST90200060 | 1-(1-enyl-palmitoyl)-2-linoleoyl-GPC (p-16:0/18:2) levels | 8260 European |
| GCST90200061 | 1-(1-enyl-palmitoyl)-2-oleoyl-GPE (p-16:0/18:1) levels | 8264 European |
| GCST90200062 | 1-palmitoyl-2-arachidonoyl-GPI (16:0/20:4) levels | 8057 European |
| GCST90200063 | 1-palmitoyl-2-docosahexaenoyl-GPE (16:0/22:6) levels | 8262 European |
| GCST90200064 | N-palmitoyl-sphinganine (d18:0/16:0) levels | 8208 European |
| GCST90200065 | 1-stearoyl-2-docosahexaenoyl-GPE (18:0/22:6) levels | 8251 European |
| GCST90200066 | Gamma-tocopherol/beta-tocopherol levels | 8231 European |
| GCST90200067 | 1-stearoyl-2-linoleoyl-GPI (18:0/18:2) levels | 8113 European |
| GCST90200068 | 1,2-dilinoleoyl-GPE (18:2/18:2) levels | 6804 European |
| GCST90200069 | Phenylacetylglutamate levels | 6227 European |
| GCST90200070 | 1-(1-enyl-palmitoyl)-2-palmitoleoyl-GPC (P-16:0/16:1) levels | 8083 European |
| GCST90200071 | Palmitoylcholine levels | 8209 European |
| GCST90200072 | Thioproline levels | 8226 European |
| GCST90200073 | 1-linoleoyl-2-arachidonoyl-GPC (18:2/20:4n6) levels | 8063 European |
| GCST90200074 | 1-linoleoyl-GPG (18:2) levels | 8115 European |
| GCST90200075 | 1-(1-enyl-palmitoyl)-2-linoleoyl-GPE (p-16:0/18:2) levels | 8254 European |
| GCST90200076 | 1-(1-enyl-stearoyl)-2-linoleoyl-GPE (p-18:0/18:2) levels | 8261 European |
| GCST90200077 | 1-stearoyl-2-oleoyl-GPI (18:0/18:1) levels | 8111 European |
| GCST90200078 | 1-myristoyl-2-arachidonoyl-GPC (14:0/20:4) levels | 8264 European |
| GCST90200079 | 1-oleoyl-2-arachidonoyl-GPE (18:1/20:4) levels | 8121 European |
| GCST90200080 | 1-palmitoyl-2-oleoyl-GPI (16:0/18:1) levels | 8123 European |
| GCST90200081 | 1-oleoyl-2-docosahexaenoyl-GPC (18:1/22:6) levels | 8267 European |
| GCST90200082 | 1-oleoyl-2-linoleoyl-GPE (18:1/18:2) levels | 8205 European |
| GCST90200083 | 1-(1-enyl-palmitoyl)-2-palmitoyl-GPC (P-16:0/16:0) levels | 8266 European |
| GCST90200084 | Oleoylcholine levels | 8120 European |
| GCST90200085 | Glycosyl-N-palmitoyl-sphingosine (d18:1/16:0) levels | 8245 European |
| GCST90200086 | Arachidonoylcholine levels | 8170 European |
| GCST90200087 | 2-methylserine levels | 7722 European |
| GCST90200088 | (S)-3-hydroxybutyrylcarnitine levels | 8249 European |
| GCST90200089 | Methylsuccinoylcarnitine levels | 8004 European |
| GCST90200090 | Dihomo-linolenoyl-choline levels | 6954 European |
| GCST90200091 | Docosahexaenoylcholine levels | 7440 European |
| GCST90200092 | Glycocholate glucuronide (1) levels | 4381 European |
| GCST90200093 | Caffeic acid sulfate levels | 7798 European |
| GCST90200094 | Ascorbic acid 2-sulfate levels | 8089 European |
| GCST90200095 | 1-linoleoyl-2-linolenoyl-GPC (18:2/18:3) levels | 8188 European |
| GCST90200096 | Catechol glucuronide levels | 4698 European |
| GCST90200097 | Furaneol sulfate levels | 5882 European |
| GCST90200098 | Ceramide (d18:1/24:1) levels | 8265 European |
| GCST90200099 | N-oleoylserine levels | 7591 European |
| GCST90200100 | Perfluorooctanesulfonate (PFOS) levels | 8218 European |
| GCST90200101 | Pimeloylcarnitine/3-methyladipoylcarnitine (C7-DC) levels | 7837 European |
| GCST90200102 | 1-palmitoleoyl-2-linolenoyl-GPC (16:1/18:3) levels | 7540 European |
| GCST90200103 | Linoleoyl-arachidonoyl-glycerol (18:2/20:4) [1] levels | 7731 European |
| GCST90200104 | Hexadecadienoate (16:2n6) levels | 8274 European |
| GCST90200105 | Glycosyl-N-behenoyl-sphingadienine (d18:2/22:0) levels | 8232 European |
| GCST90200106 | 4-hydroxyphenylacetylglutamine levels | 7752 European |
| GCST90200107 | Gamma-glutamyl-alpha-lysine levels | 8250 European |
| GCST90200108 | N-palmitoyl-sphingadienine (d18:2/16:0) levels | 7805 European |
| GCST90200109 | Linoleoyl-arachidonoyl-glycerol (18:2/20:4) [2] levels | 7744 European |
| GCST90200110 | Palmitoleoylcarnitine (C16:1) levels | 8263 European |
| GCST90200111 | N-stearoyl-sphingadienine (d18:2/18:0) levels | 7229 European |
| GCST90200112 | 2,3-dihydroxy-2-methylbutyrate levels | 8018 European |
| GCST90200113 | Stearoylcholine levels | 7140 European |
| GCST90200114 | Glycosyl-N-tricosanoyl-sphingadienine (d18:2/23:0) levels | 7274 European |
| GCST90200115 | N-palmitoyl-heptadecasphingosine (d17:1/16:0) levels | 7141 European |
| GCST90200116 | Ceramide (d18:1/14:0, d16:1/16:0) levels | 7270 European |
| GCST90200117 | Glycosyl-N-(2-hydroxynervonoyl)-sphingosine (d18:1/24:1(2OH)) levels | 8012 European |
| GCST90200118 | Glycosyl ceramide (d18:2/24:1, d18:1/24:2) levels | 8251 European |
| GCST90200119 | Glycosyl ceramide (d18:1/23:1, d17:1/24:1) levels | 5309 European |
| GCST90200120 | Sphingadienine levels | 4533 European |
| GCST90200121 | Ceramide (d18:1/17:0, d17:1/18:0) levels | 6073 European |
| GCST90200122 | Nisinate (24:6n3) levels | 4765 European |
| GCST90200123 | Sphingomyelin (d18:0/20:0, d16:0/22:0) levels | 8277 European |
| GCST90200124 | Sphingomyelin (d18:2/24:2) levels | 8140 European |
| GCST90200125 | Sphingomyelin (d17:2/16:0, d18:2/15:0) levels | 8264 European |
| GCST90200126 | Sphingomyelin (d18:2/23:1) levels | 8267 European |
| GCST90200127 | Behenoylcarnitine (C22) levels | 7368 European |
| GCST90200128 | Sphingomyelin (d18:2/18:1) levels | 8253 European |
| GCST90200129 | Sphingomyelin (d18:2/21:0, d16:2/23:0) levels | 8260 European |
| GCST90200130 | Linoleoylcholine levels | 8193 European |
| GCST90200131 | Sphingomyelin (d18:1/19:0, d19:1/18:0) levels | 8256 European |
| GCST90200132 | Linolenoylcarnitine (C18:3) levels | 8195 European |
| GCST90200133 | Heneicosapentaenoate (21:5n3) levels | 4827 European |
| GCST90200134 | Sphingomyelin (d18:1/25:0, d19:0/24:1, d20:1/23:0, d19:1/24:0) levels | 8126 European |
| GCST90200135 | Arachidoylcarnitine (C20) levels | 7682 European |
| GCST90200136 | Cerotoylcarnitine (C26) levels | 8266 European |
| GCST90200137 | Lignoceroylcarnitine (C24) levels | 8238 European |
| GCST90200138 | Docosatrienoate (22:3n6) levels | 5398 European |
| GCST90200139 | Dihomo-linolenoylcarnitine (C20:3n3 or 6) levels | 8092 European |
| GCST90200140 | Carotene diol (2) levels | 8196 European |
| GCST90200141 | Ethyl alpha-glucopyranoside levels | 6620 European |
| GCST90200142 | Carotene diol (1) levels | 8256 European |
| GCST90200143 | Nervonoylcarnitine (C24:1) levels | 7880 European |
| GCST90200144 | Dihomo-linoleoylcarnitine (C20:2) levels | 7887 European |
| GCST90200145 | Glycosyl ceramide (d18:1/20:0, d16:1/22:0) levels | 8259 European |
| GCST90200146 | Docosahexaenoylcarnitine (C22:6) levels | 6287 European |
| GCST90200147 | N,N,N-trimethyl-5-aminovalerate levels | 8214 European |
| GCST90200148 | Eicosenoylcarnitine (C20:1) levels | 8145 European |
| GCST90200149 | Ximenoylcarnitine (C26:1) levels | 8260 European |
| GCST90200150 | Arachidonoylcarnitine (C20:4) levels | 8202 European |
| GCST90200151 | 5-dodecenoylcarnitine (C12:1) levels | 7899 European |
| GCST90200152 | 2-butenoylglycine levels | 6253 European |
| GCST90200153 | (N(1) + N(8))-acetylspermidine levels | 8219 European |
| GCST90200154 | Hydroxy-cmpf levels | 8254 European |
| GCST90200155 | 2-furoylcarnitine levels | 6448 European |
| GCST90200156 | Cortolone glucuronide (1) levels | 8152 European |
| GCST90200157 | Carotene diol (3) levels | 7650 European |
| GCST90200158 | 3-hydroxyoleoylcarnitine levels | 7532 European |
| GCST90200159 | Dodecenedioate (C12:1-DC) levels | 8217 European |
| GCST90200160 | Trans-2-hexenoylglycine levels | 6545 European |
| GCST90200161 | 2-hydroxyarachidate levels | 8170 European |
| GCST90200162 | 3-hydroxyphenylacetoylglutamine levels | 5371 European |
| GCST90200163 | Heptenedioate (C7:1-DC) levels | 8128 European |
| GCST90200164 | Hexadecenedioate (C16:1-DC) levels | 8248 European |
| GCST90200165 | Octadecenedioate (C18:1-DC) levels | 8267 European |
| GCST90200166 | Dihydroferulic acid sulfate levels | 5188 European |
| GCST90200167 | Glutamine conjugate of C7H12O2 levels | 7881 European |
| GCST90200168 | Hydroxyasparagine levels | 8206 European |
| GCST90200169 | Sphingomyelin (d17:1/14:0, d16:1/15:0) levels | 8267 European |
| GCST90200170 | Octadecadienedioate (C18:2-DC) levels | 8231 European |
| GCST90200171 | Perfluorooctanoate (PFOA) levels | 8199 European |
| GCST90200172 | 3-carboxy-4-methyl-5-pentyl-2-furanpropionate (3-CMPFP) levels | 8234 European |
| GCST90200173 | Glutamine conjugate of C6H10O2 (2) levels | 8233 European |
| GCST90200174 | 3-hydroxybutyroylglycine levels | 8171 European |
| GCST90200175 | Glycine conjugate of C10H14O2 (1) levels | 8128 European |
| GCST90200176 | Glutamine conjugate of C6H10O2 (1) levels | 7980 European |
| GCST90200177 | N-methylhydroxyproline levels | 6902 European |
| GCST90200178 | Glucuronide of C10H18O2 (7) levels | 6090 European |
| GCST90200179 | Glyco-beta-muricholate levels | 7206 European |
| GCST90200180 | Glucuronide of C12H22O4 (1) levels | 5831 European |
| GCST90200181 | N,n,n-trimethyl-alanylproline betaine (tmap) levels | 8220 European |
| GCST90200182 | Glycine conjugate of C10H12O2 levels | 6177 European |
| GCST90200183 | N-acetyl-2-aminooctanoate levels | 8253 European |
| GCST90200184 | 3-formylindole levels | 8256 European |
| GCST90200185 | Gamma-glutamylcitrulline levels | 8163 European |
| GCST90200186 | 8-methoxykynurenate levels | 6938 European |
| GCST90200187 | Tetradecadienoate (14:2) levels | 8255 European |
| GCST90200188 | 3-amino-2-piperidone levels | 8237 European |
| GCST90200189 | Glucuronide of piperine metabolite C17H21NO3 (3) levels | 6512 European |
| GCST90200190 | Dodecadienoate (12:2) levels | 8244 European |
| GCST90200191 | N-lactoyl tyrosine levels | 6389 European |
| GCST90200192 | N,N-dimethylalanine levels | 8219 European |
| GCST90200193 | Ethyl beta-glucopyranoside levels | 8152 European |
| GCST90200194 | N-lactoyl phenylalanine levels | 8223 European |
| GCST90200195 | N-acetyl-isoputreanine levels | 8236 European |
| GCST90200196 | 2,2'-Methylenebis(6-tert-butyl-p-cresol) levels | 7397 European |
| GCST90200197 | N-lactoyl isoleucine levels | 7526 European |
| GCST90200198 | 2-hydroxysebacate levels | 7865 European |
| GCST90200199 | Ascorbic acid 3-sulfate levels | 8186 European |
| GCST90200200 | N-lactoyl valine levels | 8133 European |
| GCST90200201 | 6-bromotryptophan levels | 8214 European |
| GCST90200202 | Delta-CEHC levels | 6233 European |
| GCST90200203 | Indoleacetoylcarnitine levels | 7777 European |
| GCST90200204 | N-acetyl leucine levels | 7646 European |
| GCST90200205 | 3-indoleglyoxylic acid levels | 7848 European |
| GCST90200206 | Sulfate of piperine metabolite C18H21NO3 (1) levels | 7021 European |
| GCST90200207 | Methyl vanillate sulfate levels | 4303 European |
| GCST90200208 | Glucuronide of piperine metabolite C17H21NO3 (4) levels | 7833 European |
| GCST90200209 | Sulfate of piperine metabolite C16H19NO3 (3) levels | 7705 European |
| GCST90200210 | Sulfate of piperine metabolite C16H19NO3 (2) levels | 7950 European |
| GCST90200211 | 3-hydroxypyridine glucuronide levels | 5882 European |
| GCST90200212 | Glucuronide of piperine metabolite C17H21NO3 (5) levels | 6582 European |
| GCST90200213 | 2-naphthol sulfate levels | 7673 European |
| GCST90200214 | 11beta-hydroxyandrosterone glucuronide levels | 8081 European |
| GCST90200215 | 4-ethylcatechol sulfate levels | 8222 European |
| GCST90200216 | Sulfate of piperine metabolite C18H21NO3 (3) levels | 7006 European |
| GCST90200217 | (2,4 or 2,5)-dimethylphenol sulfate levels | 5189 European |
| GCST90200218 | 11beta-hydroxyetiocholanolone glucuronide levels | 7220 European |
| GCST90200219 | Cholic acid glucuronide levels | 4414 European |
| GCST90200220 | 4-allylcatechol sulfate levels | 8139 European |
| GCST90200221 | Glycoursodeoxycholic acid sulfate (1) levels | 6622 European |
| GCST90200222 | Dihydrocaffeate sulfate (2) levels | 6519 European |
| GCST90200223 | 4-methylhexanoylglutamine levels | 5576 European |
| GCST90200224 | 2-ketocaprylate levels | 8240 European |
| GCST90200225 | 3-hydroxy-2-methylpyridine sulfate levels | 7446 European |
| GCST90200226 | 5-hydroxy-2-methylpyridine sulfate levels | 6977 European |
| GCST90200227 | 3-ethylcatechol sulfate (2) levels | 4814 European |
| GCST90200228 | 3-hydroxyhexanoylcarnitine (1) levels | 8227 European |
| GCST90200229 | 2,6-dihydroxybenzoic acid levels | 8269 European |
| GCST90200230 | 3-ethylcatechol sulfate (1) levels | 7062 European |
| GCST90200231 | Lithocholate sulfate (1) levels | 7891 European |
| GCST90200232 | 4-acetylcatechol sulfate (1) levels | 7565 European |
| GCST90200233 | 2,3-dihydroxy-5-methylthio-4-pentenoate (dmtpa) levels | 8225 European |
| GCST90200234 | Picolinoylglycine levels | 7862 European |
| GCST90200235 | Succinoyltaurine levels | 7897 European |
| GCST90200236 | Undecenoylcarnitine (C11:1) levels | 8253 European |
| GCST90200237 | Palmitoyl-sphingosine-phosphoethanolamine (d18:1/16:0) levels | 8140 European |
| GCST90200238 | Pregnenetriol disulfate levels | 8228 European |
| GCST90200239 | Tetradecadienedioate (C14:2-DC) levels | 7857 European |
| GCST90200240 | Hydroxy-N6,N6,N6-trimethyllysine levels | 8190 European |
| GCST90200241 | Pregnenetriol sulfate levels | 8233 European |
| GCST90200242 | Taurochenodeoxycholic acid 3-sulfate levels | 7157 European |
| GCST90200243 | Hydroxypalmitoyl sphingomyelin (d18:1/16:0(OH)) levels | 8260 European |
| GCST90200244 | 5-hydroxymethyl-2-furoylcarnitine levels | 4803 European |
| GCST90200245 | Eicosenedioate (C20:1-DC) levels | 7861 European |
| GCST90200246 | 3-hydroxydecanoylcarnitine levels | 8237 European |
| GCST90200247 | Levulinoylcarnitine levels | 7197 European |
| GCST90200248 | Cis 3,4-methyleneheptanoate levels | 8223 European |
| GCST90200249 | Branched-chain, straight-chain, or cyclopropyl 12:1 fatty acid levels | 8256 European |
| GCST90200250 | Deoxycholic acid 12-sulfate levels | 7438 European |
| GCST90200251 | Branched-chain, straight-chain, or cyclopropyl 10:1 fatty acid (1) levels | 8265 European |
| GCST90200252 | 3,5-dichloro-2,6-dihydroxybenzoic acid levels | 8218 European |
| GCST90200253 | Vanillic acid glycine levels | 7512 European |
| GCST90200254 | (S)-a-amino-omega-caprolactam levels | 8185 European |
| GCST90200255 | 3-bromo-5-chloro-2,6-dihydroxybenzoic acid levels | 8127 European |
| GCST90200256 | Dibutyl sulfosuccinate levels | 8099 European |
| GCST90200257 | Metabolonic lactone sulfate levels | 8203 European |
| GCST90200258 | 2-hydroxy-4-(methylthio)butanoic acid levels | 8255 European |
| GCST90200259 | Pentose acid levels | 8235 European |
| GCST90200260 | Branched chain 14:0 dicarboxylic acid levels | 7990 European |
| GCST90200261 | S-carboxyethylcysteine levels | 8010 European |
| GCST90200262 | N-succinyl-phenylalanine levels | 6199 European |
| GCST90200263 | (2 or 3)-decenoate (10:1n7 or n8) levels | 8124 European |
| GCST90200264 | Bilirubin degradation product, C17H20N2O5 (2) levels | 8254 European |
| GCST90200265 | Bilirubin degradation product, C16H18N2O5 (4) levels | 8159 European |
| GCST90200266 | Cis-3,4-methyleneheptanoylcarnitine levels | 8259 European |
| GCST90200267 | Tetrahydrocortisol glucuronide levels | 8182 European |
| GCST90200268 | N-acetyl-2-aminoadipate levels | 7606 European |
| GCST90200269 | Bilirubin degradation product, C17H20N2O5 (1) levels | 8252 European |
| GCST90200270 | 2-methoxyhydroquinone sulfate (1) levels | 7487 European |
| GCST90200271 | Bilirubin degradation product, C16H18N2O5 (3) levels | 7970 European |
| GCST90200272 | 3-hydroxyoctanoylcarnitine (1) levels | 8245 European |
| GCST90200273 | 2-methoxyhydroquinone sulfate (2) levels | 5778 European |
| GCST90200274 | Cis-3,4-methyleneheptanoylglycine levels | 8106 European |
| GCST90200275 | Bilirubin degradation product, C16H18N2O5 (2) levels | 8252 European |
| GCST90200276 | 2,4-di-tert-butylphenol levels | 8251 European |
| GCST90200277 | Bilirubin degradation product, C16H18N2O5 (1) levels | 7864 European |
| GCST90200278 | 3-hydroxyoctanoylcarnitine (2) levels | 8243 European |
| GCST90200279 | Pantothenate levels | 8194 European |
| GCST90200280 | 5-oxoproline levels | 8247 European |
| GCST90200281 | Phosphoethanolamine levels | 8253 European |
| GCST90200282 | Picolinate levels | 7051 European |
| GCST90200283 | 3-ureidopropionate levels | 7543 European |
| GCST90200284 | Erucate (22:1n9) levels | 8208 European |
| GCST90200285 | Eicosenedioate (C20:1-DC) levels | 8166 European |
| GCST90200286 | Allantoin levels | 8252 European |
| GCST90200287 | N-acetylvaline levels | 8218 European |
| GCST90200288 | N-acetylleucine levels | 7905 European |
| GCST90200289 | N-acetylmethionine levels | 7993 European |
| GCST90200290 | Glycerate levels | 8222 European |
| GCST90200291 | Sarcosine levels | 8223 European |
| GCST90200292 | Trans-4-hydroxyproline levels | 8252 European |
| GCST90200293 | Thyroxine levels | 8262 European |
| GCST90200294 | Pipecolate levels | 8161 European |
| GCST90200295 | Gamma-glutamyltyrosine levels | 8221 European |
| GCST90200296 | Methyl indole-3-acetate levels | 7032 European |
| GCST90200297 | Ursodeoxycholate levels | 6206 European |
| GCST90200298 | 3-hydroxy-3-methylglutarate levels | 8073 European |
| GCST90200299 | Oleoyl ethanolamide levels | 8243 European |
| GCST90200300 | 4-hydroxyphenylpyruvate levels | 7813 European |
| GCST90200301 | Gamma-glutamylglutamine levels | 8217 European |
| GCST90200302 | Alpha-tocopherol levels | 8192 European |
| GCST90200303 | N-acetyl-L-alanine levels | 8220 European |
| GCST90200304 | Citrate levels | 8260 European |
| GCST90200305 | 2-aminobutyrate levels | 8242 European |
| GCST90200306 | Butyrate/isobutyrate (4:0) levels | 8004 European |
| GCST90200307 | Urate levels | 8264 European |
| GCST90200308 | 3-hydroxyisobutyrate levels | 8177 European |
| GCST90200309 | Vanillylmandelate (VMA) levels | 8216 European |
| GCST90200310 | 3-aminoisobutyrate levels | 8265 European |
| GCST90200311 | 4-acetamidobutanoate levels | 8154 European |
| GCST90200312 | 2-hydroxystearate levels | 8241 European |
| GCST90200313 | N1-methyladenosine levels | 8247 European |
| GCST90200314 | N-acetylneuraminate levels | 8200 European |
| GCST90200315 | Gamma-glutamylhistidine levels | 8220 European |
| GCST90200316 | N-acetylglucosaminylasparagine levels | 8144 European |
| GCST90200317 | Homovanillate (hva) levels | 6320 European |
| GCST90200318 | Acetoacetate levels | 6883 European |
| GCST90200319 | Cys-gly, oxidized levels | 8256 European |
| GCST90200320 | Dihomo-linoleate (20:2n6) levels | 8284 European |
| GCST90200321 | Cholate levels | 7892 European |
| GCST90200322 | Beta-hydroxyisovalerate levels | 8201 European |
| GCST90200323 | Creatine levels | 8285 European |
| GCST90200324 | Anthranilate levels | 5773 European |
| GCST90200325 | Glycerol levels | 8285 European |
| GCST90200326 | Gamma-glutamylleucine levels | 8041 European |
| GCST90200327 | Choline levels | 8262 European |
| GCST90200328 | 3-methoxytyrosine levels | 8258 European |
| GCST90200329 | 3-phosphoglycerate levels | 8196 European |
| GCST90200330 | 1-palmitoyl-2-linoleoyl-gpc (16:0/18:2) levels | 8230 European |
| GCST90200331 | 1-palmitoyl-2-oleoyl-GPE (16:0/18:1) levels | 8262 European |
| GCST90200332 | 1-palmitoyl-2-linoleoyl-GPI (16:0/18:2) levels | 8115 European |
| GCST90200333 | Taurocholic acid levels | 7743 European |
| GCST90200334 | Taurodeoxycholate levels | 7287 European |
| GCST90200335 | Stearoyl sphingomyelin (d18:1/18:0) levels | 8258 European |
| GCST90200336 | Ceramide (d18:1/16:0) levels | 8266 European |
| GCST90200337 | Taurochenodeoxycholate levels | 8048 European |
| GCST90200338 | 4-hydroxyphenylacetate levels | 7906 European |
| GCST90200339 | 5,6-dihydrothymine levels | 8219 European |
| GCST90200340 | 1-palmitoyl-2-oleoyl-gpc (16:0/18:1) levels | 8249 European |
| GCST90200341 | N-stearoyl-sphingosine (d18:1/18:0) levels | 8254 European |
| GCST90200342 | Glycochenodeoxycholate levels | 8164 European |
| GCST90200343 | N-formylmethionine levels | 8235 European |
| GCST90200344 | N6,n6,n6-trimethyllysine levels | 8180 European |
| GCST90200345 | S-adenosylhomocysteine (SAH) levels | 6142 European |
| GCST90200346 | 4-acetaminophen sulfate levels | 4197 European |
| GCST90200347 | 2-hydroxyhippurate (salicylurate) levels | 8244 European |
| GCST90200348 | Quinolinate levels | 8203 European |
| GCST90200349 | Eicosapentaenoate (EPA; 20:5n3) levels | 8257 European |
| GCST90200350 | Laurate (12:0) levels | 8226 European |
| GCST90200351 | N-acetylputrescine levels | 8243 European |
| GCST90200352 | Methylsuccinate levels | 7965 European |
| GCST90200353 | 9,10-DiHOME levels | 7557 European |
| GCST90200354 | Linoleate (18:2n6) levels | 8260 European |
| GCST90200355 | Adenosine 5'-diphosphate (ADP) levels | 4607 European |
| GCST90200356 | Adenosine 5'-monophosphate (AMP) levels | 8218 European |
| GCST90200357 | Adenosine 3',5'-cyclic monophosphate (camp) levels | 6756 European |
| GCST90200358 | Arachidonate (20:4n6) levels | 8272 European |
| GCST90200359 | 5-methylthioadenosine (mta) levels | 8210 European |
| GCST90200360 | Cortisone levels | 8153 European |
| GCST90200361 | Beta-alanine levels | 8114 European |
| GCST90200362 | Succinate levels | 7943 European |
| GCST90200363 | Phosphocholine levels | 8245 European |
| GCST90200364 | Cysteinylglycine levels | 8214 European |
| GCST90200365 | Phenylpyruvate levels | 8241 European |
| GCST90200366 | Creatinine levels | 8239 European |
| GCST90200367 | 3-Hydroxybutyrate levels | 8292 European |
| GCST90200368 | Cholesterol levels | 8258 European |
| GCST90200369 | 2-hydroxyphenylacetate levels | 7564 European |
| GCST90200370 | Aspartate levels | 8253 European |
| GCST90200371 | 3-(4-hydroxyphenyl)lactate levels | 8259 European |
| GCST90200372 | Arginine levels | 8237 European |
| GCST90200373 | Gluconate levels | 8106 European |
| GCST90200374 | Sphinganine levels | 7429 European |
| GCST90200375 | Gamma-glutamylglutamate levels | 8244 European |
| GCST90200376 | Flavin adenine dinucleotide (FAD) levels | 6257 European |
| GCST90200377 | Histidine levels | 8223 European |
| GCST90200378 | Cortisol levels (plasma) | 8193 European |
| GCST90200379 | Hypotaurine levels | 8212 European |
| GCST90200380 | Glutarate (C5-DC) levels | 7757 European |
| GCST90200381 | S-1-pyrroline-5-carboxylate levels | 8232 European |
| GCST90200382 | Cystine levels | 8177 European |
| GCST90200383 | Cystathionine levels | 8014 European |
| GCST90200384 | Guanidinoacetate levels | 8238 European |
| GCST90200385 | Sphingosine levels | 7982 European |
| GCST90200386 | Deoxycholate levels | 7649 European |
| GCST90200387 | Glycocholate levels | 8247 European |
| GCST90200388 | Palmitoleate (16:1n7) levels | 8290 European |
| GCST90200389 | Leucine levels | 8252 European |
| GCST90200390 | Stearate (18:0) levels | 8272 European |
| GCST90200391 | Methionine levels | 8222 European |
| GCST90200392 | Orotate levels | 8128 European |
| GCST90200393 | Phosphate levels (UKB data field 30810) | 8247 European |
| GCST90200394 | Inosine levels | 4940 European |
| GCST90200395 | Inosine 5'-monophosphate (IMP) levels | 4639 European |
| GCST90200396 | Phenylalanine levels | 8228 European |
| GCST90200397 | Palmitate (16:0) levels | 8269 European |
| GCST90200398 | Malate levels | 8187 European |
| GCST90200399 | Isoleucine levels | 8255 European |
| GCST90200400 | Nicotinamide levels | 8110 European |
| GCST90200401 | Myo-inositol levels | 8193 European |
| GCST90200402 | Lysine levels | 8250 European |
| GCST90200403 | Citrulline levels | 8157 European |
| GCST90200404 | Ornithine levels | 8255 European |
| GCST90200405 | Retinol (Vitamin A) levels | 8247 European |
| GCST90200406 | 1-methylnicotinamide levels | 8194 European |
| GCST90200407 | Uridine levels | 8253 European |
| GCST90200408 | Plasma lactate levels | 8217 European |
| GCST90200409 | Pristanate levels | 6497 European |
| GCST90200410 | Phytanate levels | 8223 European |
| GCST90200411 | Plasma free proline levels | 8257 European |
| GCST90200412 | Glutamate levels | 8287 European |
| GCST90200413 | 2'-deoxyuridine levels | 7915 European |
| GCST90200414 | Myristate (14:0) levels | 8282 European |
| GCST90200415 | Serine levels | 8271 European |
| GCST90200416 | Trans-urocanate levels | 8198 European |
| GCST90200417 | Urea levels | 8238 European |
| GCST90200418 | Pyridoxal levels | 8021 European |
| GCST90200419 | Glutamine levels | 8253 European |
| GCST90200420 | Taurine levels | 8219 European |
| GCST90200421 | Salicylate levels | 8191 European |
| GCST90200422 | Serotonin levels | 7498 European |
| GCST90200423 | Spermidine levels | 7348 European |
| GCST90200424 | Dimethylglycine levels | 8224 European |
| GCST90200425 | Betaine levels | 8232 European |
| GCST90200426 | Xylose levels | 6822 European |
| GCST90200427 | Tyrosine levels | 8252 European |
| GCST90200428 | Caprate (10:0) levels | 8272 European |
| GCST90200429 | Cytidine levels | 7659 European |
| GCST90200430 | Pyruvate levels | 8259 European |
| GCST90200431 | Alanine levels | 8265 European |
| GCST90200432 | Threonine levels | 8245 European |
| GCST90200433 | Cytosine levels | 6990 European |
| GCST90200434 | 12,13-DiHOME levels | 7918 European |
| GCST90200435 | Mannose levels | 8212 European |
| GCST90200436 | Caffeine levels | 8005 European |
| GCST90200437 | Fructose levels | 8137 European |
| GCST90200438 | Alpha-ketobutyrate levels | 8192 European |
| GCST90200439 | Cysteine levels | 8216 European |
| GCST90200440 | Blood sugar levels | 8123 European |
| GCST90200441 | Tryptophan levels | 8235 European |
| GCST90200442 | Valine levels | 8247 European |
| GCST90200443 | Pseudouridine levels | 8213 European |
| GCST90200444 | Dihydroorotate levels | 8195 European |
| GCST90200445 | Caprylate (8:0) levels | 8163 European |
| GCST90200446 | Arachidate (20:0) levels | 8228 European |
| GCST90200447 | Maltose levels in coronary artery disease | 6351 European |
| GCST90200448 | N-stearoyl-sphinganine (d18:0/18:0) levels | 6737 European |
| GCST90200449 | Sucrose levels | 7803 European |
| GCST90200450 | Caproate (6:0) levels | 7564 European |
| GCST90200451 | Nonadecanoate (19:0) levels | 8231 European |
| GCST90200452 | Plasma free asparagine levels | 8245 European |
| GCST90200453 | X-07765 levels | 8127 European |
| GCST90200454 | Kynurenate levels | 8241 European |
| GCST90200455 | Alpha-ketoglutarate levels | 8212 European |
| GCST90200456 | X-11308 levels | 8253 European |
| GCST90200457 | Pentadecanoate (15:0) levels | 8273 European |
| GCST90200458 | X-11315 levels | 8139 European |
| GCST90200459 | Margarate (17:0) levels | 8279 European |
| GCST90200460 | X-11372 levels | 8251 European |
| GCST90200461 | X-10458 levels | 7970 European |
| GCST90200462 | X-11299 levels | 8203 European |
| GCST90200463 | X-11478 levels | 8242 European |
| GCST90200464 | X-11849 levels | 7679 European |
| GCST90200465 | X-11787 levels | 8247 European |
| GCST90200466 | X-11632 levels | 7784 European |
| GCST90200467 | X-11852 levels | 7295 European |
| GCST90200468 | X-11847 levels | 7193 European |
| GCST90200469 | X-11483 levels | 7798 European |
| GCST90200470 | X-11470 levels | 8134 European |
| GCST90200471 | X-11381 levels | 8225 European |
| GCST90200472 | X-11850 levels | 7629 European |
| GCST90200473 | X-11795 levels | 8238 European |
| GCST90200474 | X-11444 levels | 8206 European |
| GCST90200475 | X-11858 levels | 6008 European |
| GCST90200476 | X-11843 levels | 7230 European |
| GCST90200477 | X-12127 levels | 5878 European |
| GCST90200478 | X-12221 levels | 6819 European |
| GCST90200479 | X-12283 levels | 7763 European |
| GCST90200480 | X-12410 levels | 7442 European |
| GCST90200481 | X-12104 levels | 7194 European |
| GCST90200482 | X-12193 levels | 7705 European |
| GCST90200483 | X-12216 levels | 8181 European |
| GCST90200484 | X-12306 levels | 6054 European |
| GCST90200485 | X-12100 levels | 8210 European |
| GCST90200486 | X-12261 levels | 4347 European |
| GCST90200487 | X-11880 levels | 8206 European |
| GCST90200488 | X-12117 levels | 7379 European |
| GCST90200489 | X-12407 levels | 6534 European |
| GCST90200490 | X-12101 levels | 8217 European |
| GCST90200491 | X-12026 levels | 8180 European |
| GCST90200492 | X-12411 levels | 8174 European |
| GCST90200493 | X-12013 levels | 5848 European |
| GCST90200494 | X-12007 levels | 8002 European |
| GCST90200495 | X-12462 levels | 8236 European |
| GCST90200496 | X-12714 levels | 6870 European |
| GCST90200497 | X-12740 levels | 6449 European |
| GCST90200498 | X-12730 levels | 7133 European |
| GCST90200499 | X-12701 levels | 6278 European |
| GCST90200500 | X-12816 levels | 5190 European |
| GCST90200501 | X-12731 levels | 5770 European |
| GCST90200502 | X-12707 levels | 7461 European |
| GCST90200503 | X-12729 levels | 7446 European |
| GCST90200504 | X-12830 levels | 5725 European |
| GCST90200505 | X-12818 levels | 6246 European |
| GCST90200506 | X-12812 levels | 6435 European |
| GCST90200507 | X-12822 levels | 7957 European |
| GCST90200508 | X-12738 levels | 5874 European |
| GCST90200509 | X-12544 levels | 7471 European |
| GCST90200510 | X-12680 levels | 8255 European |
| GCST90200511 | X-12906 levels | 7896 European |
| GCST90200512 | X-13684 levels | 8112 European |
| GCST90200513 | X-13507 levels | 7936 European |
| GCST90200514 | X-12851 levels | 7385 European |
| GCST90200515 | X-12849 levels | 7555 European |
| GCST90200516 | X-12847 levels | 7790 European |
| GCST90200517 | X-13553 levels | 8118 European |
| GCST90200518 | X-12839 levels | 4867 European |
| GCST90200519 | X-13729 levels | 7724 European |
| GCST90200520 | X-13007 levels | 6270 European |
| GCST90200521 | X-13844 levels | 7501 European |
| GCST90200522 | X-13728 levels | 7905 European |
| GCST90200523 | X-12844 levels | 8231 European |
| GCST90200524 | X-13695 levels | 6486 European |
| GCST90200525 | X-13723 levels | 5269 European |
| GCST90200526 | X-14056 levels | 8242 European |
| GCST90200527 | X-13866 levels | 8252 European |
| GCST90200528 | X-17146 levels | 7778 European |
| GCST90200529 | X-16397 levels | 8057 European |
| GCST90200530 | X-17335 levels | 7747 European |
| GCST90200531 | X-16964 levels | 8048 European |
| GCST90200532 | X-16935 levels | 7804 European |
| GCST90200533 | X-15486 levels | 8236 European |
| GCST90200534 | X-16580 levels | 8254 European |
| GCST90200535 | X-17325 levels | 7448 European |
| GCST90200536 | X-17328 levels | 5856 European |
| GCST90200537 | X-16087 levels | 7071 European |
| GCST90200538 | X-17010 levels | 7879 European |
| GCST90200539 | X-14939 levels | 8250 European |
| GCST90200540 | X-15461 levels | 8144 European |
| GCST90200541 | X-15523 levels | 8071 European |
| GCST90200542 | X-15728 levels | 7874 European |
| GCST90200543 | X-15503 levels | 8237 European |
| GCST90200544 | X-16124 levels | 5337 European |
| GCST90200545 | X-17438 levels | 6913 European |
| GCST90200546 | X-17354 levels | 5375 European |
| GCST90200547 | X-17654 levels | 7690 European |
| GCST90200548 | X-17676 levels | 8118 European |
| GCST90200549 | X-17367 levels | 6092 European |
| GCST90200550 | X-17357 levels | 8041 European |
| GCST90200551 | X-18886 levels | 8198 European |
| GCST90200552 | X-18887 levels | 6790 European |
| GCST90200553 | X-17653 levels | 8025 European |
| GCST90200554 | X-17346 levels | 6459 European |
| GCST90200555 | X-17690 levels | 7043 European |
| GCST90200556 | X-18345 levels | 5476 European |
| GCST90200557 | X-17685 levels | 7327 European |
| GCST90200558 | X-18888 levels | 6974 European |
| GCST90200559 | X-18901 levels | 8128 European |
| GCST90200560 | X-17351 levels | 7281 European |
| GCST90200561 | X-18779 levels | 8144 European |
| GCST90200562 | X-21286 levels | 8231 European |
| GCST90200563 | X-21355 levels | 8257 European |
| GCST90200564 | X-21319 levels | 8109 European |
| GCST90200565 | X-21258 levels | 8198 European |
| GCST90200566 | X-21364 levels | 8203 European |
| GCST90200567 | X-21353 levels | 8265 European |
| GCST90200568 | X-19438 levels | 7259 European |
| GCST90200569 | X-18913 levels | 8271 European |
| GCST90200570 | X-18921 levels | 8257 European |
| GCST90200571 | X-21339 levels | 8203 European |
| GCST90200572 | X-19299 levels | 6100 European |
| GCST90200573 | X-18935 levels | 4952 European |
| GCST90200574 | X-21310 levels | 7801 European |
| GCST90200575 | X-21283 levels | 7055 European |
| GCST90200576 | X-21285 levels | 6180 European |
| GCST90200577 | X-21312 levels | 4897 European |
| GCST90200578 | X-18922 levels | 8174 European |
| GCST90200579 | X-21442 levels | 6827 European |
| GCST90200580 | X-21796 levels | 8130 European |
| GCST90200581 | X-21441 levels | 8030 European |
| GCST90200582 | X-21752 levels | 6526 European |
| GCST90200583 | X-21807 levels | 5602 European |
| GCST90200584 | X-21736 levels | 8232 European |
| GCST90200585 | X-21829 levels | 8217 European |
| GCST90200586 | X-21471 levels | 7982 European |
| GCST90200587 | X-21733 levels | 6916 European |
| GCST90200588 | X-21742 levels | 5918 European |
| GCST90200589 | X-21821 levels | 7699 European |
| GCST90200590 | X-21470 levels | 7700 European |
| GCST90200591 | X-21383 levels | 8226 European |
| GCST90200592 | X-21607 levels | 7975 European |
| GCST90200593 | X-21834 levels | 7466 European |
| GCST90200594 | X-21467 levels | 8195 European |
| GCST90200595 | X-22776 levels | 6944 European |
| GCST90200596 | X-23655 levels | 6764 European |
| GCST90200597 | X-22834 levels | 6531 European |
| GCST90200598 | X-22771 levels | 8087 European |
| GCST90200599 | X-23639 levels | 8272 European |
| GCST90200600 | X-23648 levels | 7357 European |
| GCST90200601 | X-23654 levels | 8241 European |
| GCST90200602 | X-22520 levels | 5389 European |
| GCST90200603 | X-23641 levels | 6673 European |
| GCST90200604 | X-23276 levels | 5407 European |
| GCST90200605 | X-21845 levels | 4874 European |
| GCST90200606 | X-22509 levels | 5423 European |
| GCST90200607 | X-23644 levels | 8250 European |
| GCST90200608 | X-22162 levels | 8249 European |
| GCST90200609 | X-23587 levels | 7662 European |
| GCST90200610 | X-23593 levels | 7988 European |
| GCST90200611 | X-23636 levels | 8251 European |
| GCST90200612 | X-23678 levels | 5745 European |
| GCST90200613 | X-23680 levels | 8215 European |
| GCST90200614 | X-23780 levels | 7878 European |
| GCST90200615 | X-23974 levels | 7752 European |
| GCST90200616 | X-23659 levels | 8176 European |
| GCST90200617 | X-23665 levels | 8214 European |
| GCST90200618 | X-23782 levels | 8263 European |
| GCST90200619 | X-24295 levels | 7987 European |
| GCST90200620 | X-24243 levels | 7540 European |
| GCST90200621 | X-23739 levels | 8249 European |
| GCST90200622 | X-23997 levels | 7804 European |
| GCST90200623 | X-24241 levels | 8053 European |
| GCST90200624 | X-24328 levels | 7807 European |
| GCST90200625 | X-24456 levels | 6523 European |
| GCST90200626 | X-24418 levels | 5952 European |
| GCST90200627 | X-24344 levels | 5457 European |
| GCST90200628 | X-24556 levels | 8004 European |
| GCST90200629 | X-24337 levels | 8088 European |
| GCST90200630 | X-24546 levels | 6391 European |
| GCST90200631 | X-24306 levels | 8074 European |
| GCST90200632 | X-24307 levels | 8119 European |
| GCST90200633 | X-24334 levels | 6975 European |
| GCST90200634 | X-24518 levels | 6131 European |
| GCST90200635 | X-24494 levels | 6767 European |
| GCST90200636 | X-24544 levels | 7939 European |
| GCST90200637 | X-24531 levels | 6455 European |
| GCST90200638 | X-24811 levels | 6349 European |
| GCST90200639 | X-24757 levels | 6251 European |
| GCST90200640 | X-24978 levels | 5151 European |
| GCST90200641 | X-24970 levels | 8263 European |
| GCST90200642 | X-24795 levels | 4986 European |
| GCST90200643 | X-24951 levels | 8208 European |
| GCST90200644 | X-24728 levels | 8160 European |
| GCST90200645 | X-24565 levels | 4364 European |
| GCST90200646 | X-24801 levels | 8059 European |
| GCST90200647 | X-24736 levels | 4846 European |
| GCST90200648 | X-24585 levels | 8241 European |
| GCST90200649 | X-24588 levels | 8239 European |
| GCST90200650 | X-25109 levels | 7477 European |
| GCST90200651 | X-24947 levels | 8002 European |
| GCST90200652 | X-24949 levels | 7174 European |
| GCST90200653 | X-24812 levels | 7878 European |
| GCST90200654 | X-25217 levels | 4098 European |
| GCST90200655 | X-25828 levels | 8156 European |
| GCST90200656 | X-25371 levels | 8205 European |
| GCST90200657 | X-25419 levels | 7994 European |
| GCST90200658 | X-25265 levels | 7916 European |
| GCST90200659 | X-25172 levels | 8117 European |
| GCST90200660 | X-25957 levels | 7335 European |
| GCST90200661 | X-25422 levels | 8210 European |
| GCST90200662 | X-25790 levels | 8221 European |
| GCST90200663 | X-25420 levels | 8271 European |
| GCST90200664 | X-25810 levels | 8167 European |
| GCST90200665 | X-25433 levels | 6516 European |
| GCST90200666 | X-25343 levels | 8195 European |
| GCST90200667 | X-25271 levels | 7906 European |
| GCST90200668 | X-25520 levels | 7997 European |
| GCST90200669 | X-25519 levels | 8080 European |
| GCST90200670 | X-26111 levels | 8002 European |
| GCST90200671 | X-26109 levels | 6992 European |
| GCST90200672 | X-26054 levels | 8046 European |
| GCST90200673 | Carnitine C4 levels | 8111 European |
| GCST90200674 | Androsterone sulfate levels | 8182 European |
| GCST90200675 | N-acetyltyrosine levels | 7841 European |
| GCST90200676 | N-acetyl-L-glutamine levels | 8181 European |
| GCST90200677 | N-acetylphenylalanine levels | 8228 European |
| GCST90200678 | N-acetylasparagine levels | 7935 European |
| GCST90200679 | N-acetylarginine levels | 8271 European |
| GCST90200680 | 5-acetylamino-6-formylamino-3-methyluracil levels | 7599 European |
| GCST90200681 | Orotidine levels | 8017 European |
| GCST90200682 | 3-methylcytidine levels | 8117 European |
| GCST90200683 | N-acetylcitrulline levels | 7324 European |
| GCST90200684 | N-acetyl-aspartyl-glutamate (naag) levels | 7705 European |
| GCST90200685 | 1-stearoyl-2-arachidonoyl-gpc (18:0/20:4) levels | 8253 European |
| GCST90200686 | Bilirubin (E,E) levels | 8263 European |
| GCST90200687 | Bilirubin (E,Z or Z,E) levels | 8238 European |
| GCST90200688 | Androsterone glucuronide levels | 7980 European |
| GCST90200689 | N6-methyllysine levels | 8299 European |
| GCST90200690 | N-acetyl-1-methylhistidine levels | 7588 European |
| GCST90200691 | N-delta-acetylornithine levels | 8259 European |
| GCST90200692 | 1-palmitoyl-2-arachidonoyl-gpc (16:0/20:4n6) levels | 8238 European |
| GCST90200693 | Glycochenodeoxycholate glucuronide (1) levels | 8236 European |
| GCST90200694 | 2'-o-methylcytidine levels | 8122 European |
| GCST90200695 | 2'-o-methyluridine levels | 7582 European |
| GCST90200696 | N6,N6-dimethyllysine levels | 8265 European |
| GCST90200697 | N2-acetyl,N6,N6-dimethyllysine levels | 7891 European |
| GCST90200698 | N2-acetyl,N6-methyllysine levels | 8069 European |
| GCST90200699 | Deoxycholic acid glucuronide levels | 8060 European |
| GCST90200700 | Decadienedioic acid (C10:2-DC) levels | 8131 European |
| GCST90200701 | Bilirubin degradation product, C17H18N2O4 (1) levels | 8254 European |
| GCST90200702 | Bilirubin degradation product, C17H18N2O4 (2) levels | 8255 European |
| GCST90200703 | Bilirubin degradation product, C17H18N2O4 (3) levels | 8253 European |
| GCST90200704 | Bilirubin (z,z) levels | 8261 European |
| GCST90200705 | Ethylmalonate levels | 8172 European |
| GCST90200706 | Biliverdin levels | 8262 European |
| GCST90200707 | Glycine levels | 8262 European |
| GCST90200708 | X-12112 levels | 8211 European |
| GCST90200709 | X-12798 levels | 8186 European |
| GCST90200710 | X-13431 levels | 8161 European |
| GCST90200711 | X-19141 levels | 8244 European |
| GCST90200712 | Spermidine to 5-methylthioadenosine (MTA) ratio | 7320 European |
| GCST90200713 | Spermidine to ornithine ratio | 7309 European |
| GCST90200714 | Alpha-ketoglutarate to alpha-ketobutyrate ratio | 8216 European |
| GCST90200715 | Alpha-ketoglutarate to glutamate ratio | 8274 European |
| GCST90200716 | Adenosine 5'-diphosphate (ADP) to phosphoethanolamine ratio | 4579 European |
| GCST90200717 | 3-phosphoglycerate to adenosine 5'-diphosphate (ADP) ratio | 4530 European |
| GCST90200718 | Alpha-ketoglutarate to succinate ratio | 8260 European |
| GCST90200719 | Adenosine 3',5'-cyclic monophosphate (cAMP) to adenosine 5'-monophosphate (AMP) ratio | 6726 European |
| GCST90200720 | Adenosine 5'-diphosphate (ADP) to pantothenate ratio | 4561 European |
| GCST90200721 | Adenosine 5'-diphosphate (ADP) to phosphate ratio | 4559 European |
| GCST90200722 | S-adenosylhomocysteine (SAH) to leucine ratio | 6122 European |
| GCST90200723 | S-adenosylhomocysteine (SAH) to 5-methyluridine (ribothymidine) ratio | 6078 European |
| GCST90200724 | Cholate to taurocholate ratio | 7328 European |
| GCST90200725 | Adenosine 5'-diphosphate (ADP) to creatine ratio | 4561 European |
| GCST90200726 | N-acetylputrescine to (N(1) + N(8))-acetylspermidine ratio | 8144 European |
| GCST90200727 | Adenosine 3',5'-cyclic monophosphate (cAMP) to taurocholate ratio | 6329 European |
| GCST90200728 | Adenosine 5'-diphosphate (ADP) to Adenosine 5'-monophosphate (AMP) ratio | 4573 European |
| GCST90200729 | 3-phosphoglycerate to phosphate ratio | 8153 European |
| GCST90200730 | Adenosine 5'-diphosphate (ADP) to fructose ratio | 4571 European |
| GCST90200731 | Adenosine 5'-diphosphate (ADP) to mannose ratio | 4587 European |
| GCST90200732 | Adenosine 5'-diphosphate (ADP) to tyrosine ratio | 4560 European |
| GCST90200733 | Adenosine 5'-diphosphate (ADP) to N-acetylglucosamine to N-acetylgalactosamine ratio | 4392 European |
| GCST90200734 | Adenosine 5'-monophosphate (AMP) to palmitate (16:0) ratio | 8227 European |
| GCST90200735 | Arginine to ornithine ratio | 8250 European |
| GCST90200736 | Adenosine 5'-diphosphate (ADP) to arginine ratio | 4568 European |
| GCST90200737 | Adenosine 5'-diphosphate (ADP) to 2'-deoxyuridine ratio | 4391 European |
| GCST90200738 | Adenosine 5'-monophosphate (AMP) to inosine 5'-monophosphate (IMP) ratio | 4601 European |
| GCST90200739 | Adenosine 5'-monophosphate (AMP) to proline ratio | 8190 European |
| GCST90200740 | Arachidonate (20:4n6) to oleate to vaccenate (18:1) ratio | 8171 European |
| GCST90200741 | Adenosine 5'-monophosphate (AMP) to alanine ratio | 8172 European |
| GCST90200742 | Aspartate to citrate ratio | 8191 European |
| GCST90200743 | Arginine to citrulline ratio | 8192 European |
| GCST90200744 | Arginine to glutamate ratio | 8194 European |
| GCST90200745 | Adenosine 5'-monophosphate (AMP) to phenylalanine ratio | 8189 European |
| GCST90200746 | Adenosine 5'-diphosphate (ADP) to gluconate ratio | 4540 European |
| GCST90200747 | Adenosine 5'-monophosphate (AMP) to phosphate ratio | 8184 European |
| GCST90200748 | Aspartate to glutamate ratio | 8190 European |
| GCST90200749 | Arachidonate (20:4n6) to pyruvate ratio | 8200 European |
| GCST90200750 | Aspartate to asparagine ratio | 8195 European |
| GCST90200751 | Cortisone to cortisol ratio | 8173 European |
| GCST90200752 | Succinate to acetoacetate ratio | 6829 European |
| GCST90200753 | Aspartate to citrulline ratio | 8139 European |
| GCST90200754 | Sphingosine to phosphate ratio | 8017 European |
| GCST90200755 | Inosine 5'-monophosphate (IMP) to phosphate ratio | 4605 European |
| GCST90200756 | Glycine to pyridoxal ratio | 8173 European |
| GCST90200757 | Glycine to alanine ratio | 8167 European |
| GCST90200758 | Glycine to serine ratio | 8206 European |
| GCST90200759 | Histidine to trans-urocanate ratio | 8109 European |
| GCST90200760 | Methionine to methionine sulfoxide ratio | 8214 European |
| GCST90200761 | Citrulline to dimethylarginine (SDMA + ADMA) ratio | 8243 European |
| GCST90200762 | Palmitate (16:0) to myristate (14:0) ratio | 8215 European |
| GCST90200763 | Phosphate to N-acetylneuraminate ratio | 8256 European |
| GCST90200764 | Phosphate to uridine ratio | 8186 European |
| GCST90200765 | Phosphate to alanine ratio | 8230 European |
| GCST90200766 | Phosphate to phosphoethanolamine ratio | 8205 European |
| GCST90200767 | Phosphate to glucose ratio | 8232 European |
| GCST90200768 | Phosphate to 2'-deoxyuridine ratio | 7846 European |
| GCST90200769 | Phosphate to mannose ratio | 8206 European |
| GCST90200770 | Phenylalanine to tyrosine ratio | 8205 European |
| GCST90200771 | Phosphate to fructose ratio | 8209 European |
| GCST90200772 | Phosphate to citrate ratio | 8238 European |
| GCST90200773 | Serine to alpha-tocopherol ratio | 8197 European |
| GCST90200774 | Uridine to pseudouridine ratio | 8254 European |
| GCST90200775 | Serine to pyruvate ratio | 8235 European |
| GCST90200776 | Glutamate to glutamine ratio | 8228 European |
| GCST90200777 | Uridine to cytidine ratio | 7705 European |
| GCST90200778 | Pyruvate to 3-methyl-2-oxobutyrate ratio | 8193 European |
| GCST90200779 | Pyruvate to N-acetylneuraminate ratio | 8194 European |
| GCST90200780 | Alanine to pyruvate ratio | 8250 European |
| GCST90200781 | Glucose to maltose ratio | 6308 European |
| GCST90200782 | Mannose to trans-4-hydroxyproline ratio | 8194 European |
| GCST90200783 | Mannose to S-methylcysteine ratio | 8182 European |
| GCST90200784 | Glutamate to cysteine ratio | 8199 European |
| GCST90200785 | Cysteine to 5-oxoproline ratio | 8203 European |
| GCST90200786 | Cysteine to alanine ratio | 8220 European |
| GCST90200787 | Glutamine to asparagine ratio | 8251 European |
| GCST90200788 | 5-oxoproline to citrate ratio | 8256 European |
| GCST90200789 | Alpha-tocopherol to sulfate ratio | 8201 European |
| GCST90200790 | Thyroxine to taurocholate ratio | 7615 European |
| GCST90200791 | Creatine to carnitine ratio | 8235 European |
| GCST90200792 | Carnitine to palmitoylcarnitine (C16) ratio | 8146 European |
| GCST90200793 | Glycolithocholate to glycolithocholate sulfate ratio | 7043 European |
| GCST90200794 | Oleoyl-linoleoyl-glycerol (18:1 to 18:2) [2] to linoleoyl-arachidonoyl-glycerol (18:2 to 20:4) [1] ratio | 7620 European |
| GCST90200795 | Oleoyl-linoleoyl-glycerol (18:1 to 18:2) [2] to linoleoyl-arachidonoyl-glycerol (18:2 to 20:4) [2] ratio | 7615 European |
| GCST90200796 | Spermidine to (N(1) + N(8))-acetylspermidine ratio | 7287 European |
| GCST90200797 | Spermidine to N-acetylputrescine ratio | 7299 European |
| GCST90200798 | Dopamine 4-sulfate to dopamine 3-O-sulfate ratio | 6840 European |
| GCST90200799 | Spermidine to adenosine 5'-diphosphate (ADP) ratio | 4195 European |
| GCST90200800 | Spermidine to histidine ratio | 7297 European |
| GCST90200801 | 5-methylthioadenosine (MTA) to phosphate ratio | 8261 European |
| GCST90200802 | Histidine to pyruvate ratio | 8241 European |
| GCST90200803 | Spermidine to phosphate ratio | 7382 European |
| GCST90200804 | Spermidine to pyruvate ratio | 7296 European |
| GCST90200805 | Spermidine to carnitine ratio | 7338 European |
| GCST90200806 | Spermidine to ergothioneine ratio | 7291 European |
| GCST90200807 | Carnitine to ergothioneine ratio | 8110 European |
| GCST90200808 | Spermidine to choline ratio | 7295 European |
| GCST90200809 | Spermidine to taurocholate ratio | 6777 European |
| GCST90200810 | Alpha-ketoglutarate to kynurenine ratio | 8272 European |
| GCST90200811 | Choline to taurocholate ratio | 7625 European |
| GCST90200812 | Glutamate to kynurenine ratio | 8184 European |
| GCST90200813 | Ornithine to glutamate ratio | 8202 European |
| GCST90200814 | Alpha-ketoglutarate to ornithine ratio | 8273 European |
| GCST90200815 | 3-hydroxyisobutyrate to adenosine 5'-diphosphate (ADP) ratio | 4488 European |
| GCST90200816 | Alpha-ketoglutarate to aspartate ratio | 8272 European |
| GCST90200817 | 3-hydroxyisobutyrate to phosphate ratio | 8170 European |
| GCST90200818 | Adenosine 5'-diphosphate (ADP) to glycerate ratio | 4544 European |
| GCST90200819 | 3-phosphoglycerate to glycerate ratio | 8101 European |
| GCST90200820 | Adenosine 5'-diphosphate (ADP) to valine ratio | 4608 European |
| GCST90200821 | Adenosine 5'-diphosphate (ADP) to glycine ratio | 4577 European |
| GCST90200822 | Phosphate to valine ratio | 8277 European |
| GCST90200823 | Adenosine 5'-diphosphate (ADP) to N-palmitoyl-sphingosine (d18:1 to 16:0) ratio | 4561 European |
| GCST90200824 | Adenosine 5'-diphosphate (ADP) to sulfate ratio | 4570 European |
| GCST90200825 | Phosphate to sulfate ratio | 8211 European |
| GCST90200826 | Glycine to phosphate ratio | 8271 European |
| GCST90200827 | Phosphate to N-palmitoyl-sphingosine (d18:1 to 16:0) ratio | 8223 European |
| GCST90200828 | Cholate to bilirubin (Z,Z) ratio | 7747 European |
| GCST90200829 | Bilirubin (Z,Z) to taurocholate ratio | 7620 European |
| GCST90200830 | Cholate to adenosine 3',5'-cyclic monophosphate (cAMP) ratio | 6359 European |
| GCST90200831 | Cholate to phosphate ratio | 7742 European |
| GCST90200832 | Adenosine 5'-diphosphate (ADP) to citrate ratio | 4567 European |
| GCST90200833 | Cholate to adenosine 5'-monophosphate (AMP) ratio | 7769 European |
| GCST90200834 | Adenosine 5'-diphosphate (ADP) to glycerol 3-phosphate ratio | 4588 European |
| GCST90200835 | Adenosine 5'-diphosphate (ADP) to glycerol ratio | 4575 European |
| GCST90200836 | Glycerol to glycerol 3-phosphate ratio | 8188 European |
| GCST90200837 | Adenosine 5'-diphosphate (ADP) to glutamate ratio | 4556 European |
| GCST90200838 | Phosphate to glutamate ratio | 8211 European |
| GCST90200839 | Adenosine 5'-diphosphate (ADP) to aspartate ratio | 4563 European |
| GCST90200840 | Adenosine 5'-diphosphate (ADP) to flavin adenine dinucleotide (FAD) ratio | 3441 European |
| GCST90200841 | Adenosine 5'-diphosphate (ADP) to oxalate (ethanedioate) ratio | 4556 European |
| GCST90200842 | Citrate to oxalate (ethanedioate) ratio | 8097 European |
| GCST90200843 | Adenosine 5'-monophosphate (AMP) to flavin adenine dinucleotide (FAD) ratio | 6188 European |
| GCST90200844 | Aspartate to phosphate ratio | 8297 European |
| GCST90200845 | Adenosine 5'-monophosphate (AMP) to citrate ratio | 8193 European |
| GCST90200846 | Phenylalanine to phosphate ratio | 8294 European |
| GCST90200847 | Phosphate to tryptophan ratio | 8206 European |
| GCST90200848 | Adenosine 5'-monophosphate (AMP) to glutamine ratio | 8196 European |
| GCST90200849 | Adenosine 5'-monophosphate (AMP) to threonine ratio | 8173 European |
| GCST90200850 | Adenosine 5'-monophosphate (AMP) to tryptophan ratio | 8180 European |
| GCST90200851 | Adenosine 5'-monophosphate (AMP) to glycine ratio | 8195 European |
| GCST90200852 | Adenosine 5'-monophosphate (AMP) to arginine ratio | 8185 European |
| GCST90200853 | Adenosine 5'-monophosphate (AMP) to tyrosine ratio | 8193 European |
| GCST90200854 | Arginine to phosphate ratio | 8297 European |
| GCST90200855 | Phosphate to threonine ratio | 8226 European |
| GCST90200856 | Phosphate to glutamine ratio | 8258 European |
| GCST90200857 | Adenosine 5'-monophosphate (AMP) to aspartate ratio | 8205 European |
| GCST90200858 | Phosphate to tyrosine ratio | 8226 European |
| GCST90200859 | Adenosine 5'-monophosphate (AMP) to asparagine ratio | 8188 European |
| GCST90200860 | Adenosine 5'-monophosphate (AMP) to serine ratio | 8194 European |
| GCST90200861 | Histidine to phosphate ratio | 8294 European |
| GCST90200862 | Adenosine 5'-monophosphate (AMP) to methionine ratio | 8194 European |
| GCST90200863 | Phosphate to serine ratio | 8226 European |
| GCST90200864 | Methionine to phosphate ratio | 8291 European |
| GCST90200865 | Adenosine 5'-monophosphate (AMP) to histidine ratio | 8180 European |
| GCST90200866 | phosphate to asparagine ratio | 8249 European |
| GCST90200867 | Adenosine 5'-monophosphate (AMP) to isoleucine ratio | 8200 European |
| GCST90200868 | Isoleucine to phosphate ratio | 8298 European |
| GCST90200869 | Adenosine 5'-monophosphate (AMP) to valine ratio | 8225 European |
| GCST90200870 | Adenosine 5'-monophosphate (AMP) to acetoacetate ratio | 6773 European |
| GCST90200871 | Adenosine 5'-monophosphate (AMP) to cysteine ratio | 8177 European |
| GCST90200872 | Phosphate to cysteine ratio | 8195 European |
| GCST90200873 | Cortisol to 4-cholesten-3-one ratio | 7027 European |
| GCST90200874 | Cysteinylglycine to taurine ratio | 8193 European |
| GCST90200875 | Cysteinylglycine to glutamate ratio | 8197 European |
| GCST90200876 | Cortisone to 4-cholesten-3-one ratio | 6971 European |
| GCST90200877 | Taurine to glutamate ratio | 8220 European |
| GCST90200878 | Glutarate (C5-DC) to caprylate (8:0) ratio | 7685 European |
| GCST90200879 | Adenosine 5'-monophosphate (AMP) to glutamate ratio | 8213 European |
| GCST90200880 | Glutarate (C5-DC) to salicylate ratio | 7684 European |
| GCST90200881 | Aspartate to N-acetylglucosamine to N-acetylgalactosamine ratio | 7899 European |
| GCST90200882 | Aspartate to mannose ratio | 8162 European |
| GCST90200883 | Phosphate to acetoacetate ratio | 6791 European |
| GCST90200884 | Mannose to N-acetylglucosamine to N-acetylgalactosamine ratio | 7907 European |
| GCST90200885 | Phenylpyruvate to citrate ratio | 8177 European |
| GCST90200886 | Phenylpyruvate to 4-hydroxyphenylpyruvate ratio | 7829 European |
| GCST90200887 | Cholesterol to cortisol ratio | 8211 European |
| GCST90200888 | Citrate to 4-hydroxyphenylpyruvate ratio | 7851 European |
| GCST90200889 | Cholesterol to taurocholate ratio | 7611 European |
| GCST90200890 | Cortisol to taurocholate ratio | 7610 European |
| GCST90200891 | Salicylate to caprylate (8:0) ratio | 8068 European |
| GCST90200892 | Hypotaurine to taurine ratio | 8157 European |
| GCST90200893 | Taurine to cysteine ratio | 8186 European |
| GCST90200894 | Inosine to theophylline ratio | 4662 European |
| GCST90200895 | Hypotaurine to cysteine ratio | 8134 European |
| GCST90200896 | Inosine to EDTA ratio | 4926 European |
| GCST90200897 | Theophylline to EDTA ratio | 7867 European |
| GCST90200898 | Citrulline to phosphate ratio | 8294 European |
| GCST90200899 | Citrulline to ornithine ratio | 8225 European |
| GCST90200900 | Phosphate to oleoyl-linoleoyl-glycerol (18:1 to 18:2) [2] ratio | 7992 European |
| GCST90200901 | Ornithine to phosphate ratio | 8295 European |
| GCST90200902 | Phosphate to linoleoyl-arachidonoyl-glycerol (18:2 to 20:4) [2] ratio | 7649 European |
| GCST90200903 | Phosphate to glycerol ratio | 8179 European |
| GCST90200904 | Phosphate to linoleoyl-arachidonoyl-glycerol (18:2 to 20:4) [1] ratio | 7623 European |
| GCST90200905 | Glucose to glycerol ratio | 8160 European |
| GCST90200906 | 2'-deoxyuridine to cytidine ratio | 7410 European |
| GCST90200907 | Retinol (Vitamin A) to linoleoyl-arachidonoyl-glycerol (18:2 to 20:4) [1] ratio | 7617 European |
| GCST90200908 | Retinol (Vitamin A) to linoleoyl-arachidonoyl-glycerol (18:2 to 20:4) [2] ratio | 7668 European |
| GCST90200909 | Uridine to 2'-deoxyuridine ratio | 7873 European |
| GCST90200910 | Retinol (Vitamin A) to oleoyl-linoleoyl-glycerol (18:1 to 18:2) [2] ratio | 7981 European |
| GCST90200911 | Tryptophan to pyruvate ratio | 8224 European |
| GCST90200912 | tryptophan to tyrosine ratio | 8226 European |
| GCST90200913 | Glucose to sucrose ratio | 7695 European |
| GCST90200914 | Tyrosine to pyruvate ratio | 8220 European |
| GCST90200915 | Glucose to fructose ratio | 8214 European |
| GCST90200916 | Fructose to sucrose ratio | 7719 European |
| GCST90200917 | Alpha-ketobutyrate to 3-methyl-2-oxobutyrate ratio | 8133 European |
| GCST90200918 | N-palmitoyl-sphingosine (d18:1 to 16:0) to N-palmitoyl-sphinganine (d18:0 to 16:0) ratio | 8155 European |
| GCST90200919 | Caffeine to theophylline ratio | 7756 European |
| GCST90200920 | Mannose to glycerol ratio | 8168 European |
| GCST90200921 | Mannose to mannitol to sorbitol ratio | 8208 European |
| GCST90200922 | Alpha-ketobutyrate to pyruvate ratio | 8129 European |
| GCST90200923 | Glycerol to mannitol to sorbitol ratio | 8153 European |
| GCST90200924 | Alpha-tocopherol to glycerol ratio | 8158 European |
| GCST90200925 | Caffeine to theobromine ratio | 7952 European |
| GCST90200926 | N-stearoyl-sphingosine (d18:1 to 18:0) to N-palmitoyl-sphinganine (d18:0 to 16:0) ratio | 8159 European |
| GCST90200927 | Theophylline to theobromine ratio | 7858 European |
| GCST90200928 | N-palmitoyl-sphingosine (d18:1 to 16:0) to N-stearoyl-sphingosine (d18:1 to 18:0) ratio | 8191 European |
| GCST90200929 | Glycerol to carnitine ratio | 8235 European |
| GCST90200930 | Carnitine to acetylcarnitine (C2) ratio | 8206 European |
| GCST90200931 | Glycerol to sulfate ratio | 8176 European |
| GCST90200932 | Glycerol to palmitoylcarnitine (C16) ratio | 8165 European |
| GCST90200933 | Alpha-ketoglutarate to proline ratio | 8273 European |
| GCST90200934 | Acetylcarnitine (C2) to propionylcarnitine (C3) ratio | 8200 European |
| GCST90200935 | Carnitine to propionylcarnitine (C3) ratio | 8185 European |
| GCST90200936 | Succinate to proline ratio | 8209 European |
| GCST90200937 | Alpha-ketoglutarate to trans-4-hydroxyproline ratio | 8272 European |
| GCST90200938 | Alpha-ketoglutarate to alanine ratio | 8272 European |
| GCST90200939 | Proline to trans-4-hydroxyproline ratio | 8194 European |
| GCST90200940 | Adenosine 5'-diphosphate (ADP) to EDTA ratio | 4563 European |
| GCST90200941 | Succinate to trans-4-hydroxyproline ratio | 8198 European |
| GCST90200942 | Glutamate to pyruvate ratio | 8188 European |
| GCST90200943 | Phosphate to EDTA ratio | 8225 European |
| GCST90200944 | Alpha-ketoglutarate to pyruvate ratio | 8272 European |
| GCST90200945 | Adenosine 5'-monophosphate (AMP) to EDTA ratio | 8179 European |
| GCST90200946 | Glutamate to alanine ratio | 8172 European |
| GCST90200947 | Adenosine 5'-diphosphate (ADP) to glucose ratio | 4581 European |
| GCST90200948 | Adenosine 5'-diphosphate (ADP) to mannitol to sorbitol ratio | 4566 European |
| GCST90200949 | Adenosine 5'-diphosphate (ADP) to N-acetylneuraminate ratio | 4553 European |
| GCST90200950 | Glucose to mannitol to sorbitol ratio | 8254 European |
| GCST90200951 | Mannose to fructose ratio | 8202 European |
| GCST90200952 | Adenosine 5'-diphosphate (ADP) to cytidine ratio | 4350 European |
| GCST90200953 | Glucose-to-mannose ratio | 8191 European |
| GCST90200954 | Cytidine to N-acetylneuraminate ratio | 7683 European |
| GCST90200955 | Adenosine 5'-diphosphate (ADP) to choline phosphate ratio | 4560 European |
| GCST90200956 | Cytidine to N-acetylglucosamine to N-acetylgalactosamine ratio | 7427 European |
| GCST90200957 | N-acetylneuraminate to N-acetylglucosamine to N-acetylgalactosamine ratio | 7988 European |
| GCST90200958 | Adenosine 5'-diphosphate (ADP) to choline ratio | 4560 European |
| GCST90200959 | Choline phosphate to choline ratio | 8196 European |
| GCST90200960 | Choline phosphate to phosphoethanolamine ratio | 8213 European |
| GCST90200961 | Adenosine 5'-diphosphate (ADP) to ornithine ratio | 4566 European |
| GCST90200962 | Adenosine 5'-diphosphate (ADP) to glutamine ratio | 4569 European |
| GCST90200963 | Inosine 5'-monophosphate (IMP) to urate ratio | 4598 European |
| GCST90200964 | Adenosine 5'-diphosphate (ADP) to 5-oxoproline ratio | 4570 European |
| GCST90200965 | Glutamate to 5-oxoproline ratio | 8171 European |
| GCST90200966 | Adenosine 5'-diphosphate (ADP) to uridine ratio | 4565 European |
| GCST90200967 | Phosphate to urate ratio | 8204 European |
| GCST90200968 | Phosphate to 5-oxoproline ratio | 8217 European |
| GCST90200969 | Adenosine 5'-monophosphate (AMP) to N-palmitoyl-sphingosine (d18:1 to 16:0) ratio | 8188 European |
| GCST90200970 | Adenosine 5'-monophosphate (AMP) to leucine ratio | 8201 European |
| GCST90200971 | Leucine to phosphate ratio | 8298 European |
| GCST90200972 | Leucine to N-palmitoyl-sphingosine (d18:1 to 16:0) ratio | 8198 European |
| GCST90200973 | Proline to glutamate ratio | 8185 European |
| GCST90200974 | Phosphate to proline ratio | 8241 European |
| GCST90200975 | Arachidonate (20:4n6) to caffeine ratio | 8000 European |
| GCST90200976 | Adenosine 5'-monophosphate (AMP) to urate ratio | 8181 European |
| GCST90200977 | Arachidonate (20:4n6) to paraxanthine ratio | 7776 European |
| GCST90200978 | Caffeine to paraxanthine ratio | 7873 European |
| GCST90200979 | Arachidonate (20:4n6) to linoleate (18:2n6) ratio | 8162 European |
| GCST90200980 | Phosphoethanolamine to choline ratio | 8204 European |
| GCST90200981 | Caffeine to linoleate (18:2n6) ratio | 7939 European |
| GCST90200982 | Paraxanthine to linoleate (18:2n6) ratio | 7785 European |
| GCST90200983 | Cholesterol to linoleoyl-arachidonoyl-glycerol (18:2 to 20:4) [1] ratio | 7624 European |
| GCST90200984 | Cholesterol to benzoate ratio | 7387 European |
| GCST90200985 | Cholesterol to oleoyl-linoleoyl-glycerol (18:1 to 18:2) [2] ratio | 7981 European |
| GCST90200986 | Cholesterol to linoleoyl-arachidonoyl-glycerol (18:2 to 20:4) [2] ratio | 7655 European |
| GCST90200987 | Benzoate to oleoyl-linoleoyl-glycerol (18:1 to 18:2) [2] ratio | 7162 European |
| GCST90200988 | Benzoate to linoleoyl-arachidonoyl-glycerol (18:2 to 20:4) [1] ratio | 6813 European |
| GCST90200989 | Histidine to asparagine ratio | 8225 European |
| GCST90200990 | Benzoate to linoleoyl-arachidonoyl-glycerol (18:2 to 20:4) [2] ratio | 6865 European |
| GCST90200991 | Histidine to alanine ratio | 8233 European |
| GCST90200992 | Alanine to asparagine ratio | 8248 European |
| GCST90200993 | Salicylate to oxalate (ethanedioate) ratio | 8107 European |
| GCST90200994 | Glutamine to alanine ratio | 8222 European |
| GCST90200995 | Salicylate to taurocholate ratio | 7614 European |
| GCST90200996 | Citrate to taurocholate ratio | 7618 European |
| GCST90200997 | Histidine to glutamine ratio | 8223 European |
| GCST90200998 | Salicylate to citrate ratio | 8147 European |
| GCST90200999 | Taurocholate to oxalate (ethanedioate) ratio | 7755 European |
| GCST90201000 | Serine to threonine ratio | 8237 European |
| GCST90201001 | Serine to alpha-ketobutyrate ratio | 8100 European |
| GCST90201002 | Maltose to sucrose ratio | 5984 European |
| GCST90201003 | Glucose to N-palmitoyl-sphingosine (d18:1 to 16:0) ratio | 8173 European |
| GCST90201004 | Glucose to N-stearoyl-sphingosine (d18:1 to 18:0) ratio | 8179 European |
| GCST90201005 | Fructose to maltose ratio | 6307 European |
| GCST90201006 | Alpha-ketobutyrate to 3-methyl-2-oxovalerate ratio | 8136 European |
| GCST90201007 | Glucose to N-palmitoyl-sphinganine (d18:0 to 16:0) ratio | 8119 European |
| GCST90201008 | Threonine to alpha-ketobutyrate ratio | 8099 European |
| GCST90201009 | Threonine to pyruvate ratio | 8220 European |
| GCST90201010 | Alpha-ketobutyrate to 4-methyl-2-oxopentanoate ratio | 8142 European |
| GCST90201011 | 3-methyl-2-oxovalerate to 4-methyl-2-oxopentanoate ratio | 8233 European |
| GCST90201012 | 3-methyl-2-oxovalerate to 3-methyl-2-oxobutyrate ratio | 8200 European |
| GCST90201013 | Androsterone glucuronide to etiocholanolone glucuronide ratio | 7555 European |
| GCST90201014 | Glucuronate to androsterone glucuronide ratio | 7804 European |
| GCST90201015 | Bilirubin (Z,Z) to androsterone glucuronide ratio | 7888 European |
| GCST90201016 | Bilirubin (Z,Z) to etiocholanolone glucuronide ratio | 7707 European |
| GCST90201017 | 4-methyl-2-oxopentanoate to 3-methyl-2-oxobutyrate ratio | 8224 European |
| GCST90201018 | Bilirubin (Z,Z) to glucuronate ratio | 8103 European |
| GCST90201019 | Glucuronate to etiocholanolone glucuronide ratio | 7631 European |
| GCST90201020 | Paraxanthine to 5-acetylamino-6-formylamino-3-methyluracil ratio | 7388 European |

**Table S3.** Causal effects of the blood metabolites on chronic periodontitis.

| **exposure** | **outcome** | **MR method** | **nSNP** | **beta** | **se** | **p-value** | **OR** | **or_lci95** | **or_uci95** | **fdr_p-value** | **fdr_significant** |
| --- | --- | --- | --- | --- | --- | --- | --- | --- | --- | --- | --- |
| Maltotriose levels | chronic periodontitis | MR Egger | 23 | 0.079384986 | 0.094338026 | 0.409551276 | 1.082621036 | 0.899858684 | 1.302502636 |  |  |
| Maltotriose levels | chronic periodontitis | Weighted median | 23 | 0.100452096 | 0.061058833 | 0.099934976 | 1.105670674 | 0.980960371 | 1.246235502 |  |  |
| Maltotriose levels | chronic periodontitis | Inverse variance weighted | 23 | 0.151548704 | 0.045202503 | 0.000800385 | 1.163634974 | 1.064975504 | 1.27143427 | 0.207139638 | FALSE |
| Maltotriose levels | chronic periodontitis | Simple mode | 23 | 0.103747883 | 0.10567635 | 0.336896474 | 1.109320741 | 0.901786247 | 1.364616626 |  |  |
| Maltotriose levels | chronic periodontitis | Weighted mode | 23 | 0.097913118 | 0.089808571 | 0.287404211 | 1.102866962 | 0.924861131 | 1.315133153 |  |  |
| 4-guanidinobutanoate levels | chronic periodontitis | MR Egger | 48 | -0.006688421 | 0.057302526 | 0.907589087 | 0.993333897 | 0.887806583 | 1.111404498 |  |  |
| 4-guanidinobutanoate levels | chronic periodontitis | Weighted median | 48 | -0.045144407 | 0.04078793 | 0.268376951 | 0.955859439 | 0.882418584 | 1.035412539 |  |  |
| 4-guanidinobutanoate levels | chronic periodontitis | Inverse variance weighted | 48 | -0.069977783 | 0.030322645 | 0.021011761 | 0.932414535 | 0.878613514 | 0.989510008 | 0.763226997 | FALSE |
| 4-guanidinobutanoate levels | chronic periodontitis | Simple mode | 48 | -0.097809695 | 0.078688481 | 0.220033576 | 0.90682146 | 0.777214334 | 1.058041681 |  |  |
| 4-guanidinobutanoate levels | chronic periodontitis | Weighted mode | 48 | -0.041514276 | 0.039463234 | 0.298190414 | 0.95933564 | 0.887930136 | 1.036483427 |  |  |
| Ribitol levels | chronic periodontitis | MR Egger | 36 | -0.079471015 | 0.038098655 | 0.044560244 | 0.92360479 | 0.857148258 | 0.995213838 |  |  |
| Ribitol levels | chronic periodontitis | Weighted median | 36 | -0.105088756 | 0.037587631 | 0.005176607 | 0.900244617 | 0.83630616 | 0.969071387 |  |  |
| Ribitol levels | chronic periodontitis | Inverse variance weighted | 36 | -0.079048414 | 0.027014246 | 0.003431577 | 0.923995189 | 0.876344207 | 0.974237181 | 0.339097768 | FALSE |
| Ribitol levels | chronic periodontitis | Simple mode | 36 | -0.079897165 | 0.069478035 | 0.257956259 | 0.92321128 | 0.805675589 | 1.057893622 |  |  |
| Ribitol levels | chronic periodontitis | Weighted mode | 36 | -0.106014896 | 0.037569044 | 0.007818248 | 0.89941125 | 0.835562422 | 0.968139036 |  |  |
| Indoleacetate levels | chronic periodontitis | MR Egger | 27 | 0.137550854 | 0.118380348 | 0.256233171 | 1.147460058 | 0.90985082 | 1.447121391 |  |  |
| Indoleacetate levels | chronic periodontitis | Weighted median | 27 | 0.147533773 | 0.072354317 | 0.041445447 | 1.158972426 | 1.005735699 | 1.335556732 |  |  |
| Indoleacetate levels | chronic periodontitis | Inverse variance weighted | 27 | 0.103592384 | 0.052727829 | 0.049453324 | 1.109148256 | 1.000245869 | 1.229907457 | 0.852187617 | FALSE |
| Indoleacetate levels | chronic periodontitis | Simple mode | 27 | 0.123475693 | 0.124071784 | 0.32881355 | 1.131422503 | 0.887182113 | 1.442902039 |  |  |
| Indoleacetate levels | chronic periodontitis | Weighted mode | 27 | 0.168860211 | 0.101007828 | 0.106567148 | 1.183954623 | 0.971304629 | 1.443160577 |  |  |
| 1-methylhistidine levels | chronic periodontitis | MR Egger | 32 | -0.126015872 | 0.125981735 | 0.325179656 | 0.881600854 | 0.688706504 | 1.128521455 |  |  |
| 1-methylhistidine levels | chronic periodontitis | Weighted median | 32 | -0.126100739 | 0.069900479 | 0.071230692 | 0.881526039 | 0.768660663 | 1.010963868 |  |  |
| 1-methylhistidine levels | chronic periodontitis | Inverse variance weighted | 32 | -0.100669504 | 0.048185249 | 0.036688078 | 0.904231829 | 0.822742019 | 0.993792928 | 0.852187617 | FALSE |
| 1-methylhistidine levels | chronic periodontitis | Simple mode | 32 | -0.1627442 | 0.14071169 | 0.256279717 | 0.849808542 | 0.644977973 | 1.119688715 |  |  |
| 1-methylhistidine levels | chronic periodontitis | Weighted mode | 32 | -0.178148784 | 0.139256048 | 0.210288114 | 0.836817909 | 0.63693311 | 1.099431325 |  |  |
| Butyrylglycine levels | chronic periodontitis | MR Egger | 47 | -0.070942807 | 0.042462254 | 0.101717587 | 0.931515167 | 0.857127304 | 1.01235896 |  |  |
| Butyrylglycine levels | chronic periodontitis | Weighted median | 47 | -0.086647623 | 0.036224259 | 0.016757856 | 0.917000169 | 0.854151099 | 0.984473719 |  |  |
| Butyrylglycine levels | chronic periodontitis | Inverse variance weighted | 47 | -0.086533987 | 0.022009803 | 8.44E-05 | 0.917104378 | 0.878382439 | 0.957533306 | 0.1092136 | TRUE |
| Butyrylglycine levels | chronic periodontitis | Simple mode | 47 | -0.060117437 | 0.060290998 | 0.323922161 | 0.941653942 | 0.836701591 | 1.059771077 |  |  |
| Butyrylglycine levels | chronic periodontitis | Weighted mode | 47 | -0.07659379 | 0.032585774 | 0.023090234 | 0.926266036 | 0.868956765 | 0.987354957 |  |  |
| N-acetylhistidine levels | chronic periodontitis | MR Egger | 46 | 0.094969663 | 0.041832659 | 0.028144329 | 1.099625495 | 1.013062227 | 1.193585346 |  |  |
| N-acetylhistidine levels | chronic periodontitis | Weighted median | 46 | 0.094676527 | 0.037056295 | 0.010620679 | 1.099303203 | 1.022291002 | 1.182116961 |  |  |
| N-acetylhistidine levels | chronic periodontitis | Inverse variance weighted | 46 | 0.073436343 | 0.025677595 | 0.004237232 | 1.076200027 | 1.023377321 | 1.131749232 | 0.341573895 | FALSE |
| N-acetylhistidine levels | chronic periodontitis | Simple mode | 46 | 0.040084423 | 0.069654686 | 0.567837824 | 1.040898646 | 0.908065505 | 1.193162812 |  |  |
| N-acetylhistidine levels | chronic periodontitis | Weighted mode | 46 | 0.088961932 | 0.036931268 | 0.020158611 | 1.093039046 | 1.016714801 | 1.175092911 |  |  |
| 5-methyluridine (ribothymidine) levels | chronic periodontitis | MR Egger | 46 | 0.046011189 | 0.042542572 | 0.285352294 | 1.047086127 | 0.963317471 | 1.13813918 |  |  |
| 5-methyluridine (ribothymidine) levels | chronic periodontitis | Weighted median | 46 | 0.03985145 | 0.041506596 | 0.336993162 | 1.040656173 | 0.959347918 | 1.128855601 |  |  |
| 5-methyluridine (ribothymidine) levels | chronic periodontitis | Inverse variance weighted | 46 | 0.060888748 | 0.026073553 | 0.019529398 | 1.062780671 | 1.009832609 | 1.118504934 | 0.761660046 | FALSE |
| 5-methyluridine (ribothymidine) levels | chronic periodontitis | Simple mode | 46 | 0.047565463 | 0.078255835 | 0.546362311 | 1.048714851 | 0.899590182 | 1.22255985 |  |  |
| 5-methyluridine (ribothymidine) levels | chronic periodontitis | Weighted mode | 46 | 0.047565463 | 0.044021825 | 0.285677755 | 1.048714851 | 0.962022621 | 1.143219311 |  |  |
| 1-arachidonoyl-GPE (20:4n6) levels | chronic periodontitis | MR Egger | 51 | 0.063292172 | 0.061639378 | 0.309550454 | 1.065338056 | 0.944102057 | 1.202142465 |  |  |
| 1-arachidonoyl-GPE (20:4n6) levels | chronic periodontitis | Weighted median | 51 | 0.061089215 | 0.041999807 | 0.145804463 | 1.062993745 | 0.978993372 | 1.154201584 |  |  |
| 1-arachidonoyl-GPE (20:4n6) levels | chronic periodontitis | Inverse variance weighted | 51 | 0.066510345 | 0.030133809 | 0.027302406 | 1.06877202 | 1.007475884 | 1.133797493 | 0.852087914 | FALSE |
| 1-arachidonoyl-GPE (20:4n6) levels | chronic periodontitis | Simple mode | 51 | 0.034581413 | 0.078151398 | 0.660041131 | 1.035186303 | 0.888167146 | 1.206541681 |  |  |
| 1-arachidonoyl-GPE (20:4n6) levels | chronic periodontitis | Weighted mode | 51 | 0.069678038 | 0.040296745 | 0.089957513 | 1.07216293 | 0.990739563 | 1.160278031 |  |  |
| Docosapentaenoate (n6 DPA; 22:5n6) levels | chronic periodontitis | MR Egger | 19 | 0.21596666 | 0.141352188 | 0.144938657 | 1.241061002 | 0.940744564 | 1.637248269 |  |  |
| Docosapentaenoate (n6 DPA; 22:5n6) levels | chronic periodontitis | Weighted median | 19 | 0.081926112 | 0.084387269 | 0.331630466 | 1.08537561 | 0.919915983 | 1.280595442 |  |  |
| Docosapentaenoate (n6 DPA; 22:5n6) levels | chronic periodontitis | Inverse variance weighted | 19 | 0.121901813 | 0.059735663 | 0.041281912 | 1.12964318 | 1.004831548 | 1.269957851 | 0.852187617 | FALSE |
| Docosapentaenoate (n6 DPA; 22:5n6) levels | chronic periodontitis | Simple mode | 19 | 0.114686983 | 0.143667833 | 0.435115052 | 1.121522327 | 0.846282568 | 1.486279379 |  |  |
| Docosapentaenoate (n6 DPA; 22:5n6) levels | chronic periodontitis | Weighted mode | 19 | 0.076713886 | 0.117168148 | 0.520918925 | 1.079733105 | 0.858184915 | 1.358475962 |  |  |
| Catechol sulfate levels | chronic periodontitis | MR Egger | 26 | -0.258415277 | 0.106360823 | 0.022967663 | 0.772274457 | 0.626953759 | 0.951278829 |  |  |
| Catechol sulfate levels | chronic periodontitis | Weighted median | 26 | -0.134204746 | 0.081056225 | 0.097782701 | 0.874411014 | 0.745966231 | 1.024972163 |  |  |
| Catechol sulfate levels | chronic periodontitis | Inverse variance weighted | 26 | -0.120435931 | 0.060659277 | 0.047094482 | 0.886533885 | 0.787156563 | 0.998457442 | 0.852187617 | FALSE |
| Catechol sulfate levels | chronic periodontitis | Simple mode | 26 | -0.425764048 | 0.196568716 | 0.040051141 | 0.653270464 | 0.444396218 | 0.960319377 |  |  |
| Catechol sulfate levels | chronic periodontitis | Weighted mode | 26 | -0.324796754 | 0.114038437 | 0.008669836 | 0.72267422 | 0.577924472 | 0.903678687 |  |  |
| Mannitol/sorbitol levels | chronic periodontitis | MR Egger | 25 | 0.113280751 | 0.103088963 | 0.283197461 | 1.119946315 | 0.915052703 | 1.370718586 |  |  |
| Mannitol/sorbitol levels | chronic periodontitis | Weighted median | 25 | 0.04775423 | 0.078037255 | 0.540576509 | 1.048912832 | 0.900145564 | 1.222266901 |  |  |
| Mannitol/sorbitol levels | chronic periodontitis | Inverse variance weighted | 25 | 0.127782395 | 0.05938746 | 0.031423117 | 1.13630571 | 1.011448006 | 1.276576413 | 0.852187617 | FALSE |
| Mannitol/sorbitol levels | chronic periodontitis | Simple mode | 25 | 0.069573867 | 0.13905139 | 0.621388428 | 1.072051248 | 0.816305295 | 1.407921625 |  |  |
| Mannitol/sorbitol levels | chronic periodontitis | Weighted mode | 25 | 0.034458818 | 0.090454783 | 0.70659115 | 1.035059402 | 0.866899213 | 1.235839126 |  |  |
| 1-palmitoyl-2-linoleoyl-GPE (16:0/18:2) levels | chronic periodontitis | MR Egger | 54 | -0.028369877 | 0.047530055 | 0.553175622 | 0.972028769 | 0.885565562 | 1.066933911 |  |  |
| 1-palmitoyl-2-linoleoyl-GPE (16:0/18:2) levels | chronic periodontitis | Weighted median | 54 | -0.036236156 | 0.037212411 | 0.330173074 | 0.964412515 | 0.896575761 | 1.037381937 |  |  |
| 1-palmitoyl-2-linoleoyl-GPE (16:0/18:2) levels | chronic periodontitis | Inverse variance weighted | 54 | -0.052004457 | 0.024734172 | 0.035506456 | 0.949324635 | 0.904400094 | 0.996480727 | 0.852187617 | FALSE |
| 1-palmitoyl-2-linoleoyl-GPE (16:0/18:2) levels | chronic periodontitis | Simple mode | 54 | -0.002864797 | 0.065820424 | 0.965447046 | 0.997139302 | 0.876452448 | 1.134444647 |  |  |
| 1-palmitoyl-2-linoleoyl-GPE (16:0/18:2) levels | chronic periodontitis | Weighted mode | 54 | -0.037812549 | 0.037625788 | 0.319480667 | 0.962893419 | 0.894438534 | 1.036587425 |  |  |
| Succinylcarnitine levels | chronic periodontitis | MR Egger | 45 | -0.112764369 | 0.060646579 | 0.06982595 | 0.893361139 | 0.793238248 | 1.006121588 |  |  |
| Succinylcarnitine levels | chronic periodontitis | Weighted median | 45 | -0.115497454 | 0.047937547 | 0.015981764 | 0.890922841 | 0.811026097 | 0.978690465 |  |  |
| Succinylcarnitine levels | chronic periodontitis | Inverse variance weighted | 45 | -0.089500949 | 0.030306093 | 0.003144647 | 0.914387397 | 0.861654507 | 0.970347518 | 0.339097768 | FALSE |
| Succinylcarnitine levels | chronic periodontitis | Simple mode | 45 | -0.088253061 | 0.087428334 | 0.31828605 | 0.915529163 | 0.771350352 | 1.086657503 |  |  |
| Succinylcarnitine levels | chronic periodontitis | Weighted mode | 45 | -0.11766753 | 0.050640658 | 0.024831225 | 0.888991567 | 0.804991775 | 0.981756622 |  |  |
| Andro steroid monosulfate C19H28O6S (1) levels | chronic periodontitis | MR Egger | 52 | 0.064303161 | 0.037864976 | 0.095681615 | 1.066415646 | 0.990136773 | 1.148570945 |  |  |
| Andro steroid monosulfate C19H28O6S (1) levels | chronic periodontitis | Weighted median | 52 | 0.045244628 | 0.039995862 | 0.257957161 | 1.046283778 | 0.967396088 | 1.131604478 |  |  |
| Andro steroid monosulfate C19H28O6S (1) levels | chronic periodontitis | Inverse variance weighted | 52 | 0.055715037 | 0.025467099 | 0.02868956 | 1.05729635 | 1.005816373 | 1.111411191 | 0.852087914 | FALSE |
| Andro steroid monosulfate C19H28O6S (1) levels | chronic periodontitis | Simple mode | 52 | 0.05496589 | 0.075509599 | 0.469983931 | 1.056504576 | 0.911163501 | 1.225029228 |  |  |
| Andro steroid monosulfate C19H28O6S (1) levels | chronic periodontitis | Weighted mode | 52 | 0.05496589 | 0.036286317 | 0.13600103 | 1.056504576 | 0.983974506 | 1.134380935 |  |  |
| Gamma-CEHC levels | chronic periodontitis | MR Egger | 40 | 0.107494317 | 0.080147204 | 0.187813661 | 1.113484533 | 0.95161551 | 1.302887345 |  |  |
| Gamma-CEHC levels | chronic periodontitis | Weighted median | 40 | 0.097327908 | 0.056947967 | 0.087438487 | 1.102221742 | 0.985811511 | 1.232378355 |  |  |
| Gamma-CEHC levels | chronic periodontitis | Inverse variance weighted | 40 | 0.074990364 | 0.037582733 | 0.046005714 | 1.077873764 | 1.001329089 | 1.16026975 | 0.852187617 | FALSE |
| Gamma-CEHC levels | chronic periodontitis | Simple mode | 40 | 0.081445945 | 0.092082318 | 0.381852068 | 1.084854574 | 0.905710646 | 1.299432056 |  |  |
| Gamma-CEHC levels | chronic periodontitis | Weighted mode | 40 | 0.097905039 | 0.065202292 | 0.141265145 | 1.102858052 | 0.970550879 | 1.253201568 |  |  |
| N-acetyl-beta-alanine levels | chronic periodontitis | MR Egger | 35 | -0.048138456 | 0.037824194 | 0.212022244 | 0.953001829 | 0.884905981 | 1.026337833 |  |  |
| N-acetyl-beta-alanine levels | chronic periodontitis | Weighted median | 35 | -0.034010245 | 0.035623439 | 0.339721643 | 0.966561602 | 0.901376549 | 1.036460657 |  |  |
| N-acetyl-beta-alanine levels | chronic periodontitis | Inverse variance weighted | 35 | -0.085545746 | 0.0283326 | 0.002533262 | 0.918011147 | 0.868421884 | 0.970432092 | 0.339097768 | FALSE |
| N-acetyl-beta-alanine levels | chronic periodontitis | Simple mode | 35 | -0.280991932 | 0.094622914 | 0.005435575 | 0.755034427 | 0.627223186 | 0.908890167 |  |  |
| N-acetyl-beta-alanine levels | chronic periodontitis | Weighted mode | 35 | -0.042201579 | 0.033531898 | 0.216766532 | 0.958676512 | 0.89769573 | 1.02379974 |  |  |
| Alliin levels | chronic periodontitis | MR Egger | 24 | -0.15342223 | 0.06711193 | 0.032243675 | 0.85776747 | 0.752043122 | 0.978354845 |  |  |
| Alliin levels | chronic periodontitis | Weighted median | 24 | -0.098049392 | 0.054331854 | 0.071131162 | 0.906604124 | 0.815022283 | 1.008476767 |  |  |
| Alliin levels | chronic periodontitis | Inverse variance weighted | 24 | -0.09962103 | 0.036444012 | 0.006265822 | 0.905180389 | 0.842778343 | 0.972202885 | 0.421304208 | FALSE |
| Alliin levels | chronic periodontitis | Simple mode | 24 | -0.155190902 | 0.093228709 | 0.109553368 | 0.856251702 | 0.713252936 | 1.027920026 |  |  |
| Alliin levels | chronic periodontitis | Weighted mode | 24 | -0.101968376 | 0.0592531 | 0.098696857 | 0.90305811 | 0.804041454 | 1.014268538 |  |  |
| 3b-hydroxy-5-cholenoic acid levels | chronic periodontitis | MR Egger | 27 | 0.082938143 | 0.103215177 | 0.429233668 | 1.0864746 | 0.887485049 | 1.330081061 |  |  |
| 3b-hydroxy-5-cholenoic acid levels | chronic periodontitis | Weighted median | 27 | 0.150469385 | 0.069003018 | 0.029211508 | 1.162379718 | 1.015339938 | 1.330713546 |  |  |
| 3b-hydroxy-5-cholenoic acid levels | chronic periodontitis | Inverse variance weighted | 27 | 0.120395483 | 0.050344516 | 0.016782797 | 1.127942845 | 1.021957832 | 1.244919332 | 0.711482985 | FALSE |
| 3b-hydroxy-5-cholenoic acid levels | chronic periodontitis | Simple mode | 27 | 0.050492693 | 0.115659434 | 0.666031953 | 1.051789178 | 0.838450446 | 1.319410681 |  |  |
| 3b-hydroxy-5-cholenoic acid levels | chronic periodontitis | Weighted mode | 27 | 0.11536187 | 0.091761822 | 0.219861945 | 1.122279484 | 0.93754427 | 1.343415218 |  |  |
| Glycohyocholate levels | chronic periodontitis | MR Egger | 17 | 0.176497911 | 0.141395581 | 0.231070579 | 1.193031934 | 0.904260834 | 1.574020619 |  |  |
| Glycohyocholate levels | chronic periodontitis | Weighted median | 17 | 0.096498046 | 0.09446902 | 0.307027925 | 1.101307429 | 0.915155546 | 1.325324486 |  |  |
| Glycohyocholate levels | chronic periodontitis | Inverse variance weighted | 17 | 0.183538538 | 0.066259598 | 0.005605772 | 1.201461267 | 1.05513606 | 1.368078706 | 0.421304208 | FALSE |
| Glycohyocholate levels | chronic periodontitis | Simple mode | 17 | 0.070286478 | 0.167284748 | 0.679956313 | 1.072815475 | 0.772910802 | 1.489089091 |  |  |
| Glycohyocholate levels | chronic periodontitis | Weighted mode | 17 | 0.060033819 | 0.164995234 | 0.720731949 | 1.061872458 | 0.76846763 | 1.467300733 |  |  |
| 9-hydroxystearate levels | chronic periodontitis | MR Egger | 24 | -0.007968314 | 0.169469037 | 0.962922077 | 0.992063349 | 0.711679488 | 1.382911416 |  |  |
| 9-hydroxystearate levels | chronic periodontitis | Weighted median | 24 | -0.143846371 | 0.088416306 | 0.103753815 | 0.866020784 | 0.728227098 | 1.029887516 |  |  |
| 9-hydroxystearate levels | chronic periodontitis | Inverse variance weighted | 24 | -0.147781649 | 0.066869304 | 0.027104657 | 0.862619448 | 0.756656808 | 0.983421154 | 0.776834821 | FALSE |
| 9-hydroxystearate levels | chronic periodontitis | Simple mode | 24 | -0.107084652 | 0.175362639 | 0.547420876 | 0.898449614 | 0.637121169 | 1.266967335 |  |  |
| 9-hydroxystearate levels | chronic periodontitis | Weighted mode | 24 | -0.123198629 | 0.15887522 | 0.44597843 | 0.88408804 | 0.647527471 | 1.207071046 |  |  |
| Phenylacetylcarnitine levels | chronic periodontitis | MR Egger | 25 | -0.060815088 | 0.130831192 | 0.646419326 | 0.940997225 | 0.7281529 | 1.216057476 |  |  |
| Phenylacetylcarnitine levels | chronic periodontitis | Weighted median | 25 | -0.131807367 | 0.081526155 | 0.105932374 | 0.876509823 | 0.747068326 | 1.028379124 |  |  |
| Phenylacetylcarnitine levels | chronic periodontitis | Inverse variance weighted | 25 | -0.136189174 | 0.056381313 | 0.015713536 | 0.872677529 | 0.781377769 | 0.974645171 | 0.629796945 | FALSE |
| Phenylacetylcarnitine levels | chronic periodontitis | Simple mode | 25 | -0.288253958 | 0.151688994 | 0.069472591 | 0.749571208 | 0.556791527 | 1.009097605 |  |  |
| Phenylacetylcarnitine levels | chronic periodontitis | Weighted mode | 25 | -0.07588435 | 0.128410249 | 0.560077105 | 0.926923399 | 0.720673967 | 1.192199283 |  |  |
| 1-linoleoyl-2-arachidonoyl-GPC (18:2/20:4n6) levels | chronic periodontitis | MR Egger | 37 | 0.100092268 | 0.061795505 | 0.114266343 | 1.105272895 | 0.979192602 | 1.247587216 |  |  |
| 1-linoleoyl-2-arachidonoyl-GPC (18:2/20:4n6) levels | chronic periodontitis | Weighted median | 37 | 0.072369072 | 0.050093324 | 0.148546396 | 1.075052042 | 0.974516485 | 1.185959305 |  |  |
| 1-linoleoyl-2-arachidonoyl-GPC (18:2/20:4n6) levels | chronic periodontitis | Inverse variance weighted | 37 | 0.067980077 | 0.031709344 | 0.032044909 | 1.070343984 | 1.005846788 | 1.138976886 | 0.852187617 | FALSE |
| 1-linoleoyl-2-arachidonoyl-GPC (18:2/20:4n6) levels | chronic periodontitis | Simple mode | 37 | 0.074147446 | 0.087540226 | 0.402585416 | 1.076965588 | 0.907164574 | 1.278549573 |  |  |
| 1-linoleoyl-2-arachidonoyl-GPC (18:2/20:4n6) levels | chronic periodontitis | Weighted mode | 37 | 0.101000845 | 0.056979697 | 0.084761721 | 1.106277577 | 0.989377461 | 1.236990052 |  |  |
| Methylsuccinoylcarnitine levels | chronic periodontitis | MR Egger | 58 | -0.113400506 | 0.069419837 | 0.107964605 | 0.892793019 | 0.779218811 | 1.022921116 |  |  |
| Methylsuccinoylcarnitine levels | chronic periodontitis | Weighted median | 58 | -0.066223167 | 0.043555705 | 0.128404285 | 0.935921974 | 0.85933852 | 1.019330475 |  |  |
| Methylsuccinoylcarnitine levels | chronic periodontitis | Inverse variance weighted | 58 | -0.066811051 | 0.030225198 | 0.027074491 | 0.935371923 | 0.881568618 | 0.992458914 | 0.776834821 | FALSE |
| Methylsuccinoylcarnitine levels | chronic periodontitis | Simple mode | 58 | -0.066253278 | 0.090220078 | 0.465744652 | 0.935893793 | 0.784205143 | 1.116923549 |  |  |
| Methylsuccinoylcarnitine levels | chronic periodontitis | Weighted mode | 58 | -0.081087571 | 0.058445788 | 0.170721182 | 0.922112937 | 0.82230713 | 1.034032466 |  |  |
| Glycocholate glucuronide (1) levels | chronic periodontitis | MR Egger | 17 | 0.12374619 | 0.12536024 | 0.339237817 | 1.13172859 | 0.885183882 | 1.446941849 |  |  |
| Glycocholate glucuronide (1) levels | chronic periodontitis | Weighted median | 17 | 0.092525786 | 0.075629708 | 0.221176649 | 1.096941427 | 0.945814856 | 1.272215684 |  |  |
| Glycocholate glucuronide (1) levels | chronic periodontitis | Inverse variance weighted | 17 | 0.134833976 | 0.055446837 | 0.01502523 | 1.144346779 | 1.026503303 | 1.275718789 | 0.609770502 | FALSE |
| Glycocholate glucuronide (1) levels | chronic periodontitis | Simple mode | 17 | 0.072699588 | 0.110726087 | 0.520788682 | 1.075407424 | 0.865607643 | 1.336056972 |  |  |
| Glycocholate glucuronide (1) levels | chronic periodontitis | Weighted mode | 17 | 0.082591171 | 0.097630314 | 0.410049914 | 1.086097689 | 0.896941851 | 1.315144554 |  |  |
| Glycosyl-N-behenoyl-sphingadienine (d18:2/22:0) levels | chronic periodontitis | MR Egger | 38 | 0.157342812 | 0.079697589 | 0.05606406 | 1.170396771 | 1.001136183 | 1.368273992 |  |  |
| Glycosyl-N-behenoyl-sphingadienine (d18:2/22:0) levels | chronic periodontitis | Weighted median | 38 | 0.127575823 | 0.054131888 | 0.018435198 | 1.136071005 | 1.021709621 | 1.26323302 |  |  |
| Glycosyl-N-behenoyl-sphingadienine (d18:2/22:0) levels | chronic periodontitis | Inverse variance weighted | 38 | 0.080621726 | 0.038522636 | 0.036363412 | 1.083960785 | 1.005130475 | 1.168973594 | 0.852187617 | FALSE |
| Glycosyl-N-behenoyl-sphingadienine (d18:2/22:0) levels | chronic periodontitis | Simple mode | 38 | 0.08731678 | 0.09473732 | 0.362670484 | 1.091242309 | 0.906314985 | 1.313902777 |  |  |
| Glycosyl-N-behenoyl-sphingadienine (d18:2/22:0) levels | chronic periodontitis | Weighted mode | 38 | 0.134010654 | 0.059706403 | 0.030866957 | 1.143405002 | 1.017131188 | 1.285355334 |  |  |
| Perfluorooctanoate (PFOA) levels | chronic periodontitis | MR Egger | 17 | 0.329380835 | 0.145645616 | 0.039014197 | 1.390107156 | 1.044893982 | 1.849372222 |  |  |
| Perfluorooctanoate (PFOA) levels | chronic periodontitis | Weighted median | 17 | 0.180252347 | 0.096501456 | 0.061779944 | 1.197519516 | 0.991148897 | 1.446859291 |  |  |
| Perfluorooctanoate (PFOA) levels | chronic periodontitis | Inverse variance weighted | 17 | 0.210576003 | 0.0692264 | 0.002351327 | 1.234388867 | 1.077768025 | 1.41376979 | 0.339097768 | FALSE |
| Perfluorooctanoate (PFOA) levels | chronic periodontitis | Simple mode | 17 | 0.152674974 | 0.176536969 | 0.39991163 | 1.164946279 | 0.82420377 | 1.646558633 |  |  |
| Perfluorooctanoate (PFOA) levels | chronic periodontitis | Weighted mode | 17 | 0.14467839 | 0.173996532 | 0.417927444 | 1.155667836 | 0.821720625 | 1.625331172 |  |  |
| N-acetyl-2-aminoadipate levels | chronic periodontitis | MR Egger | 24 | 0.137323668 | 0.077709775 | 0.09107654 | 1.1471994 | 0.985124261 | 1.33593955 |  |  |
| N-acetyl-2-aminoadipate levels | chronic periodontitis | Weighted median | 24 | 0.105749419 | 0.062408182 | 0.090174103 | 1.111543309 | 0.983565919 | 1.256172571 |  |  |
| N-acetyl-2-aminoadipate levels | chronic periodontitis | Inverse variance weighted | 24 | 0.0863817 | 0.040982285 | 0.035049895 | 1.090222387 | 1.0060748 | 1.181408036 | 0.852187617 | FALSE |
| N-acetyl-2-aminoadipate levels | chronic periodontitis | Simple mode | 24 | 0.088936304 | 0.114068123 | 0.443530563 | 1.093011034 | 0.874032938 | 1.366851372 |  |  |
| N-acetyl-2-aminoadipate levels | chronic periodontitis | Weighted mode | 24 | 0.104875287 | 0.064046864 | 0.115141398 | 1.110572099 | 0.97955532 | 1.259112541 |  |  |
| 5-oxoproline levels | chronic periodontitis | MR Egger | 32 | 0.041620359 | 0.053427978 | 0.442081991 | 1.042498628 | 0.938851012 | 1.157588771 |  |  |
| 5-oxoproline levels | chronic periodontitis | Weighted median | 32 | 0.018982143 | 0.047458277 | 0.689174635 | 1.01916345 | 0.928638189 | 1.11851327 |  |  |
| 5-oxoproline levels | chronic periodontitis | Inverse variance weighted | 32 | 0.068349694 | 0.034676948 | 0.048719164 | 1.070739674 | 1.000382948 | 1.146044573 | 0.852187617 | FALSE |
| 5-oxoproline levels | chronic periodontitis | Simple mode | 32 | 0.037098366 | 0.110230709 | 0.738722372 | 1.0377951 | 0.836144508 | 1.288077191 |  |  |
| 5-oxoproline levels | chronic periodontitis | Weighted mode | 32 | 0.022339417 | 0.044278515 | 0.617463246 | 1.02259081 | 0.937586299 | 1.115302096 |  |  |
| Flavin adenine dinucleotide (FAD) levels | chronic periodontitis | MR Egger | 27 | 0.084094001 | 0.042610027 | 0.059580458 | 1.087731137 | 1.000578516 | 1.182474946 |  |  |
| Flavin adenine dinucleotide (FAD) levels | chronic periodontitis | Weighted median | 27 | 0.06933697 | 0.044365747 | 0.118088077 | 1.071797312 | 0.982534429 | 1.169169695 |  |  |
| Flavin adenine dinucleotide (FAD) levels | chronic periodontitis | Inverse variance weighted | 27 | 0.071653271 | 0.02891068 | 0.013195975 | 1.074282795 | 1.015101228 | 1.136914715 | 0.519339382 | FALSE |
| Flavin adenine dinucleotide (FAD) levels | chronic periodontitis | Simple mode | 27 | 0.193722927 | 0.090396261 | 0.041639668 | 1.213759936 | 1.016683903 | 1.449037581 |  |  |
| Flavin adenine dinucleotide (FAD) levels | chronic periodontitis | Weighted mode | 27 | 0.061832029 | 0.039127725 | 0.126137366 | 1.063783645 | 0.985251528 | 1.148575375 |  |  |
| Guanidinoacetate levels | chronic periodontitis | MR Egger | 19 | 0.135044489 | 0.12209705 | 0.284115284 | 1.144587705 | 0.900985847 | 1.454052823 |  |  |
| Guanidinoacetate levels | chronic periodontitis | Weighted median | 19 | 0.13781619 | 0.079385897 | 0.082558771 | 1.14776456 | 0.982376967 | 1.340995901 |  |  |
| Guanidinoacetate levels | chronic periodontitis | Inverse variance weighted | 19 | 0.126606928 | 0.057171954 | 0.02679484 | 1.134970804 | 1.014656264 | 1.26955184 | 0.776834821 | FALSE |
| Guanidinoacetate levels | chronic periodontitis | Simple mode | 19 | 0.184505759 | 0.135657718 | 0.190595784 | 1.202623908 | 0.921840221 | 1.568931612 |  |  |
| Guanidinoacetate levels | chronic periodontitis | Weighted mode | 19 | 0.184505759 | 0.101289438 | 0.085182275 | 1.202623908 | 0.986076306 | 1.466726515 |  |  |
| Orotate levels | chronic periodontitis | MR Egger | 53 | -0.113826552 | 0.039628571 | 0.0059231 | 0.892412729 | 0.825720821 | 0.964491217 |  |  |
| Orotate levels | chronic periodontitis | Weighted median | 53 | -0.082325818 | 0.034427704 | 0.016790356 | 0.920971841 | 0.86087659 | 0.985262162 |  |  |
| Orotate levels | chronic periodontitis | Inverse variance weighted | 53 | -0.05185574 | 0.022462803 | 0.020970456 | 0.949465827 | 0.908570463 | 0.992201919 | 0.761660046 | FALSE |
| Orotate levels | chronic periodontitis | Simple mode | 53 | -0.034044713 | 0.06022175 | 0.574285581 | 0.966528287 | 0.858920128 | 1.087617928 |  |  |
| Orotate levels | chronic periodontitis | Weighted mode | 53 | -0.078478448 | 0.034672555 | 0.027810972 | 0.924521985 | 0.863780446 | 0.989534903 |  |  |
| 2'-deoxyuridine levels | chronic periodontitis | MR Egger | 34 | 0.083551878 | 0.080638262 | 0.307907395 | 1.087141613 | 0.928208295 | 1.273288435 |  |  |
| 2'-deoxyuridine levels | chronic periodontitis | Weighted median | 34 | 0.086131595 | 0.063359967 | 0.174019594 | 1.08994975 | 0.962661013 | 1.234069358 |  |  |
| 2'-deoxyuridine levels | chronic periodontitis | Inverse variance weighted | 34 | 0.136132871 | 0.040321628 | 0.000735012 | 1.145834132 | 1.058764308 | 1.240064335 | 0.207139638 | FALSE |
| 2'-deoxyuridine levels | chronic periodontitis | Simple mode | 34 | 0.085152204 | 0.120817903 | 0.485880929 | 1.088882786 | 0.859288218 | 1.379823087 |  |  |
| 2'-deoxyuridine levels | chronic periodontitis | Weighted mode | 34 | 0.085152204 | 0.066195601 | 0.207265122 | 1.088882786 | 0.956388397 | 1.239732441 |  |  |
| Dimethylglycine levels | chronic periodontitis | MR Egger | 42 | 0.100830625 | 0.061052015 | 0.106456173 | 1.106089282 | 0.981344878 | 1.246690667 |  |  |
| Dimethylglycine levels | chronic periodontitis | Weighted median | 42 | 0.050391073 | 0.052845387 | 0.340308085 | 1.0516823 | 0.948203738 | 1.166453597 |  |  |
| Dimethylglycine levels | chronic periodontitis | Inverse variance weighted | 42 | 0.07440843 | 0.034334953 | 0.03022467 | 1.077246695 | 1.007137272 | 1.152236617 | 0.852187617 | FALSE |
| Dimethylglycine levels | chronic periodontitis | Simple mode | 42 | 0.103929336 | 0.091005851 | 0.260076812 | 1.109522048 | 0.928261189 | 1.326177579 |  |  |
| Dimethylglycine levels | chronic periodontitis | Weighted mode | 42 | 0.074093421 | 0.058093132 | 0.209338453 | 1.076907406 | 0.961011294 | 1.206780367 |  |  |
| Dihydroorotate levels | chronic periodontitis | MR Egger | 40 | 0.108928375 | 0.077869552 | 0.169965217 | 1.11508248 | 0.957244966 | 1.29894539 |  |  |
| Dihydroorotate levels | chronic periodontitis | Weighted median | 40 | 0.129638804 | 0.057834236 | 0.024989942 | 1.138417117 | 1.016417004 | 1.275060853 |  |  |
| Dihydroorotate levels | chronic periodontitis | Inverse variance weighted | 40 | 0.111255254 | 0.037597256 | 0.003085067 | 1.117680162 | 1.0382791 | 1.203153319 | 0.339097768 | FALSE |
| Dihydroorotate levels | chronic periodontitis | Simple mode | 40 | 0.094229417 | 0.096816779 | 0.336414579 | 1.098811803 | 0.908889758 | 1.32842005 |  |  |
| Dihydroorotate levels | chronic periodontitis | Weighted mode | 40 | 0.112397761 | 0.061619599 | 0.075812424 | 1.11895785 | 0.991658333 | 1.262598849 |  |  |
| N-stearoyl-sphinganine (d18:0/18:0) levels | chronic periodontitis | MR Egger | 13 | 0.185470202 | 0.076804073 | 0.034318566 | 1.203784329 | 1.035551587 | 1.39934768 |  |  |
| N-stearoyl-sphinganine (d18:0/18:0) levels | chronic periodontitis | Weighted median | 13 | 0.154177665 | 0.071618314 | 0.031336862 | 1.16669815 | 1.013901509 | 1.342521498 |  |  |
| N-stearoyl-sphinganine (d18:0/18:0) levels | chronic periodontitis | Inverse variance weighted | 13 | 0.168094829 | 0.049993187 | 0.000772808 | 1.183048792 | 1.072624215 | 1.304841365 | 0.207139638 | FALSE |
| N-stearoyl-sphinganine (d18:0/18:0) levels | chronic periodontitis | Simple mode | 13 | 0.082244297 | 0.115596915 | 0.490390037 | 1.085721016 | 0.865605811 | 1.361809392 |  |  |
| N-stearoyl-sphinganine (d18:0/18:0) levels | chronic periodontitis | Weighted mode | 13 | 0.180860199 | 0.083351074 | 0.050807571 | 1.198247651 | 1.017645977 | 1.410900712 |  |  |
| X-11787 levels | chronic periodontitis | MR Egger | 40 | -0.080277799 | 0.053865573 | 0.144390467 | 0.922859941 | 0.830394556 | 1.025621453 |  |  |
| X-11787 levels | chronic periodontitis | Weighted median | 40 | -0.084988543 | 0.043530804 | 0.050893457 | 0.918522808 | 0.843404232 | 1.000331889 |  |  |
| X-11787 levels | chronic periodontitis | Inverse variance weighted | 40 | -0.081195898 | 0.029817841 | 0.006467948 | 0.922013053 | 0.869672246 | 0.977503967 | 0.421304208 | FALSE |
| X-11787 levels | chronic periodontitis | Simple mode | 40 | -0.065003132 | 0.077520492 | 0.406844213 | 0.937064529 | 0.804975613 | 1.090827991 |  |  |
| X-11787 levels | chronic periodontitis | Weighted mode | 40 | -0.080485765 | 0.044311337 | 0.07700589 | 0.922668038 | 0.845915348 | 1.006384753 |  |  |
| X-12117 levels | chronic periodontitis | MR Egger | 41 | 0.076317939 | 0.054755118 | 0.171268424 | 1.079305673 | 0.969473544 | 1.201580738 |  |  |
| X-12117 levels | chronic periodontitis | Weighted median | 41 | 0.019807119 | 0.048271319 | 0.681564665 | 1.020004582 | 0.927924724 | 1.121221711 |  |  |
| X-12117 levels | chronic periodontitis | Inverse variance weighted | 41 | 0.095869976 | 0.03154777 | 0.002374572 | 1.100615948 | 1.03462221 | 1.170819119 | 0.339097768 | FALSE |
| X-12117 levels | chronic periodontitis | Simple mode | 41 | 0.06998443 | 0.108733284 | 0.523486193 | 1.072491483 | 0.866638966 | 1.327240091 |  |  |
| X-12117 levels | chronic periodontitis | Weighted mode | 41 | 0.000528489 | 0.045547896 | 0.990800087 | 1.000528629 | 0.915078535 | 1.093958058 |  |  |
| X-12816 levels | chronic periodontitis | MR Egger | 30 | -0.075777136 | 0.073915271 | 0.314049823 | 0.927022784 | 0.801996474 | 1.071539925 |  |  |
| X-12816 levels | chronic periodontitis | Weighted median | 30 | -0.087158952 | 0.059124756 | 0.14044033 | 0.916531399 | 0.816242755 | 1.029142128 |  |  |
| X-12816 levels | chronic periodontitis | Inverse variance weighted | 30 | -0.078630172 | 0.037931449 | 0.038176503 | 0.924381723 | 0.858150478 | 0.995724633 | 0.852187617 | FALSE |
| X-12816 levels | chronic periodontitis | Simple mode | 30 | -0.073578947 | 0.109793394 | 0.508058767 | 0.929062796 | 0.749181502 | 1.152134265 |  |  |
| X-12816 levels | chronic periodontitis | Weighted mode | 30 | -0.088750735 | 0.088980737 | 0.326813231 | 0.915073641 | 0.768624305 | 1.089426607 |  |  |
| X-16397 levels | chronic periodontitis | MR Egger | 16 | -0.143529093 | 0.187619754 | 0.456974257 | 0.866295597 | 0.599737146 | 1.251328296 |  |  |
| X-16397 levels | chronic periodontitis | Weighted median | 16 | -0.189981396 | 0.09280914 | 0.040656936 | 0.826974519 | 0.68943193 | 0.991957038 |  |  |
| X-16397 levels | chronic periodontitis | Inverse variance weighted | 16 | -0.183452051 | 0.071567578 | 0.010367041 | 0.832391787 | 0.723449523 | 0.957739367 | 0.421304208 | FALSE |
| X-16397 levels | chronic periodontitis | Simple mode | 16 | -0.189674822 | 0.137909057 | 0.189212999 | 0.827228086 | 0.631298434 | 1.083966424 |  |  |
| X-16397 levels | chronic periodontitis | Weighted mode | 16 | -0.193806981 | 0.112369001 | 0.105113193 | 0.823816901 | 0.660967755 | 1.026788799 |  |  |
| X-17438 levels | chronic periodontitis | MR Egger | 19 | -0.175883576 | 0.082191264 | 0.047157291 | 0.838715624 | 0.713924222 | 0.985320117 |  |  |
| X-17438 levels | chronic periodontitis | Weighted median | 19 | -0.116105479 | 0.083535115 | 0.164559164 | 0.890381302 | 0.755908981 | 1.048775556 |  |  |
| X-17438 levels | chronic periodontitis | Inverse variance weighted | 19 | -0.129290262 | 0.050970377 | 0.011194227 | 0.87871887 | 0.795175686 | 0.971039314 | 0.421304208 | FALSE |
| X-17438 levels | chronic periodontitis | Simple mode | 19 | -0.071510941 | 0.121955845 | 0.564910242 | 0.930986092 | 0.733047779 | 1.182371911 |  |  |
| X-17438 levels | chronic periodontitis | Weighted mode | 19 | -0.109358401 | 0.079836121 | 0.187600272 | 0.896409086 | 0.766563916 | 1.048248206 |  |  |
| X-18901 levels | chronic periodontitis | MR Egger | 23 | 0.209533839 | 0.091984892 | 0.033304775 | 1.233103101 | 1.029675238 | 1.476721204 |  |  |
| X-18901 levels | chronic periodontitis | Weighted median | 23 | 0.099657729 | 0.072493204 | 0.169218817 | 1.104792715 | 0.958458561 | 1.273468665 |  |  |
| X-18901 levels | chronic periodontitis | Inverse variance weighted | 23 | 0.124077801 | 0.052579627 | 0.018284418 | 1.132103947 | 1.021244245 | 1.254997864 | 0.753085762 | FALSE |
| X-18901 levels | chronic periodontitis | Simple mode | 23 | 0.27447827 | 0.13839467 | 0.059953583 | 1.31584398 | 1.003229922 | 1.725870952 |  |  |
| X-18901 levels | chronic periodontitis | Weighted mode | 23 | 0.033958475 | 0.111342377 | 0.763239374 | 1.034541646 | 0.831709062 | 1.286839914 |  |  |
| X-23654 levels | chronic periodontitis | MR Egger | 44 | -0.105459886 | 0.067998878 | 0.128427604 | 0.899910571 | 0.78762146 | 1.028208445 |  |  |
| X-23654 levels | chronic periodontitis | Weighted median | 44 | -0.055512035 | 0.048725627 | 0.25458722 | 0.946000639 | 0.859835434 | 1.040800568 |  |  |
| X-23654 levels | chronic periodontitis | Inverse variance weighted | 44 | -0.069178879 | 0.034977169 | 0.047947939 | 0.933159743 | 0.871330322 | 0.999376567 | 0.852187617 | FALSE |
| X-23654 levels | chronic periodontitis | Simple mode | 44 | -0.044095403 | 0.086230246 | 0.611709273 | 0.956862665 | 0.808069923 | 1.133053135 |  |  |
| X-23654 levels | chronic periodontitis | Weighted mode | 44 | -0.04036733 | 0.063831349 | 0.530470167 | 0.960436577 | 0.847489546 | 1.088436338 |  |  |
| X-22162 levels | chronic periodontitis | MR Egger | 35 | -0.027281044 | 0.050972919 | 0.596095546 | 0.973087722 | 0.88056814 | 1.075328157 |  |  |
| X-22162 levels | chronic periodontitis | Weighted median | 35 | -0.037168497 | 0.04720959 | 0.431101469 | 0.963513772 | 0.87835952 | 1.056923467 |  |  |
| X-22162 levels | chronic periodontitis | Inverse variance weighted | 35 | -0.073524157 | 0.034033734 | 0.030746993 | 0.9291137 | 0.869158066 | 0.993205151 | 0.852187617 | FALSE |
| X-22162 levels | chronic periodontitis | Simple mode | 35 | -0.063901649 | 0.081253265 | 0.437051181 | 0.938097258 | 0.799988403 | 1.100049029 |  |  |
| X-22162 levels | chronic periodontitis | Weighted mode | 35 | -0.046099399 | 0.046958883 | 0.333183474 | 0.954947037 | 0.870977784 | 1.0470116 |  |  |
| X-23593 levels | chronic periodontitis | MR Egger | 19 | 0.140314055 | 0.126722788 | 0.283606703 | 1.150635104 | 0.897571398 | 1.475048277 |  |  |
| X-23593 levels | chronic periodontitis | Weighted median | 19 | 0.182933223 | 0.08882238 | 0.039442561 | 1.200734224 | 1.008880558 | 1.429071723 |  |  |
| X-23593 levels | chronic periodontitis | Inverse variance weighted | 19 | 0.118389785 | 0.058679028 | 0.043634752 | 1.1256828 | 1.003384605 | 1.262887392 | 0.852187617 | FALSE |
| X-23593 levels | chronic periodontitis | Simple mode | 19 | 0.257110656 | 0.145187446 | 0.093511641 | 1.293188218 | 0.972916772 | 1.718888825 |  |  |
| X-23593 levels | chronic periodontitis | Weighted mode | 19 | 0.242154731 | 0.116793223 | 0.052767123 | 1.273991303 | 1.01332805 | 1.601706219 |  |  |
| X-23739 levels | chronic periodontitis | MR Egger | 33 | 0.21578047 | 0.130841721 | 0.109211225 | 1.24082995 | 0.960146595 | 1.603566552 |  |  |
| X-23739 levels | chronic periodontitis | Weighted median | 33 | 0.081073365 | 0.073865968 | 0.272390558 | 1.084450454 | 0.938282753 | 1.253388474 |  |  |
| X-23739 levels | chronic periodontitis | Inverse variance weighted | 33 | 0.121579352 | 0.051203247 | 0.0175753 | 1.129278973 | 1.021447754 | 1.248493614 | 0.728021067 | FALSE |
| X-23739 levels | chronic periodontitis | Simple mode | 33 | 0.088561097 | 0.142892642 | 0.539795247 | 1.092601006 | 0.825712622 | 1.445753554 |  |  |
| X-23739 levels | chronic periodontitis | Weighted mode | 33 | 0.083745206 | 0.105721263 | 0.434116915 | 1.087351808 | 0.883849509 | 1.337709579 |  |  |
| X-25520 levels | chronic periodontitis | MR Egger | 11 | -0.413514988 | 0.159272138 | 0.028914127 | 0.661321623 | 0.483991285 | 0.903624305 |  |  |
| X-25520 levels | chronic periodontitis | Weighted median | 11 | -0.311012825 | 0.107963909 | 0.003967792 | 0.732704479 | 0.592963706 | 0.905377258 |  |  |
| X-25520 levels | chronic periodontitis | Inverse variance weighted | 11 | -0.233317607 | 0.078289145 | 0.002880567 | 0.79190202 | 0.679251154 | 0.923235544 | 0.339097768 | FALSE |
| X-25520 levels | chronic periodontitis | Simple mode | 11 | -0.346645699 | 0.176412777 | 0.077791781 | 0.707055795 | 0.500366354 | 0.999123727 |  |  |
| X-25520 levels | chronic periodontitis | Weighted mode | 11 | -0.336267416 | 0.161243803 | 0.0636122 | 0.71443203 | 0.520843706 | 0.979973683 |  |  |
| Orotidine levels | chronic periodontitis | MR Egger | 33 | -0.072713767 | 0.030657632 | 0.024093486 | 0.929866951 | 0.875637816 | 0.98745455 |  |  |
| Orotidine levels | chronic periodontitis | Weighted median | 33 | -0.050126944 | 0.03684413 | 0.173667418 | 0.951108679 | 0.884846195 | 1.022333287 |  |  |
| Orotidine levels | chronic periodontitis | Inverse variance weighted | 33 | -0.09076838 | 0.024581206 | 0.000221979 | 0.913229209 | 0.870273678 | 0.958304966 | 0.143620413 | TRUE |
| Orotidine levels | chronic periodontitis | Simple mode | 33 | -0.053282127 | 0.078296574 | 0.501071894 | 0.948112487 | 0.813228289 | 1.105368935 |  |  |
| Orotidine levels | chronic periodontitis | Weighted mode | 33 | -0.059798489 | 0.031372352 | 0.065654446 | 0.941954329 | 0.885778555 | 1.001692752 |  |  |
| N-acetyl-aspartyl-glutamate (naag) levels | chronic periodontitis | MR Egger | 142 | -0.048681274 | 0.019252674 | 0.012562872 | 0.952484663 | 0.917212119 | 0.989113656 |  |  |
| N-acetyl-aspartyl-glutamate (naag) levels | chronic periodontitis | Weighted median | 142 | -0.038039534 | 0.013579498 | 0.005090547 | 0.962674882 | 0.937390479 | 0.988641285 |  |  |
| N-acetyl-aspartyl-glutamate (naag) levels | chronic periodontitis | Inverse variance weighted | 142 | -0.024412929 | 0.010566383 | 0.020864143 | 0.975882657 | 0.955879864 | 0.996304028 | 0.761660046 | FALSE |
| N-acetyl-aspartyl-glutamate (naag) levels | chronic periodontitis | Simple mode | 142 | -0.051008164 | 0.025630041 | 0.048505776 | 0.950270912 | 0.903713363 | 0.999227016 |  |  |
| N-acetyl-aspartyl-glutamate (naag) levels | chronic periodontitis | Weighted mode | 142 | -0.036119799 | 0.012146201 | 0.003461374 | 0.964524738 | 0.941833895 | 0.987762252 |  |  |
| X-12798 levels | chronic periodontitis | MR Egger | 70 | -0.044361886 | 0.031784262 | 0.167340069 | 0.956607712 | 0.898832088 | 1.01809707 |  |  |
| X-12798 levels | chronic periodontitis | Weighted median | 70 | -0.021294918 | 0.033305806 | 0.522578659 | 0.978930218 | 0.917067411 | 1.044966118 |  |  |
| X-12798 levels | chronic periodontitis | Inverse variance weighted | 70 | -0.054552839 | 0.02005001 | 0.006511657 | 0.946908474 | 0.910418533 | 0.984860946 | 0.421304208 | FALSE |
| X-12798 levels | chronic periodontitis | Simple mode | 70 | -0.094909714 | 0.056553387 | 0.097825855 | 0.909455042 | 0.814033017 | 1.016062563 |  |  |
| X-12798 levels | chronic periodontitis | Weighted mode | 70 | -0.01368343 | 0.032125577 | 0.671480428 | 0.986409762 | 0.926214378 | 1.05051729 |  |  |
| N-acetylputrescine to (N(1) + N(8))-acetylspermidine ratio | chronic periodontitis | MR Egger | 56 | 0.041724289 | 0.041183078 | 0.31551072 | 1.042606981 | 0.961755955 | 1.130254832 |  |  |
| N-acetylputrescine to (N(1) + N(8))-acetylspermidine ratio | chronic periodontitis | Weighted median | 56 | 0.045816835 | 0.039878713 | 0.250595341 | 1.046882641 | 0.968172077 | 1.131992226 |  |  |
| N-acetylputrescine to (N(1) + N(8))-acetylspermidine ratio | chronic periodontitis | Inverse variance weighted | 56 | 0.065008963 | 0.023363519 | 0.005394207 | 1.06716859 | 1.01940229 | 1.117173083 | 0.391641301 | FALSE |
| N-acetylputrescine to (N(1) + N(8))-acetylspermidine ratio | chronic periodontitis | Simple mode | 56 | 0.129987257 | 0.084393087 | 0.129231269 | 1.138813871 | 0.965196864 | 1.343660637 |  |  |
| N-acetylputrescine to (N(1) + N(8))-acetylspermidine ratio | chronic periodontitis | Weighted mode | 56 | 0.050289958 | 0.039386854 | 0.207030029 | 1.051575965 | 0.973450526 | 1.135971456 |  |  |
| Aspartate to asparagine ratio | chronic periodontitis | MR Egger | 21 | -0.26071678 | 0.117170313 | 0.038383571 | 0.77049911 | 0.612399401 | 0.969414531 |  |  |
| Aspartate to asparagine ratio | chronic periodontitis | Weighted median | 21 | -0.064218587 | 0.080359036 | 0.42420587 | 0.937799986 | 0.801137811 | 1.097774692 |  |  |
| Aspartate to asparagine ratio | chronic periodontitis | Inverse variance weighted | 21 | -0.107680097 | 0.053591449 | 0.044507704 | 0.897914796 | 0.808382988 | 0.997362627 | 0.852187617 | FALSE |
| Aspartate to asparagine ratio | chronic periodontitis | Simple mode | 21 | -0.058434759 | 0.134081835 | 0.667639208 | 0.943239776 | 0.725252683 | 1.226746616 |  |  |
| Aspartate to asparagine ratio | chronic periodontitis | Weighted mode | 21 | -0.07230723 | 0.091255851 | 0.437450755 | 0.930245053 | 0.777891133 | 1.112438261 |  |  |
| Serine to pyruvate ratio | chronic periodontitis | MR Egger | 18 | -0.136380689 | 0.174343187 | 0.445488496 | 0.872510414 | 0.61996433 | 1.227932618 |  |  |
| Serine to pyruvate ratio | chronic periodontitis | Weighted median | 18 | -0.108512344 | 0.081442528 | 0.18273556 | 0.897167821 | 0.764800932 | 1.052443929 |  |  |
| Serine to pyruvate ratio | chronic periodontitis | Inverse variance weighted | 18 | -0.126343776 | 0.062855532 | 0.04442419 | 0.881311821 | 0.779158632 | 0.996858013 | 0.852187617 | FALSE |
| Serine to pyruvate ratio | chronic periodontitis | Simple mode | 18 | -0.106815351 | 0.117826962 | 0.377318507 | 0.8986916 | 0.713369189 | 1.132157941 |  |  |
| Serine to pyruvate ratio | chronic periodontitis | Weighted mode | 18 | -0.106815351 | 0.117952373 | 0.377814229 | 0.8986916 | 0.713193861 | 1.132436266 |  |  |
| 5-oxoproline to citrate ratio | chronic periodontitis | MR Egger | 27 | 0.057677067 | 0.053021772 | 0.287060022 | 1.059372834 | 0.954807426 | 1.175389687 |  |  |
| 5-oxoproline to citrate ratio | chronic periodontitis | Weighted median | 27 | 0.022374152 | 0.047424619 | 0.63708135 | 1.02262633 | 0.931854957 | 1.122239683 |  |  |
| 5-oxoproline to citrate ratio | chronic periodontitis | Inverse variance weighted | 27 | 0.084778928 | 0.035730889 | 0.01765827 | 1.088476409 | 1.014855649 | 1.167437845 | 0.747794139 | FALSE |
| 5-oxoproline to citrate ratio | chronic periodontitis | Simple mode | 27 | 0.054967612 | 0.118552191 | 0.646751467 | 1.056506396 | 0.837449194 | 1.33286386 |  |  |
| 5-oxoproline to citrate ratio | chronic periodontitis | Weighted mode | 27 | 0.029754901 | 0.046251592 | 0.525645915 | 1.030202001 | 0.940919002 | 1.127956987 |  |  |
| Creatine to carnitine ratio | chronic periodontitis | MR Egger | 22 | -0.124798789 | 0.141380419 | 0.387876626 | 0.882674489 | 0.669044696 | 1.164517494 |  |  |
| Creatine to carnitine ratio | chronic periodontitis | Weighted median | 22 | -0.056491753 | 0.088585069 | 0.523661237 | 0.945074279 | 0.794439465 | 1.124271177 |  |  |
| Creatine to carnitine ratio | chronic periodontitis | Inverse variance weighted | 22 | -0.120270511 | 0.057519812 | 0.036533386 | 0.886680547 | 0.792146173 | 0.992496612 | 0.852187617 | FALSE |
| Creatine to carnitine ratio | chronic periodontitis | Simple mode | 22 | -0.103423089 | 0.156502535 | 0.515899972 | 0.901745375 | 0.663538728 | 1.225466858 |  |  |
| Creatine to carnitine ratio | chronic periodontitis | Weighted mode | 22 | -0.041635554 | 0.091827409 | 0.654903311 | 0.959219301 | 0.801221953 | 1.148373011 |  |  |
| phosphate to asparagine ratio | chronic periodontitis | MR Egger | 38 | -0.124746081 | 0.076108712 | 0.109914052 | 0.882721013 | 0.760393546 | 1.024727776 |  |  |
| phosphate to asparagine ratio | chronic periodontitis | Weighted median | 38 | -0.0737697 | 0.06012041 | 0.219810359 | 0.928885592 | 0.825632348 | 1.045051644 |  |  |
| phosphate to asparagine ratio | chronic periodontitis | Inverse variance weighted | 38 | -0.08045185 | 0.040143094 | 0.045056479 | 0.922699331 | 0.852883504 | 0.998230182 | 0.852187617 | FALSE |
| phosphate to asparagine ratio | chronic periodontitis | Simple mode | 38 | -0.09500496 | 0.101302272 | 0.354410373 | 0.909368424 | 0.745606415 | 1.109098466 |  |  |
| phosphate to asparagine ratio | chronic periodontitis | Weighted mode | 38 | -0.059616106 | 0.072270757 | 0.414715396 | 0.942126141 | 0.817694251 | 1.085493343 |  |  |
| Adenosine 5'-monophosphate (AMP) to valine ratio | chronic periodontitis | MR Egger | 18 | -0.150129146 | 0.088843248 | 0.110449113 | 0.860596827 | 0.72306084 | 1.024294025 |  |  |
| Adenosine 5'-monophosphate (AMP) to valine ratio | chronic periodontitis | Weighted median | 18 | -0.183825111 | 0.089796534 | 0.040645397 | 0.832081313 | 0.69779751 | 0.992206624 |  |  |
| Adenosine 5'-monophosphate (AMP) to valine ratio | chronic periodontitis | Inverse variance weighted | 18 | -0.115300351 | 0.056074066 | 0.039761772 | 0.891098462 | 0.798352124 | 0.994619347 | 0.852187617 | FALSE |
| Adenosine 5'-monophosphate (AMP) to valine ratio | chronic periodontitis | Simple mode | 18 | -0.174995778 | 0.147014716 | 0.250274838 | 0.839460565 | 0.629301616 | 1.119803322 |  |  |
| Adenosine 5'-monophosphate (AMP) to valine ratio | chronic periodontitis | Weighted mode | 18 | -0.199160264 | 0.079089378 | 0.022109404 | 0.819418559 | 0.701751837 | 0.95681513 |  |  |
| Alpha-tocopherol to glycerol ratio | chronic periodontitis | MR Egger | 22 | -0.400450583 | 0.163779971 | 0.023866285 | 0.670018079 | 0.486042444 | 0.923631736 |  |  |
| Alpha-tocopherol to glycerol ratio | chronic periodontitis | Weighted median | 22 | -0.109829396 | 0.102378425 | 0.283370412 | 0.895986982 | 0.733086842 | 1.095085364 |  |  |
| Alpha-tocopherol to glycerol ratio | chronic periodontitis | Inverse variance weighted | 22 | -0.154091525 | 0.071497519 | 0.031146108 | 0.857193563 | 0.745107585 | 0.986140551 | 0.852187617 | FALSE |
| Alpha-tocopherol to glycerol ratio | chronic periodontitis | Simple mode | 22 | -0.109925454 | 0.157720823 | 0.493470366 | 0.895900919 | 0.657665874 | 1.220435007 |  |  |
| Alpha-tocopherol to glycerol ratio | chronic periodontitis | Weighted mode | 22 | -0.08510913 | 0.140983826 | 0.552522092 | 0.918412053 | 0.696674187 | 1.210724776 |  |  |
| Cytidine to N-acetylglucosamine to N-acetylgalactosamine ratio | chronic periodontitis | MR Egger | 20 | 0.025663605 | 0.150937946 | 0.866884915 | 1.02599575 | 0.763246091 | 1.379197734 |  |  |
| Cytidine to N-acetylglucosamine to N-acetylgalactosamine ratio | chronic periodontitis | Weighted median | 20 | 0.082397648 | 0.081626894 | 0.312762509 | 1.085887525 | 0.925342755 | 1.274286432 |  |  |
| Cytidine to N-acetylglucosamine to N-acetylgalactosamine ratio | chronic periodontitis | Inverse variance weighted | 20 | 0.140043057 | 0.062204388 | 0.024364183 | 1.150323328 | 1.018287665 | 1.299479315 | 0.776834821 | FALSE |
| Cytidine to N-acetylglucosamine to N-acetylgalactosamine ratio | chronic periodontitis | Simple mode | 20 | 0.088883897 | 0.128296139 | 0.496815045 | 1.092953754 | 0.849951037 | 1.405431438 |  |  |
| Cytidine to N-acetylglucosamine to N-acetylgalactosamine ratio | chronic periodontitis | Weighted mode | 20 | 0.074728806 | 0.097250067 | 0.451684258 | 1.077591875 | 0.890580907 | 1.303872832 |  |  |
| Phosphate to 5-oxoproline ratio | chronic periodontitis | MR Egger | 29 | -0.081497485 | 0.066961252 | 0.234103718 | 0.921735028 | 0.808365041 | 1.051004706 |  |  |
| Phosphate to 5-oxoproline ratio | chronic periodontitis | Weighted median | 29 | -0.037648197 | 0.053684288 | 0.483122715 | 0.963051685 | 0.866867273 | 1.069908368 |  |  |
| Phosphate to 5-oxoproline ratio | chronic periodontitis | Inverse variance weighted | 29 | -0.0856146 | 0.043064901 | 0.046807641 | 0.91794794 | 0.843646419 | 0.998793335 | 0.852187617 | FALSE |
| Phosphate to 5-oxoproline ratio | chronic periodontitis | Simple mode | 29 | -0.174215441 | 0.117648515 | 0.149822485 | 0.840115883 | 0.667105863 | 1.057995042 |  |  |
| Phosphate to 5-oxoproline ratio | chronic periodontitis | Weighted mode | 29 | -0.0607432 | 0.055059352 | 0.279318656 | 0.941064875 | 0.844796482 | 1.048303487 |  |  |
| Abbreviations: IVW, inverse-variance weighting; MR, Mendelian randomization; SNP, single nucleotide polymorphisms; OR, odds ratio; lci, lower confidence interval; uci, upper confidence interval | | | | | | | | | | | |

**Table S4.** Sensitivity analyses based on the causal effects of the blood metabolites on chronic periodontitis.

| **Exposure** | **Outcome** | **Heterogeneity (Cochran's Q)** | | **Pleiotropy** | |
| --- | --- | --- | --- | --- | --- |
|  |  | **IVW_pvalue** | **MR Egger_pvalue** | **Egger_intercept** | **pvalue (MR Egger)** |
| Maltotriose levels | chronic periodontitis | 0.869184517 | 0.866297335 | 0.012299675 | 0.393332106 |
| 4-guanidinobutanoate levels | chronic periodontitis | 0.117324974 | 0.135088461 | -0.013371112 | 0.200746008 |
| Ribitol levels | chronic periodontitis | 0.543922162 | 0.495352598 | 0.000132001 | 0.987541382 |
| Indoleacetate levels | chronic periodontitis | 0.888188221 | 0.861055345 | -0.004690746 | 0.751330849 |
| 1-methylhistidine levels | chronic periodontitis | 0.717492756 | 0.673478721 | 0.003140882 | 0.829100283 |
| Butyrylglycine levels | chronic periodontitis | 0.449576231 | 0.416015699 | -0.003955138 | 0.668679289 |
| N-acetylhistidine levels | chronic periodontitis | 0.585049804 | 0.561146262 | -0.005631363 | 0.517769571 |
| 5-methyluridine (ribothymidine) levels | chronic periodontitis | 0.979127357 | 0.974387459 | 0.003438768 | 0.660242853 |
| 1-arachidonoyl-GPE (20:4n6) levels | chronic periodontitis | 0.078947704 | 0.065733244 | 0.00062509 | 0.95236709 |
| Docosapentaenoate (n6 DPA; 22:5n6) levels | chronic periodontitis | 0.760234595 | 0.737925782 | -0.012205867 | 0.472801696 |
| Catechol sulfate levels | chronic periodontitis | 0.061494479 | 0.097219995 | 0.021071024 | 0.132066011 |
| Mannitol/sorbitol levels | chronic periodontitis | 0.148834481 | 0.119564144 | 0.002626765 | 0.863445623 |
| 1-palmitoyl-2-linoleoyl-GPE (16:0/18:2) levels | chronic periodontitis | 0.170597435 | 0.155858374 | -0.005335726 | 0.56195771 |
| Succinylcarnitine levels | chronic periodontitis | 0.434576746 | 0.401070515 | 0.004414854 | 0.659108606 |
| Andro steroid monosulfate C19H28O6S (1) levels | chronic periodontitis | 0.980586234 | 0.975647491 | -0.002103155 | 0.760506156 |
| Gamma-CEHC levels | chronic periodontitis | 0.345740745 | 0.31383819 | -0.005137067 | 0.647777955 |
| N-acetyl-beta-alanine levels | chronic periodontitis | 0.182638039 | 0.22591922 | -0.013317434 | 0.152988651 |
| Alliin levels | chronic periodontitis | 0.403624272 | 0.399079879 | 0.011963136 | 0.349732118 |
| 3b-hydroxy-5-cholenoic acid levels | chronic periodontitis | 0.133247582 | 0.111490151 | 0.005710428 | 0.679620779 |
| Glycohyocholate levels | chronic periodontitis | 0.889305814 | 0.847553639 | 0.000958648 | 0.955794338 |
| 9-hydroxystearate levels | chronic periodontitis | 0.211385781 | 0.206639705 | -0.019655783 | 0.378617013 |
| Phenylacetylcarnitine levels | chronic periodontitis | 0.824849746 | 0.802827745 | -0.00928482 | 0.529491431 |
| 1-linoleoyl-2-arachidonoyl-GPC (18:2/20:4n6) levels | chronic periodontitis | 0.521276843 | 0.490979847 | -0.005897163 | 0.548794245 |
| Methylsuccinoylcarnitine levels | chronic periodontitis | 0.123473634 | 0.116458364 | 0.007587691 | 0.458678425 |
| Glycocholate glucuronide (1) levels | chronic periodontitis | 0.2815091 | 0.225577594 | 0.001883469 | 0.922122224 |
| Glycosyl-N-behenoyl-sphingadienine (d18:2/22:0) levels | chronic periodontitis | 0.228201995 | 0.23826948 | -0.011310098 | 0.279187516 |
| Perfluorooctanoate (PFOA) levels | chronic periodontitis | 0.780021976 | 0.780142402 | -0.014476353 | 0.368534944 |
| N-acetyl-2-aminoadipate levels | chronic periodontitis | 0.552711689 | 0.528801618 | -0.009117513 | 0.448584226 |
| 5-oxoproline levels | chronic periodontitis | 0.239779117 | 0.219773414 | 0.00811972 | 0.512972996 |
| Flavin adenine dinucleotide (FAD) levels | chronic periodontitis | 0.739345414 | 0.698578842 | -0.004307078 | 0.694410518 |
| Guanidinoacetate levels | chronic periodontitis | 0.384792882 | 0.322385241 | -0.001101793 | 0.938065608 |
| Orotate levels | chronic periodontitis | 0.69476143 | 0.787829927 | 0.014150032 | 0.063337013 |
| 2'-deoxyuridine levels | chronic periodontitis | 0.503507061 | 0.481778012 | 0.008383668 | 0.456984078 |
| Dimethylglycine levels | chronic periodontitis | 0.405378853 | 0.374831673 | -0.004585265 | 0.602078551 |
| Dihydroorotate levels | chronic periodontitis | 0.213433872 | 0.182488035 | 0.000399105 | 0.972849368 |
| N-stearoyl-sphinganine (d18:0/18:0) levels | chronic periodontitis | 0.917945592 | 0.881637951 | -0.003885296 | 0.771252608 |
| X-11787 levels | chronic periodontitis | 0.430159739 | 0.385898215 | -0.000215969 | 0.983683653 |
| X-12117 levels | chronic periodontitis | 0.532969682 | 0.496314911 | 0.003723017 | 0.664604657 |
| X-12816 levels | chronic periodontitis | 0.482977725 | 0.430130961 | -0.000530932 | 0.96429914 |
| X-16397 levels | chronic periodontitis | 0.294302114 | 0.237754418 | -0.00615754 | 0.820258431 |
| X-17438 levels | chronic periodontitis | 0.751690739 | 0.727679022 | 0.009950187 | 0.479735982 |
| X-18901 levels | chronic periodontitis | 0.761453517 | 0.78338762 | -0.01337434 | 0.270300795 |
| X-23654 levels | chronic periodontitis | 0.860019649 | 0.845272028 | 0.005435899 | 0.537191069 |
| X-22162 levels | chronic periodontitis | 0.273280491 | 0.29508472 | -0.012100755 | 0.234110704 |
| X-23593 levels | chronic periodontitis | 0.639684244 | 0.574970896 | -0.003205912 | 0.847552372 |
| X-23739 levels | chronic periodontitis | 0.285596093 | 0.270992048 | -0.011962961 | 0.439438622 |
| X-25520 levels | chronic periodontitis | 0.564173066 | 0.639506031 | 0.027353155 | 0.226181185 |
| Orotidine levels | chronic periodontitis | 0.598572784 | 0.598732155 | -0.006336058 | 0.332024518 |
| N-acetyl-aspartyl-glutamate (naag) levels | chronic periodontitis | 0.007818235 | 0.009977598 | 0.010402072 | 0.134569048 |
| X-12798 levels | chronic periodontitis | 0.918079828 | 0.906609106 | -0.002605773 | 0.680747518 |
| N-acetylputrescine to (N(1) + N(8))-acetylspermidine ratio | chronic periodontitis | 0.701185214 | 0.683795462 | 0.004809497 | 0.495291308 |
| Aspartate to asparagine ratio | chronic periodontitis | 0.449309302 | 0.523439304 | 0.023538039 | 0.158625046 |
| Serine to pyruvate ratio | chronic periodontitis | 0.943784953 | 0.918204789 | 0.001193681 | 0.951549711 |
| 5-oxoproline to citrate ratio | chronic periodontitis | 0.396610843 | 0.369814962 | 0.00696944 | 0.49180374 |
| Creatine to carnitine ratio | chronic periodontitis | 0.67665035 | 0.616446319 | 0.000575455 | 0.972377906 |
| phosphate to asparagine ratio | chronic periodontitis | 0.54059486 | 0.515731414 | 0.006958396 | 0.497718234 |
| Adenosine 5'-monophosphate (AMP) to valine ratio | chronic periodontitis | 0.374074346 | 0.327354961 | 0.006319828 | 0.614847863 |
| Alpha-tocopherol to glycerol ratio | chronic periodontitis | 0.525190561 | 0.643692002 | 0.026006611 | 0.11011078 |
| Cytidine to N-acetylglucosamine to N-acetylgalactosamine ratio | chronic periodontitis | 0.223071513 | 0.211996715 | 0.015984001 | 0.415706019 |
| Phosphate to 5-oxoproline ratio | chronic periodontitis | 0.062727332 | 0.048577231 | -0.000953187 | 0.935759211 |
| Abbreviations: IVW, inverse-variance weighting; MR, Mendelian randomization. | | | | | |

**Table S5.** Basic Information and Classification of Significant Metabolites Identified by Bidirectional MR.

| **Metabolite** | **Category** | **HMDB ID** | **Metabocard** |
| --- | --- | --- | --- |
| butyrylglycine | Amino acid | HMDB0000808 | https://hmdb.ca/metabolites/HMDB0000808 |
| orotidine | Nucleotide | HMDB0000788 | https://hmdb.ca/metabolites/HMDB0000788 |
| 2'-deoxyuridine | Nucleotide | HMDB0000012 | https://hmdb.ca/metabolites/HMDB0000012 |
| N-stearoyl-sphinganine | Lipid | HMDB0011761 | https://hmdb.ca/metabolites/HMDB0011761 |
| maltotriose | Carbohydrate | HMDB0001262 | https://hmdb.ca/metabolites/HMDB0001262 |
| furaneol sulfate | Xenobiotics | HMDB0341318 | https://hmdb.ca/metabolites/HMDB0341318 |
| N-acetyl-2-aminoadipate | Amino acid | HMDB0062715 | https://hmdb.ca/metabolites/HMDB0062715 |
| pyridoxal | Cofactor and vitamins | HMDB0001545 | https://hmdb.ca/metabolites/HMDB0001545 |
| glycerophosphoethanolamine | Lipid | HMDB0059660 | https://hmdb.ca/metabolites/HMDB0059660 |
| guanidinoacetate | Amino acid | HMDB0000128 | https://hmdb.ca/metabolites/HMDB0000128 |

**Table S6.** Causal effects of chronic periodontitis on the blood metabolites.

| **Exposure** | **Outcome** | **MR method** | **nSNP** | **Beta** | **Se** | **p-value** | **OR** | **or_lci95** | **or_uci95** | **fdr_p-value** | **fdr_significant** |
| --- | --- | --- | --- | --- | --- | --- | --- | --- | --- | --- | --- |
| chronic periodontitis | Glycerophosphorylcholine (GPC) levels | MR Egger | 11 | -0.059392514 | 0.088478033 | 0.518905024 | 0.942336816 | 0.792304525 | 1.120779506 |  |  |
| chronic periodontitis | Glycerophosphorylcholine (GPC) levels | Weighted median | 11 | -0.113620245 | 0.057400113 | 0.047766164 | 0.892596859 | 0.797618819 | 0.998884599 |  |  |
| chronic periodontitis | Glycerophosphorylcholine (GPC) levels | Inverse variance weighted | 11 | -0.099696917 | 0.043799196 | 0.02283242 | 0.905111701 | 0.830652834 | 0.986244984 | 0.999084603 | FALSE |
| chronic periodontitis | Glycerophosphorylcholine (GPC) levels | Simple mode | 11 | -0.120522114 | 0.087837269 | 0.200023157 | 0.886457484 | 0.746258547 | 1.052995473 |  |  |
| chronic periodontitis | Glycerophosphorylcholine (GPC) levels | Weighted mode | 11 | -0.105495919 | 0.077317414 | 0.202341098 | 0.899878145 | 0.773338787 | 1.047122801 |  |  |
| chronic periodontitis | Pyridoxate levels | MR Egger | 11 | -0.176044174 | 0.109626405 | 0.142768205 | 0.838580939 | 0.676439728 | 1.039587064 |  |  |
| chronic periodontitis | Pyridoxate levels | Weighted median | 11 | -0.077387021 | 0.061675199 | 0.209569146 | 0.925531584 | 0.820148045 | 1.044456202 |  |  |
| chronic periodontitis | Pyridoxate levels | Inverse variance weighted | 11 | -0.104416968 | 0.052842864 | 0.048155914 | 0.900849594 | 0.812215987 | 0.999155401 | 0.999084603 | FALSE |
| chronic periodontitis | Pyridoxate levels | Simple mode | 11 | -0.135481105 | 0.091259003 | 0.168479264 | 0.873295664 | 0.730264304 | 1.044341497 |  |  |
| chronic periodontitis | Pyridoxate levels | Weighted mode | 11 | -0.107182062 | 0.082483567 | 0.222952048 | 0.8983621 | 0.764257998 | 1.055997406 |  |  |
| chronic periodontitis | N-acetylthreonine levels | MR Egger | 11 | -0.166868937 | 0.083971659 | 0.0781476 | 0.846310524 | 0.717879617 | 0.997718122 |  |  |
| chronic periodontitis | N-acetylthreonine levels | Weighted median | 11 | -0.083228351 | 0.059739214 | 0.163560948 | 0.920141008 | 0.818471084 | 1.034440301 |  |  |
| chronic periodontitis | N-acetylthreonine levels | Inverse variance weighted | 11 | -0.094944161 | 0.041556351 | 0.022329908 | 0.909423714 | 0.838287122 | 0.986596919 | 0.999084603 | FALSE |
| chronic periodontitis | N-acetylthreonine levels | Simple mode | 11 | -0.044314495 | 0.097274182 | 0.658436289 | 0.956653047 | 0.790593071 | 1.157593062 |  |  |
| chronic periodontitis | N-acetylthreonine levels | Weighted mode | 11 | -0.068564535 | 0.076198014 | 0.389370287 | 0.9337332 | 0.804195691 | 1.084136234 |  |  |
| chronic periodontitis | Stachydrine levels | MR Egger | 11 | -0.202062187 | 0.089039736 | 0.049415678 | 0.817044117 | 0.686204171 | 0.972831581 |  |  |
| chronic periodontitis | Stachydrine levels | Weighted median | 11 | -0.086681229 | 0.066772675 | 0.194234346 | 0.916969352 | 0.804482817 | 1.045184278 |  |  |
| chronic periodontitis | Stachydrine levels | Inverse variance weighted | 11 | -0.117335519 | 0.044445602 | 0.008291082 | 0.889286771 | 0.815096393 | 0.970229984 | 0.999084603 | FALSE |
| chronic periodontitis | Stachydrine levels | Simple mode | 11 | -0.030650163 | 0.109744426 | 0.785715834 | 0.96981479 | 0.782118325 | 1.202555543 |  |  |
| chronic periodontitis | Stachydrine levels | Weighted mode | 11 | -0.036341327 | 0.108162692 | 0.743823451 | 0.964311092 | 0.780094506 | 1.192029778 |  |  |
| chronic periodontitis | 3,7-dimethylurate levels | MR Egger | 11 | 0.015708502 | 0.09732419 | 0.875340481 | 1.015832529 | 0.839417643 | 1.229323372 |  |  |
| chronic periodontitis | 3,7-dimethylurate levels | Weighted median | 11 | 0.107844669 | 0.062704068 | 0.085450791 | 1.113874712 | 0.985057459 | 1.259537567 |  |  |
| chronic periodontitis | 3,7-dimethylurate levels | Inverse variance weighted | 11 | 0.101089276 | 0.04834603 | 0.036532277 | 1.10637541 | 1.006351141 | 1.216341393 | 0.999084603 | FALSE |
| chronic periodontitis | 3,7-dimethylurate levels | Simple mode | 11 | 0.041484322 | 0.092023845 | 0.661750859 | 1.04235682 | 0.87033036 | 1.248385429 |  |  |
| chronic periodontitis | 3,7-dimethylurate levels | Weighted mode | 11 | 0.099969379 | 0.083256934 | 0.257524093 | 1.105137077 | 0.938742363 | 1.30102572 |  |  |
| chronic periodontitis | 1-linoleoyl-GPE (18:2) levels | MR Egger | 11 | -0.068878161 | 0.089258804 | 0.460091603 | 0.933440403 | 0.783624429 | 1.111898702 |  |  |
| chronic periodontitis | 1-linoleoyl-GPE (18:2) levels | Weighted median | 11 | -0.084031778 | 0.058813897 | 0.153069146 | 0.919402039 | 0.819298318 | 1.03173666 |  |  |
| chronic periodontitis | 1-linoleoyl-GPE (18:2) levels | Inverse variance weighted | 11 | -0.091847591 | 0.044120093 | 0.037364093 | 0.912244173 | 0.836672158 | 0.994642195 | 0.999084603 | FALSE |
| chronic periodontitis | 1-linoleoyl-GPE (18:2) levels | Simple mode | 11 | -0.077778202 | 0.086146493 | 0.387831091 | 0.925169605 | 0.78143342 | 1.095344498 |  |  |
| chronic periodontitis | 1-linoleoyl-GPE (18:2) levels | Weighted mode | 11 | -0.102999018 | 0.07812879 | 0.216791969 | 0.90212786 | 0.774040217 | 1.051411357 |  |  |
| chronic periodontitis | Glycerophosphoethanolamine levels | MR Egger | 11 | -0.091280377 | 0.088946737 | 0.331570702 | 0.912761757 | 0.766733511 | 1.086601816 |  |  |
| chronic periodontitis | Glycerophosphoethanolamine levels | Weighted median | 11 | -0.096498285 | 0.059774648 | 0.106447609 | 0.908011455 | 0.807625679 | 1.020874922 |  |  |
| chronic periodontitis | Glycerophosphoethanolamine levels | Inverse variance weighted | 11 | -0.120003625 | 0.044060453 | 0.006457211 | 0.886917221 | 0.81353843 | 0.966914566 | 0.999084603 | FALSE |
| chronic periodontitis | Glycerophosphoethanolamine levels | Simple mode | 11 | -0.088358631 | 0.090430973 | 0.351572348 | 0.915432515 | 0.766743205 | 1.092956135 |  |  |
| chronic periodontitis | Glycerophosphoethanolamine levels | Weighted mode | 11 | -0.085529072 | 0.082862564 | 0.326305131 | 0.918026453 | 0.780407002 | 1.079914157 |  |  |
| chronic periodontitis | 4-vinylphenol sulfate levels | MR Egger | 11 | 0.157886487 | 0.110227014 | 0.18583924 | 1.171033259 | 0.943500427 | 1.453437492 |  |  |
| chronic periodontitis | 4-vinylphenol sulfate levels | Weighted median | 11 | 0.028871879 | 0.064113502 | 0.652476494 | 1.029292712 | 0.907746082 | 1.167114361 |  |  |
| chronic periodontitis | 4-vinylphenol sulfate levels | Inverse variance weighted | 11 | 0.105476894 | 0.052554242 | 0.044748587 | 1.111240428 | 1.002473635 | 1.231808244 | 0.999084603 | FALSE |
| chronic periodontitis | 4-vinylphenol sulfate levels | Simple mode | 11 | 0.025332779 | 0.108403973 | 0.819941133 | 1.025656381 | 0.829328446 | 1.268461267 |  |  |
| chronic periodontitis | 4-vinylphenol sulfate levels | Weighted mode | 11 | 0.025332779 | 0.088314015 | 0.780083572 | 1.025656381 | 0.862635803 | 1.219484524 |  |  |
| chronic periodontitis | 1-palmitoyl-2-linoleoyl-GPE (16:0/18:2) levels | MR Egger | 11 | -0.130081909 | 0.09344784 | 0.197350794 | 0.87802351 | 0.731074671 | 1.054509635 |  |  |
| chronic periodontitis | 1-palmitoyl-2-linoleoyl-GPE (16:0/18:2) levels | Weighted median | 11 | -0.113980794 | 0.062776844 | 0.069424036 | 0.892275093 | 0.78897282 | 1.009103003 |  |  |
| chronic periodontitis | 1-palmitoyl-2-linoleoyl-GPE (16:0/18:2) levels | Inverse variance weighted | 11 | -0.108516544 | 0.044006407 | 0.013665955 | 0.897164052 | 0.823024672 | 0.977982027 | 0.999084603 | FALSE |
| chronic periodontitis | 1-palmitoyl-2-linoleoyl-GPE (16:0/18:2) levels | Simple mode | 11 | -0.184199246 | 0.125777324 | 0.1737781 | 0.83177006 | 0.650039076 | 1.064307453 |  |  |
| chronic periodontitis | 1-palmitoyl-2-linoleoyl-GPE (16:0/18:2) levels | Weighted mode | 11 | -0.220074531 | 0.122528443 | 0.102700815 | 0.802458988 | 0.631138291 | 1.020284201 |  |  |
| chronic periodontitis | Androstenediol (3beta,17beta) disulfate (2) levels | MR Egger | 11 | 0.068058164 | 0.079335161 | 0.413233033 | 1.070427567 | 0.91627499 | 1.250514517 |  |  |
| chronic periodontitis | Androstenediol (3beta,17beta) disulfate (2) levels | Weighted median | 11 | 0.058177657 | 0.055039896 | 0.290507554 | 1.059903278 | 0.951514322 | 1.180639043 |  |  |
| chronic periodontitis | Androstenediol (3beta,17beta) disulfate (2) levels | Inverse variance weighted | 11 | 0.083385374 | 0.039281731 | 0.033774635 | 1.086960614 | 1.006413662 | 1.173954033 | 0.999084603 | FALSE |
| chronic periodontitis | Androstenediol (3beta,17beta) disulfate (2) levels | Simple mode | 11 | 0.068431099 | 0.082191068 | 0.424516026 | 1.070826842 | 0.911500265 | 1.258003062 |  |  |
| chronic periodontitis | Androstenediol (3beta,17beta) disulfate (2) levels | Weighted mode | 11 | 0.03679195 | 0.070859011 | 0.614898569 | 1.037477152 | 0.902946737 | 1.192051309 |  |  |
| chronic periodontitis | 16a-hydroxy DHEA 3-sulfate levels | MR Egger | 11 | 0.215380444 | 0.097176151 | 0.053880365 | 1.240333684 | 1.025228165 | 1.500570996 |  |  |
| chronic periodontitis | 16a-hydroxy DHEA 3-sulfate levels | Weighted median | 11 | 0.136441958 | 0.062408566 | 0.028795877 | 1.146188349 | 1.014221344 | 1.295326449 |  |  |
| chronic periodontitis | 16a-hydroxy DHEA 3-sulfate levels | Inverse variance weighted | 11 | 0.103157049 | 0.049936234 | 0.038849682 | 1.10866551 | 1.005296005 | 1.222663978 | 0.999084603 | FALSE |
| chronic periodontitis | 16a-hydroxy DHEA 3-sulfate levels | Simple mode | 11 | 0.183687929 | 0.10195385 | 0.101773784 | 1.201640767 | 0.98398796 | 1.467437195 |  |  |
| chronic periodontitis | 16a-hydroxy DHEA 3-sulfate levels | Weighted mode | 11 | 0.166677991 | 0.081633336 | 0.068442327 | 1.181373791 | 1.006698991 | 1.386356842 |  |  |
| chronic periodontitis | Cinnamoylglycine levels | MR Egger | 11 | -0.077537134 | 0.092633 | 0.424240363 | 0.92539266 | 0.771747531 | 1.109626582 |  |  |
| chronic periodontitis | Cinnamoylglycine levels | Weighted median | 11 | -0.118324396 | 0.05817967 | 0.041974222 | 0.88840781 | 0.792663448 | 0.995716956 |  |  |
| chronic periodontitis | Cinnamoylglycine levels | Inverse variance weighted | 11 | -0.086469239 | 0.043970526 | 0.049237733 | 0.917163762 | 0.841430829 | 0.999713033 | 0.999084603 | FALSE |
| chronic periodontitis | Cinnamoylglycine levels | Simple mode | 11 | -0.126122157 | 0.090483239 | 0.193554016 | 0.881507158 | 0.73825255 | 1.052559683 |  |  |
| chronic periodontitis | Cinnamoylglycine levels | Weighted mode | 11 | -0.11740142 | 0.087226432 | 0.208040899 | 0.889228168 | 0.749487809 | 1.05502281 |  |  |
| chronic periodontitis | Fructosyllysine levels | MR Egger | 11 | 0.028085809 | 0.108987877 | 0.802436576 | 1.028483934 | 0.830663559 | 1.273414719 |  |  |
| chronic periodontitis | Fructosyllysine levels | Weighted median | 11 | 0.050565667 | 0.06374907 | 0.427662182 | 1.051865934 | 0.928316535 | 1.191858489 |  |  |
| chronic periodontitis | Fructosyllysine levels | Inverse variance weighted | 11 | 0.116334419 | 0.053551539 | 0.029826835 | 1.123371486 | 1.011438325 | 1.24769199 | 0.999084603 | FALSE |
| chronic periodontitis | Fructosyllysine levels | Simple mode | 11 | 0.060154387 | 0.073773781 | 0.43384028 | 1.062000493 | 0.919024752 | 1.227219446 |  |  |
| chronic periodontitis | Fructosyllysine levels | Weighted mode | 11 | 0.048089641 | 0.073577545 | 0.528117071 | 1.049264708 | 0.908352874 | 1.212036048 |  |  |
| chronic periodontitis | N-palmitoyl-sphinganine (d18:0/16:0) levels | MR Egger | 11 | -0.041398852 | 0.08888331 | 0.652448649 | 0.959446376 | 0.806049487 | 1.142035772 |  |  |
| chronic periodontitis | N-palmitoyl-sphinganine (d18:0/16:0) levels | Weighted median | 11 | -0.070082411 | 0.058199236 | 0.228519645 | 0.932316984 | 0.8318086 | 1.04496991 |  |  |
| chronic periodontitis | N-palmitoyl-sphinganine (d18:0/16:0) levels | Inverse variance weighted | 11 | -0.08648689 | 0.043964707 | 0.049161418 | 0.917147572 | 0.841425572 | 0.999683985 | 0.999084603 | FALSE |
| chronic periodontitis | N-palmitoyl-sphinganine (d18:0/16:0) levels | Simple mode | 11 | -0.053691451 | 0.085730353 | 0.545159092 | 0.94772448 | 0.801137294 | 1.121133291 |  |  |
| chronic periodontitis | N-palmitoyl-sphinganine (d18:0/16:0) levels | Weighted mode | 11 | -0.027949263 | 0.085158202 | 0.74952618 | 0.972437705 | 0.822950408 | 1.14907907 |  |  |
| chronic periodontitis | 1-oleoyl-2-linoleoyl-GPE (18:1/18:2) levels | MR Egger | 11 | -0.168548569 | 0.092917454 | 0.103085815 | 0.844890227 | 0.704218375 | 1.013662127 |  |  |
| chronic periodontitis | 1-oleoyl-2-linoleoyl-GPE (18:1/18:2) levels | Weighted median | 11 | -0.097019925 | 0.064245565 | 0.131007201 | 0.907537924 | 0.800161872 | 1.029323081 |  |  |
| chronic periodontitis | 1-oleoyl-2-linoleoyl-GPE (18:1/18:2) levels | Inverse variance weighted | 11 | -0.105421731 | 0.04505038 | 0.019279337 | 0.899944908 | 0.823888168 | 0.983022779 | 0.999084603 | FALSE |
| chronic periodontitis | 1-oleoyl-2-linoleoyl-GPE (18:1/18:2) levels | Simple mode | 11 | -0.133933187 | 0.13018057 | 0.327803251 | 0.8746485 | 0.677675238 | 1.128874062 |  |  |
| chronic periodontitis | 1-oleoyl-2-linoleoyl-GPE (18:1/18:2) levels | Weighted mode | 11 | -0.212567011 | 0.12443048 | 0.11837646 | 0.808506136 | 0.633528209 | 1.031812257 |  |  |
| chronic periodontitis | Furaneol sulfate levels | MR Egger | 11 | 0.186475716 | 0.106791981 | 0.114734984 | 1.20499536 | 0.977422214 | 1.485554345 |  |  |
| chronic periodontitis | Furaneol sulfate levels | Weighted median | 11 | 0.217040369 | 0.072047044 | 0.002591214 | 1.242394255 | 1.078777184 | 1.430826966 |  |  |
| chronic periodontitis | Furaneol sulfate levels | Inverse variance weighted | 11 | 0.216421637 | 0.052480876 | 3.73E-05 | 1.241625784 | 1.120258116 | 1.376142305 | 0.052132569 | TRUE |
| chronic periodontitis | Furaneol sulfate levels | Simple mode | 11 | 0.292065913 | 0.110350459 | 0.024450648 | 1.339191285 | 1.078724165 | 1.662550406 |  |  |
| chronic periodontitis | Furaneol sulfate levels | Weighted mode | 11 | 0.256915257 | 0.094389087 | 0.021491065 | 1.292935554 | 1.074561472 | 1.555687962 |  |  |
| chronic periodontitis | Perfluorooctanesulfonate (PFOS) levels | MR Egger | 11 | -0.083851104 | 0.083272381 | 0.340262654 | 0.919568166 | 0.781089944 | 1.082596978 |  |  |
| chronic periodontitis | Perfluorooctanesulfonate (PFOS) levels | Weighted median | 11 | -0.063724338 | 0.056897776 | 0.262722538 | 0.938263608 | 0.839252236 | 1.048955916 |  |  |
| chronic periodontitis | Perfluorooctanesulfonate (PFOS) levels | Inverse variance weighted | 11 | -0.107650676 | 0.040649111 | 0.008089985 | 0.897941214 | 0.829175924 | 0.972409353 | 0.999084603 | FALSE |
| chronic periodontitis | Perfluorooctanesulfonate (PFOS) levels | Simple mode | 11 | -0.108462401 | 0.09025566 | 0.257156733 | 0.897212628 | 0.751740951 | 1.070834972 |  |  |
| chronic periodontitis | Perfluorooctanesulfonate (PFOS) levels | Weighted mode | 11 | -0.07564987 | 0.074225376 | 0.332135898 | 0.927140769 | 0.801611176 | 1.072327871 |  |  |
| chronic periodontitis | Glycosyl ceramide (d18:2/24:1, d18:1/24:2) levels | MR Egger | 11 | -0.008251188 | 0.086189051 | 0.925829908 | 0.99178276 | 0.83762755 | 1.174308369 |  |  |
| chronic periodontitis | Glycosyl ceramide (d18:2/24:1, d18:1/24:2) levels | Weighted median | 11 | -0.101202591 | 0.057360759 | 0.077678537 | 0.903749922 | 0.807647419 | 1.011287727 |  |  |
| chronic periodontitis | Glycosyl ceramide (d18:2/24:1, d18:1/24:2) levels | Inverse variance weighted | 11 | -0.106198542 | 0.042622471 | 0.012716586 | 0.899246093 | 0.827175342 | 0.977596277 | 0.999084603 | FALSE |
| chronic periodontitis | Glycosyl ceramide (d18:2/24:1, d18:1/24:2) levels | Simple mode | 11 | -0.108508428 | 0.08303319 | 0.220530578 | 0.897171333 | 0.762423212 | 1.055734385 |  |  |
| chronic periodontitis | Glycosyl ceramide (d18:2/24:1, d18:1/24:2) levels | Weighted mode | 11 | -0.081874708 | 0.076221092 | 0.307980047 | 0.921387394 | 0.793526734 | 1.069850194 |  |  |
| chronic periodontitis | Carotene diol (2) levels | MR Egger | 11 | -0.220998903 | 0.094031324 | 0.043286006 | 0.80171756 | 0.66677655 | 0.963967684 |  |  |
| chronic periodontitis | Carotene diol (2) levels | Weighted median | 11 | -0.129139668 | 0.059180631 | 0.029100142 | 0.87885121 | 0.782599895 | 0.986940395 |  |  |
| chronic periodontitis | Carotene diol (2) levels | Inverse variance weighted | 11 | -0.099628598 | 0.049204361 | 0.042888722 | 0.905173539 | 0.821955399 | 0.996817025 | 0.999084603 | FALSE |
| chronic periodontitis | Carotene diol (2) levels | Simple mode | 11 | -0.124358785 | 0.084333354 | 0.171090569 | 0.883062954 | 0.74852389 | 1.041783959 |  |  |
| chronic periodontitis | Carotene diol (2) levels | Weighted mode | 11 | -0.13937169 | 0.072381199 | 0.083047111 | 0.869904634 | 0.754848026 | 1.002498576 |  |  |
| chronic periodontitis | Glyco-beta-muricholate levels | MR Egger | 11 | 0.14177484 | 0.0978457 | 0.181277971 | 1.152317163 | 0.951226826 | 1.395918206 |  |  |
| chronic periodontitis | Glyco-beta-muricholate levels | Weighted median | 11 | 0.133680169 | 0.062162004 | 0.031514696 | 1.143027185 | 1.011913042 | 1.291129861 |  |  |
| chronic periodontitis | Glyco-beta-muricholate levels | Inverse variance weighted | 11 | 0.100092358 | 0.047795152 | 0.036242631 | 1.105272994 | 1.006434473 | 1.213818111 | 0.999084603 | FALSE |
| chronic periodontitis | Glyco-beta-muricholate levels | Simple mode | 11 | 0.124008232 | 0.089797683 | 0.197366143 | 1.13202519 | 0.949333399 | 1.349874589 |  |  |
| chronic periodontitis | Glyco-beta-muricholate levels | Weighted mode | 11 | 0.138181092 | 0.072522189 | 0.085859505 | 1.148183458 | 0.996045442 | 1.323559346 |  |  |
| chronic periodontitis | Ascorbic acid 3-sulfate levels | MR Egger | 11 | -0.175212027 | 0.086516092 | 0.073502671 | 0.839279052 | 0.708373652 | 0.994375391 |  |  |
| chronic periodontitis | Ascorbic acid 3-sulfate levels | Weighted median | 11 | -0.096227794 | 0.057009572 | 0.091425921 | 0.908257097 | 0.812234202 | 1.015631886 |  |  |
| chronic periodontitis | Ascorbic acid 3-sulfate levels | Inverse variance weighted | 11 | -0.099147969 | 0.042845275 | 0.020662598 | 0.905608696 | 0.832664309 | 0.984943273 | 0.999084603 | FALSE |
| chronic periodontitis | Ascorbic acid 3-sulfate levels | Simple mode | 11 | -0.098833514 | 0.093172791 | 0.313748481 | 0.905893514 | 0.754687 | 1.087395249 |  |  |
| chronic periodontitis | Ascorbic acid 3-sulfate levels | Weighted mode | 11 | -0.098833514 | 0.077064661 | 0.228605955 | 0.905893514 | 0.77889405 | 1.053600369 |  |  |
| chronic periodontitis | 11beta-hydroxyandrosterone glucuronide levels | MR Egger | 11 | 0.134367597 | 0.08534956 | 0.149865379 | 1.143813205 | 0.967618345 | 1.352091611 |  |  |
| chronic periodontitis | 11beta-hydroxyandrosterone glucuronide levels | Weighted median | 11 | 0.100399456 | 0.058045157 | 0.083687746 | 1.105612473 | 0.986719917 | 1.238830716 |  |  |
| chronic periodontitis | 11beta-hydroxyandrosterone glucuronide levels | Inverse variance weighted | 11 | 0.085737589 | 0.042145782 | 0.041920342 | 1.089520389 | 1.003136766 | 1.183342808 | 0.999084603 | FALSE |
| chronic periodontitis | 11beta-hydroxyandrosterone glucuronide levels | Simple mode | 11 | 0.175122364 | 0.095506694 | 0.096603118 | 1.191391991 | 0.988001804 | 1.43665211 |  |  |
| chronic periodontitis | 11beta-hydroxyandrosterone glucuronide levels | Weighted mode | 11 | 0.117936269 | 0.084075274 | 0.190968205 | 1.125172401 | 0.954229311 | 1.326738676 |  |  |
| chronic periodontitis | 2-hydroxy-4-(methylthio)butanoic acid levels | MR Egger | 11 | 0.142281649 | 0.087135621 | 0.136929006 | 1.152901316 | 0.971898243 | 1.367613795 |  |  |
| chronic periodontitis | 2-hydroxy-4-(methylthio)butanoic acid levels | Weighted median | 11 | 0.093769484 | 0.053700662 | 0.080784656 | 1.098306539 | 0.988581873 | 1.220209764 |  |  |
| chronic periodontitis | 2-hydroxy-4-(methylthio)butanoic acid levels | Inverse variance weighted | 11 | 0.085398679 | 0.043099321 | 0.047542057 | 1.089151202 | 1.000924436 | 1.185154741 | 0.999084603 | FALSE |
| chronic periodontitis | 2-hydroxy-4-(methylthio)butanoic acid levels | Simple mode | 11 | 0.093111743 | 0.080263407 | 0.272964567 | 1.097584376 | 0.937813164 | 1.284575125 |  |  |
| chronic periodontitis | 2-hydroxy-4-(methylthio)butanoic acid levels | Weighted mode | 11 | 0.099852524 | 0.073625955 | 0.204861105 | 1.105007944 | 0.956519267 | 1.27654779 |  |  |
| chronic periodontitis | N-acetyl-2-aminoadipate levels | MR Egger | 11 | 0.151039586 | 0.095769657 | 0.149224447 | 1.163042697 | 0.963995221 | 1.403189857 |  |  |
| chronic periodontitis | N-acetyl-2-aminoadipate levels | Weighted median | 11 | 0.178024904 | 0.062447039 | 0.004360745 | 1.194855078 | 1.057205079 | 1.350427354 |  |  |
| chronic periodontitis | N-acetyl-2-aminoadipate levels | Inverse variance weighted | 11 | 0.148166964 | 0.046059343 | 0.001295969 | 1.159706509 | 1.059599125 | 1.269271705 | 0.752528418 | FALSE |
| chronic periodontitis | N-acetyl-2-aminoadipate levels | Simple mode | 11 | 0.232609496 | 0.093847979 | 0.032622193 | 1.261888611 | 1.049871165 | 1.516722165 |  |  |
| chronic periodontitis | N-acetyl-2-aminoadipate levels | Weighted mode | 11 | 0.189221816 | 0.080953895 | 0.041523036 | 1.208308945 | 1.03102369 | 1.416078526 |  |  |
| chronic periodontitis | 2-methoxyhydroquinone sulfate (1) levels | MR Egger | 11 | 0.076794377 | 0.092841082 | 0.429531579 | 1.079820017 | 0.90016775 | 1.295326644 |  |  |
| chronic periodontitis | 2-methoxyhydroquinone sulfate (1) levels | Weighted median | 11 | 0.090551305 | 0.063234452 | 0.152145767 | 1.094777674 | 0.967163014 | 1.239230758 |  |  |
| chronic periodontitis | 2-methoxyhydroquinone sulfate (1) levels | Inverse variance weighted | 11 | 0.096002656 | 0.045783308 | 0.036003128 | 1.100761988 | 1.006287054 | 1.20410667 | 0.999084603 | FALSE |
| chronic periodontitis | 2-methoxyhydroquinone sulfate (1) levels | Simple mode | 11 | 0.068773138 | 0.095411943 | 0.487539377 | 1.071193168 | 0.888487882 | 1.291469278 |  |  |
| chronic periodontitis | 2-methoxyhydroquinone sulfate (1) levels | Weighted mode | 11 | 0.075947784 | 0.082989677 | 0.381651924 | 1.078906236 | 0.916941152 | 1.269480232 |  |  |
| chronic periodontitis | 3-(4-hydroxyphenyl)lactate levels | MR Egger | 11 | 0.08335545 | 0.084774567 | 0.351171915 | 1.086928089 | 0.92053275 | 1.28340102 |  |  |
| chronic periodontitis | 3-(4-hydroxyphenyl)lactate levels | Weighted median | 11 | 0.112679115 | 0.056394879 | 0.045712533 | 1.119272717 | 1.002147455 | 1.250086911 |  |  |
| chronic periodontitis | 3-(4-hydroxyphenyl)lactate levels | Inverse variance weighted | 11 | 0.092475494 | 0.041880878 | 0.027240087 | 1.096886261 | 1.010443126 | 1.190724583 | 0.999084603 | FALSE |
| chronic periodontitis | 3-(4-hydroxyphenyl)lactate levels | Simple mode | 11 | 0.116606803 | 0.088888453 | 0.218892632 | 1.123677516 | 0.944013733 | 1.337534736 |  |  |
| chronic periodontitis | 3-(4-hydroxyphenyl)lactate levels | Weighted mode | 11 | 0.12064415 | 0.084248975 | 0.182651394 | 1.128223363 | 0.956491055 | 1.330789189 |  |  |
| chronic periodontitis | Guanidinoacetate levels | MR Egger | 11 | -0.073170777 | 0.094521384 | 0.458712302 | 0.929442089 | 0.772261009 | 1.118614802 |  |  |
| chronic periodontitis | Guanidinoacetate levels | Weighted median | 11 | -0.096208275 | 0.057640169 | 0.095093889 | 0.908274825 | 0.811246758 | 1.016907802 |  |  |
| chronic periodontitis | Guanidinoacetate levels | Inverse variance weighted | 11 | -0.121553528 | 0.045169936 | 0.007123237 | 0.885543651 | 0.810514051 | 0.96751877 | 0.999084603 | FALSE |
| chronic periodontitis | Guanidinoacetate levels | Simple mode | 11 | -0.084867504 | 0.079393183 | 0.310215117 | 0.918633992 | 0.78625183 | 1.073305496 |  |  |
| chronic periodontitis | Guanidinoacetate levels | Weighted mode | 11 | -0.079816181 | 0.071080921 | 0.287718982 | 0.923286048 | 0.803213449 | 1.061308334 |  |  |
| chronic periodontitis | Citrulline levels | MR Egger | 11 | -0.115430994 | 0.104276808 | 0.297015917 | 0.890982053 | 0.726284449 | 1.093027697 |  |  |
| chronic periodontitis | Citrulline levels | Weighted median | 11 | -0.114588162 | 0.063705708 | 0.072064397 | 0.891733318 | 0.787059563 | 1.010327995 |  |  |
| chronic periodontitis | Citrulline levels | Inverse variance weighted | 11 | -0.102458747 | 0.048796576 | 0.0357542 | 0.902615384 | 0.82028779 | 0.993205728 | 0.999084603 | FALSE |
| chronic periodontitis | Citrulline levels | Simple mode | 11 | -0.149883702 | 0.094267326 | 0.14292215 | 0.860808081 | 0.715590173 | 1.035495707 |  |  |
| chronic periodontitis | Citrulline levels | Weighted mode | 11 | -0.12733717 | 0.08026966 | 0.143739387 | 0.880436766 | 0.752265692 | 1.030445635 |  |  |
| chronic periodontitis | Pyridoxal levels | MR Egger | 11 | -0.103044865 | 0.09051834 | 0.284354021 | 0.902086501 | 0.755435548 | 1.077206464 |  |  |
| chronic periodontitis | Pyridoxal levels | Weighted median | 11 | -0.108073806 | 0.060226762 | 0.072741656 | 0.897561348 | 0.797623769 | 1.01002052 |  |  |
| chronic periodontitis | Pyridoxal levels | Inverse variance weighted | 11 | -0.141196701 | 0.044775785 | 0.001613714 | 0.868318496 | 0.795362544 | 0.947966455 | 0.752528418 | FALSE |
| chronic periodontitis | Pyridoxal levels | Simple mode | 11 | -0.11298403 | 0.081036347 | 0.193446837 | 0.893164924 | 0.761995011 | 1.046914442 |  |  |
| chronic periodontitis | Pyridoxal levels | Weighted mode | 11 | -0.104422728 | 0.079292056 | 0.217238941 | 0.900844405 | 0.771178698 | 1.052312056 |  |  |
| chronic periodontitis | X-11381 levels | MR Egger | 11 | 0.207994909 | 0.087898007 | 0.042162388 | 1.231206901 | 1.03636025 | 1.462686777 |  |  |
| chronic periodontitis | X-11381 levels | Weighted median | 11 | 0.179748355 | 0.059536128 | 0.00253487 | 1.196916127 | 1.065088128 | 1.345060732 |  |  |
| chronic periodontitis | X-11381 levels | Inverse variance weighted | 11 | 0.100122927 | 0.043539676 | 0.021472079 | 1.105306782 | 1.014895004 | 1.20377288 | 0.999084603 | FALSE |
| chronic periodontitis | X-11381 levels | Simple mode | 11 | 0.181090805 | 0.10136765 | 0.104323348 | 1.198524007 | 0.982564009 | 1.461950348 |  |  |
| chronic periodontitis | X-11381 levels | Weighted mode | 11 | 0.181090805 | 0.077717482 | 0.042041846 | 1.198524007 | 1.029182235 | 1.395729294 |  |  |
| chronic periodontitis | X-11795 levels | MR Egger | 11 | 0.200018164 | 0.086351402 | 0.045760064 | 1.221424944 | 1.031247686 | 1.446673688 |  |  |
| chronic periodontitis | X-11795 levels | Weighted median | 11 | 0.116094551 | 0.062041733 | 0.061312763 | 1.123102058 | 0.994507892 | 1.268324005 |  |  |
| chronic periodontitis | X-11795 levels | Inverse variance weighted | 11 | 0.091064511 | 0.042760684 | 0.033202024 | 1.095339665 | 1.007279942 | 1.191097858 | 0.999084603 | FALSE |
| chronic periodontitis | X-11795 levels | Simple mode | 11 | 0.173622658 | 0.103366108 | 0.123940297 | 1.189606592 | 0.971440834 | 1.45676792 |  |  |
| chronic periodontitis | X-11795 levels | Weighted mode | 11 | 0.158653564 | 0.079772096 | 0.074765414 | 1.171931877 | 1.002302904 | 1.370268728 |  |  |
| chronic periodontitis | X-12026 levels | MR Egger | 11 | 0.101498218 | 0.086554812 | 0.271039808 | 1.106827946 | 0.934121079 | 1.311466072 |  |  |
| chronic periodontitis | X-12026 levels | Weighted median | 11 | 0.08901746 | 0.059517685 | 0.134745611 | 1.093099742 | 0.972741211 | 1.22835039 |  |  |
| chronic periodontitis | X-12026 levels | Inverse variance weighted | 11 | 0.092466786 | 0.042090492 | 0.028030674 | 1.096876709 | 1.01001928 | 1.191203512 | 0.999084603 | FALSE |
| chronic periodontitis | X-12026 levels | Simple mode | 11 | 0.191287459 | 0.106466579 | 0.102603804 | 1.21080746 | 0.982763247 | 1.49176794 |  |  |
| chronic periodontitis | X-12026 levels | Weighted mode | 11 | 0.165642196 | 0.098256752 | 0.122730997 | 1.180150763 | 0.973418635 | 1.430788126 |  |  |
| chronic periodontitis | X-12818 levels | MR Egger | 11 | 0.146151385 | 0.10364074 | 0.192099794 | 1.157371384 | 0.944608743 | 1.418056449 |  |  |
| chronic periodontitis | X-12818 levels | Weighted median | 11 | 0.108425886 | 0.063408861 | 0.087275396 | 1.114522304 | 0.984269556 | 1.262011976 |  |  |
| chronic periodontitis | X-12818 levels | Inverse variance weighted | 11 | 0.101502305 | 0.050805518 | 0.045731855 | 1.10683247 | 1.001925341 | 1.222723956 | 0.999084603 | FALSE |
| chronic periodontitis | X-12818 levels | Simple mode | 11 | 0.149260459 | 0.089497504 | 0.126326294 | 1.160975337 | 0.974184422 | 1.383581694 |  |  |
| chronic periodontitis | X-12818 levels | Weighted mode | 11 | 0.115103571 | 0.080954218 | 0.185504584 | 1.121989637 | 0.957368699 | 1.314917385 |  |  |
| chronic periodontitis | X-12906 levels | MR Egger | 11 | -0.100800622 | 0.089770721 | 0.290553748 | 0.904113276 | 0.758243098 | 1.078045837 |  |  |
| chronic periodontitis | X-12906 levels | Weighted median | 11 | -0.069673791 | 0.06052879 | 0.249697208 | 0.932698025 | 0.828357694 | 1.050181113 |  |  |
| chronic periodontitis | X-12906 levels | Inverse variance weighted | 11 | -0.106220783 | 0.044302031 | 0.016500604 | 0.899226092 | 0.824438473 | 0.980797951 | 0.999084603 | FALSE |
| chronic periodontitis | X-12906 levels | Simple mode | 11 | -0.03256068 | 0.099693725 | 0.750698121 | 0.967963712 | 0.796155801 | 1.176847227 |  |  |
| chronic periodontitis | X-12906 levels | Weighted mode | 11 | -0.061552771 | 0.093924326 | 0.527033873 | 0.940303324 | 0.782200287 | 1.1303631 |  |  |
| chronic periodontitis | X-15461 levels | MR Egger | 11 | 0.1985162 | 0.08966242 | 0.054087533 | 1.219591785 | 1.023039253 | 1.453907186 |  |  |
| chronic periodontitis | X-15461 levels | Weighted median | 11 | 0.131892829 | 0.059359151 | 0.026286954 | 1.140986032 | 1.015670406 | 1.281763372 |  |  |
| chronic periodontitis | X-15461 levels | Inverse variance weighted | 11 | 0.091988033 | 0.046146682 | 0.046219471 | 1.096351702 | 1.001541724 | 1.200136775 | 0.999084603 | FALSE |
| chronic periodontitis | X-15461 levels | Simple mode | 11 | 0.127457578 | 0.098838175 | 0.226230536 | 1.135936679 | 0.935882593 | 1.378754288 |  |  |
| chronic periodontitis | X-15461 levels | Weighted mode | 11 | 0.134106802 | 0.079797408 | 0.123761006 | 1.143514943 | 0.977950604 | 1.337108869 |  |  |
| chronic periodontitis | X-21364 levels | MR Egger | 11 | 0.098951149 | 0.085270661 | 0.275726695 | 1.104012366 | 0.934092932 | 1.304841587 |  |  |
| chronic periodontitis | X-21364 levels | Weighted median | 11 | 0.091677672 | 0.061560483 | 0.136427009 | 1.09601149 | 0.971435044 | 1.23656357 |  |  |
| chronic periodontitis | X-21364 levels | Inverse variance weighted | 11 | 0.095899364 | 0.0421298 | 0.022829053 | 1.100648293 | 1.013414129 | 1.195391529 | 0.999084603 | FALSE |
| chronic periodontitis | X-21364 levels | Simple mode | 11 | 0.212229495 | 0.09626139 | 0.052023656 | 1.236431608 | 1.023836833 | 1.49317066 |  |  |
| chronic periodontitis | X-21364 levels | Weighted mode | 11 | 0.110167171 | 0.081830831 | 0.207934731 | 1.116464695 | 0.951018981 | 1.310692468 |  |  |
| chronic periodontitis | X-23587 levels | MR Egger | 11 | 0.134190266 | 0.092680059 | 0.181570944 | 1.143610389 | 0.95364609 | 1.371415177 |  |  |
| chronic periodontitis | X-23587 levels | Weighted median | 11 | 0.120140279 | 0.061715481 | 0.051573138 | 1.127655027 | 0.999178274 | 1.272651631 |  |  |
| chronic periodontitis | X-23587 levels | Inverse variance weighted | 11 | 0.105892541 | 0.045894073 | 0.021036711 | 1.111702408 | 1.016067879 | 1.216338267 | 0.999084603 | FALSE |
| chronic periodontitis | X-23587 levels | Simple mode | 11 | 0.124137004 | 0.089004631 | 0.193304422 | 1.132170972 | 0.950932617 | 1.347951565 |  |  |
| chronic periodontitis | X-23587 levels | Weighted mode | 11 | 0.120617597 | 0.082579247 | 0.174812042 | 1.128193405 | 0.959600979 | 1.326405857 |  |  |
| chronic periodontitis | X-25172 levels | MR Egger | 11 | 0.114809631 | 0.090630212 | 0.237029164 | 1.121659888 | 0.939107255 | 1.339698845 |  |  |
| chronic periodontitis | X-25172 levels | Weighted median | 11 | 0.103543567 | 0.059917771 | 0.083971293 | 1.109094112 | 0.98620083 | 1.247301475 |  |  |
| chronic periodontitis | X-25172 levels | Inverse variance weighted | 11 | 0.095112106 | 0.044773178 | 0.033644238 | 1.09978214 | 1.007383803 | 1.200655352 | 0.999084603 | FALSE |
| chronic periodontitis | X-25172 levels | Simple mode | 11 | 0.093768573 | 0.093038776 | 0.337293374 | 1.098305539 | 0.915223091 | 1.318012045 |  |  |
| chronic periodontitis | X-25172 levels | Weighted mode | 11 | 0.114133006 | 0.074534526 | 0.156701446 | 1.120901201 | 0.968550491 | 1.297216319 |  |  |
| chronic periodontitis | Bilirubin (E,E) levels | MR Egger | 11 | 0.055999664 | 0.092190389 | 0.558574847 | 1.057597328 | 0.882767431 | 1.267051853 |  |  |
| chronic periodontitis | Bilirubin (E,E) levels | Weighted median | 11 | 0.084549087 | 0.05891566 | 0.15126236 | 1.088226261 | 0.969547698 | 1.221431804 |  |  |
| chronic periodontitis | Bilirubin (E,E) levels | Inverse variance weighted | 11 | 0.087785787 | 0.04371018 | 0.044605026 | 1.091754229 | 1.00211607 | 1.189410421 | 0.999084603 | FALSE |
| chronic periodontitis | Bilirubin (E,E) levels | Simple mode | 11 | 0.118693061 | 0.090622701 | 0.219570434 | 1.126024245 | 0.942775181 | 1.344891789 |  |  |
| chronic periodontitis | Bilirubin (E,E) levels | Weighted mode | 11 | 0.111684557 | 0.074220864 | 0.163298037 | 1.118160089 | 0.966776115 | 1.293248732 |  |  |
| chronic periodontitis | Alpha-tocopherol to sulfate ratio | MR Egger | 11 | -0.134884895 | 0.089976521 | 0.168076575 | 0.873816487 | 0.732538869 | 1.042340939 |  |  |
| chronic periodontitis | Alpha-tocopherol to sulfate ratio | Weighted median | 11 | -0.067388087 | 0.060360094 | 0.264236226 | 0.934832335 | 0.830527805 | 1.052236288 |  |  |
| chronic periodontitis | Alpha-tocopherol to sulfate ratio | Inverse variance weighted | 11 | -0.089667681 | 0.04454664 | 0.044125742 | 0.914234953 | 0.837797299 | 0.997646507 | 0.999084603 | FALSE |
| chronic periodontitis | Alpha-tocopherol to sulfate ratio | Simple mode | 11 | -0.106194892 | 0.098274319 | 0.305247916 | 0.899249374 | 0.741698423 | 1.090267166 |  |  |
| chronic periodontitis | Alpha-tocopherol to sulfate ratio | Weighted mode | 11 | -0.076008752 | 0.081485496 | 0.372903069 | 0.926808095 | 0.790001588 | 1.087305721 |  |  |
| chronic periodontitis | Arginine to phosphate ratio | MR Egger | 11 | -0.12191129 | 0.087811396 | 0.198435243 | 0.885226893 | 0.745260374 | 1.051480368 |  |  |
| chronic periodontitis | Arginine to phosphate ratio | Weighted median | 11 | -0.131983097 | 0.060013761 | 0.027862717 | 0.876355808 | 0.779104521 | 0.985746432 |  |  |
| chronic periodontitis | Arginine to phosphate ratio | Inverse variance weighted | 11 | -0.094230226 | 0.043436996 | 0.030055809 | 0.910073216 | 0.835799325 | 0.990947509 | 0.999084603 | FALSE |
| chronic periodontitis | Arginine to phosphate ratio | Simple mode | 11 | -0.151644071 | 0.102481962 | 0.169748904 | 0.859294074 | 0.702922442 | 1.050452029 |  |  |
| chronic periodontitis | Arginine to phosphate ratio | Weighted mode | 11 | -0.156332051 | 0.086988881 | 0.102527048 | 0.855275148 | 0.721206152 | 1.014266971 |  |  |
| chronic periodontitis | Mannose to N-acetylglucosamine to N-acetylgalactosamine ratio | MR Egger | 11 | 0.097509075 | 0.097164294 | 0.34181317 | 1.102421446 | 0.911254606 | 1.333692073 |  |  |
| chronic periodontitis | Mannose to N-acetylglucosamine to N-acetylgalactosamine ratio | Weighted median | 11 | 0.078006896 | 0.062918895 | 0.215049082 | 1.081130114 | 0.955697214 | 1.223025773 |  |  |
| chronic periodontitis | Mannose to N-acetylglucosamine to N-acetylgalactosamine ratio | Inverse variance weighted | 11 | 0.098488779 | 0.045150774 | 0.029158951 | 1.103502022 | 1.010043361 | 1.205608352 | 0.999084603 | FALSE |
| chronic periodontitis | Mannose to N-acetylglucosamine to N-acetylgalactosamine ratio | Simple mode | 11 | 0.005842308 | 0.101453988 | 0.955212998 | 1.005859408 | 0.824475783 | 1.2271472 |  |  |
| chronic periodontitis | Mannose to N-acetylglucosamine to N-acetylgalactosamine ratio | Weighted mode | 11 | 0.04101062 | 0.089798756 | 0.657649683 | 1.041863171 | 0.873720332 | 1.242364206 |  |  |
| chronic periodontitis | Citrulline to ornithine ratio | MR Egger | 11 | -0.003301611 | 0.090911516 | 0.971822544 | 0.996703833 | 0.834028102 | 1.191109183 |  |  |
| chronic periodontitis | Citrulline to ornithine ratio | Weighted median | 11 | -0.11226701 | 0.062344309 | 0.071741037 | 0.89380557 | 0.790996406 | 1.009977279 |  |  |
| chronic periodontitis | Citrulline to ornithine ratio | Inverse variance weighted | 11 | -0.098365572 | 0.045895803 | 0.032094093 | 0.906317519 | 0.828348478 | 0.991625466 | 0.999084603 | FALSE |
| chronic periodontitis | Citrulline to ornithine ratio | Simple mode | 11 | -0.18318159 | 0.098328932 | 0.092068484 | 0.832616947 | 0.686666674 | 1.009588797 |  |  |
| chronic periodontitis | Citrulline to ornithine ratio | Weighted mode | 11 | -0.129757904 | 0.083673821 | 0.152004795 | 0.87830804 | 0.745456428 | 1.034835818 |  |  |
| chronic periodontitis | Phosphate to linoleoyl-arachidonoyl-glycerol (18:2 to 20:4) [2] ratio | MR Egger | 11 | 0.160169444 | 0.106407299 | 0.16652057 | 1.173709733 | 0.952763223 | 1.445893905 |  |  |
| chronic periodontitis | Phosphate to linoleoyl-arachidonoyl-glycerol (18:2 to 20:4) [2] ratio | Weighted median | 11 | 0.093946957 | 0.070865239 | 0.184934837 | 1.098501477 | 0.956046323 | 1.262183082 |  |  |
| chronic periodontitis | Phosphate to linoleoyl-arachidonoyl-glycerol (18:2 to 20:4) [2] ratio | Inverse variance weighted | 11 | 0.115330216 | 0.050603508 | 0.022661401 | 1.12224396 | 1.016278414 | 1.239258345 | 0.999084603 | FALSE |
| chronic periodontitis | Phosphate to linoleoyl-arachidonoyl-glycerol (18:2 to 20:4) [2] ratio | Simple mode | 11 | 0.153124528 | 0.111180519 | 0.198475185 | 1.165470104 | 0.937264935 | 1.449238644 |  |  |
| chronic periodontitis | Phosphate to linoleoyl-arachidonoyl-glycerol (18:2 to 20:4) [2] ratio | Weighted mode | 11 | 0.13918556 | 0.096285661 | 0.178904017 | 1.149337351 | 0.951672481 | 1.388057734 |  |  |
| chronic periodontitis | Retinol (Vitamin A) to linoleoyl-arachidonoyl-glycerol (18:2 to 20:4) [2] ratio | MR Egger | 11 | 0.156719589 | 0.113894305 | 0.202087807 | 1.16966758 | 0.93565052 | 1.462215025 |  |  |
| chronic periodontitis | Retinol (Vitamin A) to linoleoyl-arachidonoyl-glycerol (18:2 to 20:4) [2] ratio | Weighted median | 11 | 0.114431286 | 0.068256678 | 0.093643263 | 1.121235594 | 0.980834241 | 1.281734674 |  |  |
| chronic periodontitis | Retinol (Vitamin A) to linoleoyl-arachidonoyl-glycerol (18:2 to 20:4) [2] ratio | Inverse variance weighted | 11 | 0.121822547 | 0.053820689 | 0.023605551 | 1.129553642 | 1.016468126 | 1.255220303 | 0.999084603 | FALSE |
| chronic periodontitis | Retinol (Vitamin A) to linoleoyl-arachidonoyl-glycerol (18:2 to 20:4) [2] ratio | Simple mode | 11 | 0.160439826 | 0.096812032 | 0.128467519 | 1.174027126 | 0.971113667 | 1.419339197 |  |  |
| chronic periodontitis | Retinol (Vitamin A) to linoleoyl-arachidonoyl-glycerol (18:2 to 20:4) [2] ratio | Weighted mode | 11 | 0.144136056 | 0.088977481 | 0.136318593 | 1.155041248 | 0.970193438 | 1.375107513 |  |  |
| chronic periodontitis | Theophylline to theobromine ratio | MR Egger | 11 | -0.053080613 | 0.091857019 | 0.577527298 | 0.948303564 | 0.792058227 | 1.135370631 |  |  |
| chronic periodontitis | Theophylline to theobromine ratio | Weighted median | 11 | -0.093669774 | 0.062114276 | 0.13154857 | 0.910583411 | 0.806207798 | 1.028472001 |  |  |
| chronic periodontitis | Theophylline to theobromine ratio | Inverse variance weighted | 11 | -0.107229461 | 0.045524402 | 0.018501373 | 0.89831952 | 0.821636423 | 0.982159429 | 0.999084603 | FALSE |
| chronic periodontitis | Theophylline to theobromine ratio | Simple mode | 11 | -0.122340699 | 0.088448604 | 0.196708435 | 0.88484685 | 0.744010625 | 1.05234243 |  |  |
| chronic periodontitis | Theophylline to theobromine ratio | Weighted mode | 11 | -0.100055028 | 0.075901252 | 0.216823291 | 0.904787628 | 0.779719155 | 1.049917329 |  |  |
| chronic periodontitis | Glucose to N-palmitoyl-sphinganine (d18:0 to 16:0) ratio | MR Egger | 11 | 0.079956044 | 0.090749075 | 0.401197735 | 1.083239452 | 0.906728564 | 1.294111333 |  |  |
| chronic periodontitis | Glucose to N-palmitoyl-sphinganine (d18:0 to 16:0) ratio | Weighted median | 11 | 0.123199664 | 0.061767213 | 0.046088678 | 1.13111024 | 1.002138209 | 1.276680565 |  |  |
| chronic periodontitis | Glucose to N-palmitoyl-sphinganine (d18:0 to 16:0) ratio | Inverse variance weighted | 11 | 0.108783491 | 0.044083084 | 0.013598711 | 1.114920934 | 1.022632973 | 1.215537462 | 0.999084603 | FALSE |
| chronic periodontitis | Glucose to N-palmitoyl-sphinganine (d18:0 to 16:0) ratio | Simple mode | 11 | 0.134432518 | 0.091300085 | 0.171667572 | 1.143887464 | 0.956460632 | 1.368042225 |  |  |
| chronic periodontitis | Glucose to N-palmitoyl-sphinganine (d18:0 to 16:0) ratio | Weighted mode | 11 | 0.140883621 | 0.082370442 | 0.117987523 | 1.151290654 | 0.979647521 | 1.353007222 |  |  |
| Abbreviations: IVW, inverse-variance weighting; MR, Mendelian randomization; SNP, single nucleotide polymorphisms; OR, odds ratio; lci, lower confidence interval; uci, upper confidence interval | | | | | | | | | | | |

**Table S7.** Sensitivity analyses based on the causal effects of chronic periodontitis on the blood metabolites.

| **Exposure** | **Outcome** | **Heterogeneity (Cochran's Q)** | | **Pleiotropy** | |
| --- | --- | --- | --- | --- | --- |
|  |  | **IVW_pvalue** | **MR Egger_pvalue** | **Egger_intercept** | **pvalue (MR Egger)** |
| chronic periodontitis | Glycerophosphorylcholine (GPC) levels | 0.979371401 | 0.971375254 | -0.008006155 | 0.612753136 |
| chronic periodontitis | Pyridoxate levels | 0.154313081 | 0.138143373 | 0.014170695 | 0.471872019 |
| chronic periodontitis | N-acetylthreonine levels | 0.555715847 | 0.556341513 | 0.014282895 | 0.350034188 |
| chronic periodontitis | Stachydrine levels | 0.424754531 | 0.438920143 | 0.016819332 | 0.301982193 |
| chronic periodontitis | 3,7-dimethylurate levels | 0.964198293 | 0.979185687 | 0.017022594 | 0.338505189 |
| chronic periodontitis | 1-linoleoyl-GPE (18:2) levels | 0.884038262 | 0.832771198 | -0.004556325 | 0.773925921 |
| chronic periodontitis | Glycerophosphoethanolamine levels | 0.55918155 | 0.477051573 | -0.005710425 | 0.718687801 |
| chronic periodontitis | 4-vinylphenol sulfate levels | 0.165106909 | 0.132638845 | -0.010398133 | 0.59767451 |
| chronic periodontitis | 1-palmitoyl-2-linoleoyl-GPE (16:0/18:2) levels | 0.437917487 | 0.354429162 | 0.004279189 | 0.796604285 |
| chronic periodontitis | Androstenediol (3beta,17beta) disulfate (2) levels | 0.747698222 | 0.667009205 | 0.003045648 | 0.828992557 |
| chronic periodontitis | 16a-hydroxy DHEA 3-sulfate levels | 0.173817678 | 0.231519032 | -0.022309312 | 0.216458148 |
| chronic periodontitis | Cinnamoylglycine levels | 0.455547911 | 0.365553703 | -0.001775823 | 0.914030922 |
| chronic periodontitis | Fructosyllysine levels | 0.146237112 | 0.147772679 | 0.017514307 | 0.375827835 |
| chronic periodontitis | N-palmitoyl-sphinganine (d18:0/16:0) levels | 0.840690312 | 0.802960277 | -0.008949117 | 0.57377392 |
| chronic periodontitis | 1-oleoyl-2-linoleoyl-GPE (18:1/18:2) levels | 0.409525834 | 0.375224326 | 0.012529629 | 0.454478558 |
| chronic periodontitis | Furaneol sulfate levels | 0.58450965 | 0.499190479 | 0.00590181 | 0.754824208 |
| chronic periodontitis | Perfluorooctanesulfonate (PFOS) levels | 0.500042587 | 0.416276108 | -0.004719009 | 0.749835578 |
| chronic periodontitis | Glycosyl ceramide (d18:2/24:1, d18:1/24:2) levels | 0.953918668 | 0.989072601 | -0.019443648 | 0.223458609 |
| chronic periodontitis | Carotene diol (2) levels | 0.249429358 | 0.343479462 | 0.024073189 | 0.171738705 |
| chronic periodontitis | Glyco-beta-muricholate levels | 0.923226096 | 0.89495264 | -0.00816888 | 0.637074368 |
| chronic periodontitis | Ascorbic acid 3-sulfate levels | 0.535217795 | 0.539893827 | 0.015113177 | 0.337969148 |
| chronic periodontitis | 11beta-hydroxyandrosterone glucuronide levels | 0.536248023 | 0.48202411 | -0.009638737 | 0.528707431 |
| chronic periodontitis | 2-hydroxy-4-(methylthio)butanoic acid levels | 0.959380897 | 0.958117549 | -0.011289044 | 0.471759084 |
| chronic periodontitis | N-acetyl-2-aminoadipate levels | 0.472331837 | 0.380241604 | -0.000573233 | 0.973165806 |
| chronic periodontitis | 2-methoxyhydroquinone sulfate (1) levels | 0.892490155 | 0.840904727 | 0.003805214 | 0.817344644 |
| chronic periodontitis | 3-(4-hydroxyphenyl)lactate levels | 0.784341023 | 0.705188883 | 0.001807665 | 0.904245368 |
| chronic periodontitis | Guanidinoacetate levels | 0.322607155 | 0.273134836 | -0.009599359 | 0.570452315 |
| chronic periodontitis | Citrulline levels | 0.275814696 | 0.207057049 | 0.002565817 | 0.889472281 |
| chronic periodontitis | Pyridoxal levels | 0.669441779 | 0.600899948 | -0.007571092 | 0.639282661 |
| chronic periodontitis | X-11381 levels | 0.469662631 | 0.567008774 | -0.021437929 | 0.191367857 |
| chronic periodontitis | X-11795 levels | 0.517790214 | 0.632719428 | -0.021644332 | 0.180367814 |
| chronic periodontitis | X-12026 levels | 0.5033583 | 0.410872885 | -0.001791108 | 0.907107164 |
| chronic periodontitis | X-12818 levels | 0.997394165 | 0.996325843 | -0.008785487 | 0.632956559 |
| chronic periodontitis | X-12906 levels | 0.56170789 | 0.466781194 | -0.001072821 | 0.946173127 |
| chronic periodontitis | X-15461 levels | 0.303826039 | 0.374331137 | -0.021104927 | 0.205026313 |
| chronic periodontitis | X-21364 levels | 0.564477259 | 0.469227128 | -0.000605421 | 0.968063409 |
| chronic periodontitis | X-23587 levels | 0.970229418 | 0.952086964 | -0.005617706 | 0.733344806 |
| chronic periodontitis | X-25172 levels | 0.644781479 | 0.557047805 | -0.00390553 | 0.808220257 |
| chronic periodontitis | Bilirubin (E,E) levels | 0.445646278 | 0.369364397 | 0.006301603 | 0.700932868 |
| chronic periodontitis | Alpha-tocopherol to sulfate ratio | 0.8423268 | 0.804307922 | 0.008982886 | 0.577173558 |
| chronic periodontitis | Arginine to phosphate ratio | 0.784931305 | 0.717735639 | 0.005494832 | 0.725187347 |
| chronic periodontitis | Mannose to N-acetylglucosamine to N-acetylgalactosamine ratio | 0.426803322 | 0.337913946 | 0.000192872 | 0.991024705 |
| chronic periodontitis | Citrulline to ornithine ratio | 0.377817333 | 0.413952729 | -0.018847222 | 0.259805994 |
| chronic periodontitis | Phosphate to linoleoyl-arachidonoyl-glycerol (18:2 to 20:4) [2] ratio | 0.271418896 | 0.21920671 | -0.008893108 | 0.63930832 |
| chronic periodontitis | Retinol (Vitamin A) to linoleoyl-arachidonoyl-glycerol (18:2 to 20:4) [2] ratio | 0.181857434 | 0.136443781 | -0.006920523 | 0.732531661 |
| chronic periodontitis | Theophylline to theobromine ratio | 0.840982343 | 0.814310774 | -0.010775527 | 0.514395774 |
| chronic periodontitis | Glucose to N-palmitoyl-sphinganine (d18:0 to 16:0) ratio | 0.486819992 | 0.405993073 | 0.00572607 | 0.723111997 |
| Abbreviations: IVW, inverse-variance weighting; MR, Mendelian randomization. | | | | | |
